# Probiotics and Extracellular Vesicles as Redox Modulators in Wound Healing: From Microbial Therapeutics to Engineered Nanotherapeutic Strategies

**DOI:** 10.3390/antiox15091172

**Published:** 2026-09-15

**Authors:** Aline Yen Ling Wang, Ana Elena Aviña, Jerry Tsing-Kai Lin, Yen-Yu Liu, Min Hsuan Lin, Huang-Kai Kao

**Affiliations:** 1 Center for Reconstruction, Transplantation and Regeneration Research, Chang Gung Memorial Hospital, No. 5, Fuxing St., Taoyuan 333, Taiwan; aviestlani@hotmail.com (A.E.A.); louis881128@gmail.com (Y.-Y.L.); m9571008@gmail.com (M.H.L.); 2 International PhD Program in Medicine, College of Medicine, Taipei Medical University, Taipei 110, Taiwan; 3 Division of Reconstructive Microsurgery, Department of Plastic Surgery, Chang Gung Memorial Hospital, Taoyuan 333, Taiwan; jerry15862@gmail.com (J.T.-K.L.); kai3488@gmail.com (H.-K.K.); 4 College of Medicine, Chang Gung University, Taoyuan 333, Taiwan

**Keywords:** probiotic-derived extracellular vesicles, wound healing, oxidative stress, redox modulation, reactive oxygen species, probiotics, extracellular vesicles, bioengineering, ROS-responsive therapeutics, regenerative medicine

## Abstract

Chronic wounds are characterized by prolonged inflammation, excess oxidative stress, unregulated angiogenesis, and faulty tissue regeneration that are associated with slow wound healing and poor clinical outcomes. Reactive oxygen species (ROS) signaling deregulation is involved in the pathway of chronic wound pathophysiology, altering redox balance, augmenting the inflammatory response, and inhibiting the cellular response. In this review, a structured literature search and evidence-screening process was used to synthesize evidence on probiotics, EVs, and engineered EVs derived from probiotics in the context of wound healing. Studies suggest that EVs from probiotics may impact redox-sensitive processes of wound healing, including changes associated with Nrf2/HO-1, NF-κB signaling, modulation of mitochondrial ROS, macrophage polarization, and repair of the epithelial barrier. Many of these mechanistic links are derived from pathway-based markers or indirect experiments, however, and will need further mechanistic validation. Probiotic-EVs may offer potential advantages over traditional antioxidant delivery and mammalian-EV systems, including being cell-free, compatible with the microbiome, and easy to engineer. Recent progress in synthetic biology, cargo loading, biomaterial-assisted delivery systems, and ROS-responsive platforms may enable engineered probiotic-EVs to serve as programmable redox nanotherapies. However, major issues regarding EV standardization, biosafety, biodistribution, pharmacokinetics, and clinical validation remain unresolved. Importantly, the evidence for the application of probiotic-derived EVs to wound healing is predominantly preclinical, and there is a lack of direct clinical evidence to date. Collectively, EVs secreted from probiotics might hold potential for chronic wound care and redox-oriented regenerative medicine as a therapeutic platform.

## 1. Introduction

Re-establishment of the integrity of the skin after injury is a basic biological event that is required for tissue homeostasis and to protect the wounds from microbial invasion, dehydration, and chronic inflammation [1,2]. While the majority of acute wounds will go through a well-orchestrated process of wound healing, a significant number of wounds do not heal in a timely fashion but instead turn into chronic wounds that will not heal [3,4]. Chronic wounds like diabetic foot ulcers, venous leg ulcers, and pressure ulcers are growing clinical problems for the aging population and people suffering from metabolic problems like diabetes mellitus and obesity [5,6]. It is known that these wounds are expected to have the following characteristics: prolonged inflammation, recurring infections, abnormal processes of angiogenesis (blood vessel formation), and excessive tissue destruction, which ultimately result in pain, disability, extended hospitalizations, and sometimes limb amputation [5,6]. Although various strategies are available for wound management, including antimicrobial dressings, growth factor therapy, stem cell-based approaches, and bioengineered substitutes, their therapeutic outcomes can be variable, and their regenerative efficacy in the microenvironments of chronic wounds may be constrained by modality-specific limitations, including limited tissue penetration, short biological half-lives, manufacturing complexity and cost, and inconsistent regenerative efficacy [3,4,6]. Consequently, there is growing interest in elucidating novel mechanisms and developing biologically adaptable therapies capable of restoring tissue repair processes under chronic inflammatory-oxidative conditions [3,4,6].

The overproduction and persistence of ROS are considered as major pathological features of chronic wounds. While transient ROS signaling has a role in host defense and normal repair, sustained oxidative stress engenders inflammation, impaired cellular responses, proteolytic activity, and mitochondrial dysfunction, which affect matrix remodeling, re-epithelialization, and angiogenesis [7,8,9,10,11,12,13,14,15,16]. These redox abnormalities are especially pronounced in diabetic wounds in which hyperglycemia, ischemia, and chronic activation of inflammatory cells further add to the oxidative stress. The physiological and pathological functions of ROS in wound healing are extensively described in Section 2.

As oxidative stress plays a pivotal role in the pathogenesis of chronic wounds, a lot of interest has been focused on the therapeutic potential of antioxidants for the restoration of redox homeostasis and the healing of chronic wounds. Conventional antioxidants such as polyphenols, ROS-scavenging small molecules, and vitamins have been found to have beneficial effects in various experimental models of wounds, restoring redox homeostasis and reducing excessive inflammatory responses [7,17,18,19,20]. Some polyphenols like curcumin, quercetin, resveratrol, and genistein have anti-oxidative stress activity and the ability to improve the activities of endogenous anti-oxidative defense systems, and some polyphenols also have the ability to inhibit the activation of redox-sensitive transcription factors (Nrf2 and NF-κB) and reduce chronic inflammation and promote tissue repair [7,17,19]. Small molecule antioxidants and ROS scavengers like N-acetylcysteine and edaravone have been shown to decrease excessive accumulation of ROS and oxidative tissue damage, while vitamins C and E have been shown to play a role in redox homeostasis, not only by scavenging free radicals but also by restoring antioxidant capacity [17,19,20]. Additionally, in diabetic wound models, provision of vitamins C and E has been shown to improve antioxidant enzyme activities, decrease ROS, and promote faster wound healing, further suggesting the therapeutic potential of antioxidant-based interventions [19]. However, their clinical translation has been limited because of their low stability in the wound microenvironment, which has a high amount of protease, their rapid degradation, their poor tissue retention, and their failure to obtain sustained or site-specific redox regulation. Likewise, growth factors, including vascular endothelial growth factor (VEGF), platelet-derived growth factor (PDGF), and epidermal growth factor (EGF), have demonstrated the potential to stimulate angiogenesis and cells, and their topical or local application has also been found to be beneficial; however, these bioactive factors tend to be highly susceptible to enzymatic breakdown and less effective in chronic wounds that have a high inflammatory profile and excessive proteolytic activity [21,22,23]. Over the last few years, cell-based therapies, specifically mesenchymal stem cell (MSC)-based therapies, have become promising therapies in the field of regeneration due to their immunomodulatory and paracrine effects [24,25]. Although the preclinical results look promising, there are still a number of practical and biological stumbling blocks that prevent their widespread use as treatments, such as donor variability, poor cell survival after transplantation, potential immunogenicity, manufacturing costs, and the safety and standardization of the cells. Importantly, there is growing evidence that many of the beneficial effects previously thought to be due to engraftment of the transplanted cells are indeed due to the expression of extracellular factors secreted by the grafts and not to direct engraftment [26,27,28,29,30,31]. All of these properties have forced today’s research focus on less complex and biologically derived delivery systems that allow for the simultaneous modulation of oxidative stress, inflammation, and tissue repair in a more stable, controllable, and clinically translatable way.

EVs are biologically active vehicles of intercellular communication that have become the focus of much attention in the field of emerging acellular therapeutic platforms in relation to the intercellular communication that occurs during tissue repair and inflammatory regulation [26,28]. EVs are nanoscale membrane-enclosed vesicles that can be used for protein, lipid, nucleic acid, metabolite, and redox-regulating molecule transfer to recipient cells and can impact immune modulation, angiogenesis, extracellular matrix (ECM) remodeling, cell stress adaptation, and regenerative signaling. In particular, EVs could be very useful in chronic wounding as they might offer a biologically regulated way of dealing with ROS scavenging and controlling the levels of oxidative stress and the inflammatory response, as opposed to ROS scavenging alone [26,27,32,33]. Although most existing studies on EVs in wound healing have used vesicles generated from mammalian cells, particularly MSCs, EVs from microbes may also be involved in host tissue regulation [29]. In particular, EVs excreted by beneficial probiotic species have become interesting microbiome-derived nanovesicles with potential immunoregulatory and antioxidant properties that could pave the way for new redox-based regenerative therapeutic strategies [27,34].

Given the growing awareness of the potential for probiotic EVs to be involved in the communication of microbes with host cells, these EVs have recently become a more interesting subclass of bioactive nanovesicles that could be utilized in redox signaling and wound healing [27,35]. Probiotic EVs are a cell-free and potentially safer alternative to conventional probiotic therapies where live microorganisms are fed and could convey the functional messages of the microorganisms without the risks of colonization or translocation. It has been increasingly recognized that EVs originating from beneficial bacterial species, like *Lactobacillus*, *Bifidobacterium*, and *Akkermansia*, have a range of bioactive cargo, including proteins, lipids, short-chain fatty acid (SCFA)-associated metabolites, nucleic acids, and immunomodulatory molecules that can affect the responses to oxidative stress, inflammatory signaling, epithelial barrier function, and tissue repair [36,37]. In addition to their inherent biological activity, EVs also represent an appealing basis for engineering approaches for the enhanced delivery of antioxidant cargo and targeting to specific tissues and for the response to the oxidative microenvironments of wounds [38,39]. These properties set the EVs derived from probiotics apart from some related therapeutic platforms. Probiotic-EVs are cell-free, non-replicative, and do not need to maintain the viability of bacteria or colonize to provide the microbial bioactive components, unlike live probiotics. Probiotic-EVs could be regarded as part of a larger scope of postbiotic products, but the membrane-enclosed vesicular structure distinguishes them from non-vesicular postbiotic products like soluble metabolites, secreted molecules, and cell fragments, and allows for the transport of multiple molecular cargoes in a coordinated fashion. Probiotic-EVs, unlike EVs derived from mammalian systems including MSC-derived EVs, are generated from microbial systems and contain components specific to microbiota which may have unique immunomodulatory and redox-associated signaling properties. In contrast to synthetic antioxidant nanocarriers, probiotic-EVs are vesicles of biological origin that contain inherent microbial cargo and could potentially enable the combination of endogenous biological activity with cargo engineering and biomaterial-assisted delivery. These differences are the basis for considering EVs derived from probiotics as a unique emerging platform at the intersection of microbiome-derived therapeutics, redox regulation, and wound repair. Yet, despite the increasing number of studies on probiotics, EV-based therapeutics, and redox regulation in wound healing, the evidence for probiotic-derived EVs is still scattered among microbiome, extracellular vesicle, and regenerative medicine studies. In particular, a critical synthesis linking probiotic-derived EVs with redox-regulatory mechanisms, engineering strategies, wound-repair evidence, and current translational limitations remains limited. Accordingly, this review aims to critically synthesize the current evidence by focusing on: (i) redox imbalance and oxidative stress in chronic wound healing; (ii) antioxidant and redox-regulatory mechanisms associated with probiotics; (iii) probiotic-derived EVs as emerging redox-modulating platforms for wound repair; (iv) engineering strategies and biomaterial-assisted delivery approaches designed to enhance probiotic-EV function and delivery; and (v) the current mechanistic, methodological, safety, and translational challenges that need to be addressed before clinical application. Particular attention is given to the strength and limitations of the available evidence and to distinguishing preclinical mechanistic associations from clinically validated effects.

## 2. Redox Imbalance and Oxidative Stress in Wound Healing

### 2.1. Phases of Wound Healing and Redox Dynamics

The coordinated progression of wound healing occurs through four overlapping phases: hemostasis, inflammation, proliferation, and remodeling, all depending in part on tightly regulated redox signaling [40,41]. Transient ROS generation during hemostasis and inflammation helps maintain antimicrobial defense, recruitment of leukocytes, and redox-sensitive signaling, while controlled ROS signaling during proliferation aids in the activity of fibroblasts and keratinocytes, angiogenesis, and extracellular matrix remodeling [42,43,44,45,46]. Restoring redox homeostasis is coupled with the maturation of the matrix and the repair of the tissue during the subsequent remodeling [41,47]. These redox changes in space and time are essential for normal phase transition, and disruption of this equilibrium can lead to a chronic wound progression of persistent inflammation, proteolysis, and regeneration failure.

### 2.2. Dual Roles of ROS in Wound Healing

In the wound healing process, ROS have pleiotropic and highly context-dependent effects and are now known as “double-edged swords” because of their biological effects, which depend on the concentration, duration, cellular origin, and spatial localization in the microenvironment of the wound [7,48]. In the initial hemostatic/inflammatory response, there is transient ROS production, which is beneficial and an integral part of the innate defense response of wounds. Neutrophils and macrophages respond to invading pathogens and prevent microbial colonization by producing reactive intermediates like superoxide anions, hydrogen peroxide, and others via respiratory burst mechanisms, which involve NADPH oxidase (NOX) [49,50]. Meanwhile, moderate ROS production can serve as a signaling molecule to activate platelets, stimulate secretion of cytokines, and initiate chemotaxis of leukocytes into the wound area, as well as activate redox-sensitive signaling pathways like NF-κB, HIF-1α, MAPK, and Nrf2 that will help coordinate the early inflammatory response and prepare the wound bed for tissue regeneration [9,42,51]. Oxidative signaling is also under control of this process, which helps to change the pro-inflammatory wound environment into a reparative one and regulates macrophage plasticity.

During the proliferation phase of wound healing, there is a gradual increase in the amount of ROS, which continues to help the healing process by stimulating fibroblast activity, keratinocyte migration, angiogenesis, and remodeling of the extracellular matrix. Low levels of hydrogen peroxide (H_2_O_2_) have been seen to promote vascular endothelial growth factor (VEGF) signaling, endothelial cell sprouting, as well as granulation tissue formation, whilst transient ROS generation also plays a role in promoting wound contraction and matrix turnover by regulated modulation of matrix metalloproteinase (MMP) activity [52,53]. In the normal state, these redox-dependent processes are regulated by endogenous antioxidant systems that tightly balance the production of ROS to play a controlled role as signaling molecules instead of indiscriminate molecules of cytotoxicity.

However, when ROS production is too great or prolonged, these normal signaling properties can be transformed into oxidative damage and inflammatory amplification [54,55,56,57,58]. Such a transition is especially relevant for chronic wounds, where ROS production is increased and surpasses the endogenous anti-oxidative capacity of the wound, which can lead to chronic inflammation and poor wound repair. Hence, therapeutic redox targets should be to re-establish redox balance and not to simply inhibit ROS, as physiological ROS signaling is still required for antimicrobial defense, angiogenesis, keratinocyte migration, and tissue repair. This balance could help to decrease the excess of oxidative stress and to keep the physiological redox signals, which are necessary for proper wound healing. Section 2.3 discusses how this pathological redox state occurs.

### 2.3. Oxidative Stress and Healing Failure in Chronic Wounds

Failure to resolve the presence of oxidative stress is a key pathological process in chronic wounds and interferes with the concerted movement of tissue repair processes and ultimately results in a failure to heal. In contrast to acute wounds, the production of ROS is long-lasting and not controlled in chronic wounds, with excessive production of ROS, failure of antioxidant defense, chronic and nonrevolving inflammation, and impaired cellular adaptation [7,46,58,59,60]. This pathological redox change is particularly marked in diabetic wounds, where hyperglycemia, ischemia, advanced glycation end products (AGEs), mitochondrial dysfunction, the presence of bacteria, and persistent infiltration by inflammatory cells all contribute to increased levels of oxidative stress that rise above normal levels of oxidative stress [46,61,62,63]. Of great importance, overproduction of ROS is detrimental not only by causing oxidative damage but also by setting up a cascade of inflammatory reactions, which will not allow a wound to move beyond the inflammatory stage.

Continual activation of neutrophils and pro-inflammatory macrophages results in chronic production of superoxide by NOX enzymes and production of ROS from mitochondria, and reduced levels of antioxidant enzymes such as superoxide dismutase (SOD), catalase, glutathione peroxidase, and Nrf2-mediated cytoprotective responses further exacerbate the capacity of wound tissue to re-establish redox balance [42,64]. Redox-sensitive inflammatory pathways like NF-κB and MAPK or inflammasome-associated signaling pathways can also be over-activated by excessive oxidative signaling, leading to increased production of pro-inflammatory cytokines such as TNF-α, IL-1β, and IL-6 [48,65,66].

Another characteristic of chronic wounds is that they do not undergo the shift from persistent inflammation to a reparative immune response. Overproduction of ROS can hinder the macrophage phenotypic switch and inflammation resolution [67], and chronic hypoxia and vascular insufficiency also raise ROS production from the mitochondria [68]. All of this culminates in a vicious cycle of oxidative stress, inflammation, and ischemia that sustains wound chronicity. In the context of this persistent redox imbalance, the downstream cellular and extracellular matrix consequences are discussed in Section 2.4.

### 2.4. ROS-Induced Cellular Dysfunction and MMP Activation

As oxidative stress continues, the function of reparative cells and ECM homeostasis are progressively impaired, leading to an inability to effectively close the wound [69,70]. While transient ROS signaling is important for physiological proliferation, migration, and tissue remodeling, sustained ROS signaling can lead to senescence, apoptosis, and impaired regenerative activity of fibroblasts, keratinocytes, and endothelial cells [42,71].

Granulation tissue formation, collagen deposition, and extracellular matrix reconstruction are processes that involve high participation of fibroblasts, and for this reason, they are particularly vulnerable [72,73]. A high level of ROS leads to a loss of integrity of the mitochondrial membranes, which leads to a reduction in ATP generation and an increase in the expression of oxidative stress-associated apoptotic pathways, ultimately resulting in a decrease in fibroblast proliferation, migration, and collagen production [55,74,75]. Chronic oxidative damage also disrupts the responsiveness to TGF-β and the differentiation of fibroblasts into myofibroblasts, which impairs wound contraction and matrix remodeling [76,77]. Likewise, ROS exposure delays keratinocyte proliferation and re-epithelialization by damaging mitochondria and disrupting the cytoskeleton, or by causing a lack of responsiveness to growth signals [78,79]. Endothelial cells are also very sensitive to oxidant damage, leading to decreased angiogenic signaling and reduced nitric oxide (NO) bioavailability, endothelial dysfunction, and impaired neovascularization, all of which further contribute to tissue hypoxia [80,81].

Along with cellular dysfunction, overabundance of ROS also plays important roles in the pathological degradation of the extracellular matrix; the accumulation of various kinds of matrix metalloproteinases (MMPs) is a characteristic feature of chronic non-healing wounds [7,82]. In normal circumstances, MMP activity is tightly controlled and is involved in controlled ECM turnover and tissue remodeling. However, chronic exposure to oxidative stress leads to imbalances between the MMPs and the natural inhibitors of the MMPs, such as the tissue inhibitors of metalloproteinases (TIMPs) and creates a very proteolytic wound environment [83,84]. ROS have been shown to activate the transcription of various MMPs (MMP-1, MMP-2, MMP-8, and MMP-9) through redox-sensitive signaling pathways including NF-κB and AP-1 and to downregulate TIMP expression [42,85]. High protease activity degrades collagen, fibronectin, laminin, and other structural matrix proteins needed for the integrity of the scaffold and anchorage of the cells [86,87]. Additionally, MMP overactivation can also destabilize crucial growth factors like VEGF, PDGF, EGF, and TGF-β, which are necessary for regenerative signaling mechanisms related to the processes of angiogenesis, epithelial repair, and matrix reconstruction [88,89].

ROS-induced dysfunction of reparative cells, combined with dysregulated MMP activity, results in failure of tissue regeneration and ECM integrity, further contributing to persistent wound-healing failure [7,44,46,60]. The redox-dependent pathological processes represent the mechanistic ground for the establishment of a probiotic approach to chronic wound repair. Specifically, probiotics and EVs derived from probiotics have been linked to modulation of inflammatory signaling, macrophage response, oxidative stress, and endogenous antioxidant mechanisms. Therefore, rather than functioning solely as ROS-scavenging agents, probiotic-derived EVs may represent a potential strategy for modulating the dysregulated redox microenvironment of chronic wounds. The available evidence regarding these effects and the limitations of the mechanism are discussed in the following sections.

## 3. Antioxidant and Redox-Regulatory Roles of Probiotics in Wound Healing

### 3.1. Literature Screening and Study Classification

With oxidative stress being a main factor in chronic wound pathologies, more recent research has focused on understanding the biological activity of antioxidant agents that can modulate ROS accumulation, restore redox homeostasis, and promote regenerative repair. Of all these new strategies, one that has received significant attention is probiotics due to their characteristics of antioxidant potency, immunomodulatory activity, and barrier protection. There is increasing evidence that various species of probiotics interact with the wound associated oxidative microenvironment in a number of ways, such as direct scavenging of ROS, increasing the activity of the innate antioxidant defense system, regulating the production of inflammatory cytokines, changing the polarization of macrophages, and reducing the activity of proteases which damage the tissue, as summarized in Table 1. Concurrently, the developments in microbial engineering and synthetic biology have further paved the way for a greater therapeutic potential for probiotics, whereby their antioxidant activity can be targeted, bioactive molecules can be released in a controlled manner, and tissue-specific regenerative activity can be enhanced [90,91]. Furthermore, emerging evidence suggests that EVs derived from probiotics might have their own roles in redox regulation and tissue repair via cell-free intercellular signaling mechanisms [92,93,94,95,96]. Taken together, all this evidence points towards the new notion of probiotic treatment not only as a modulator of the microbiome but also as an active modulator of oxidative signaling during wound healing.

**Figure 1:**
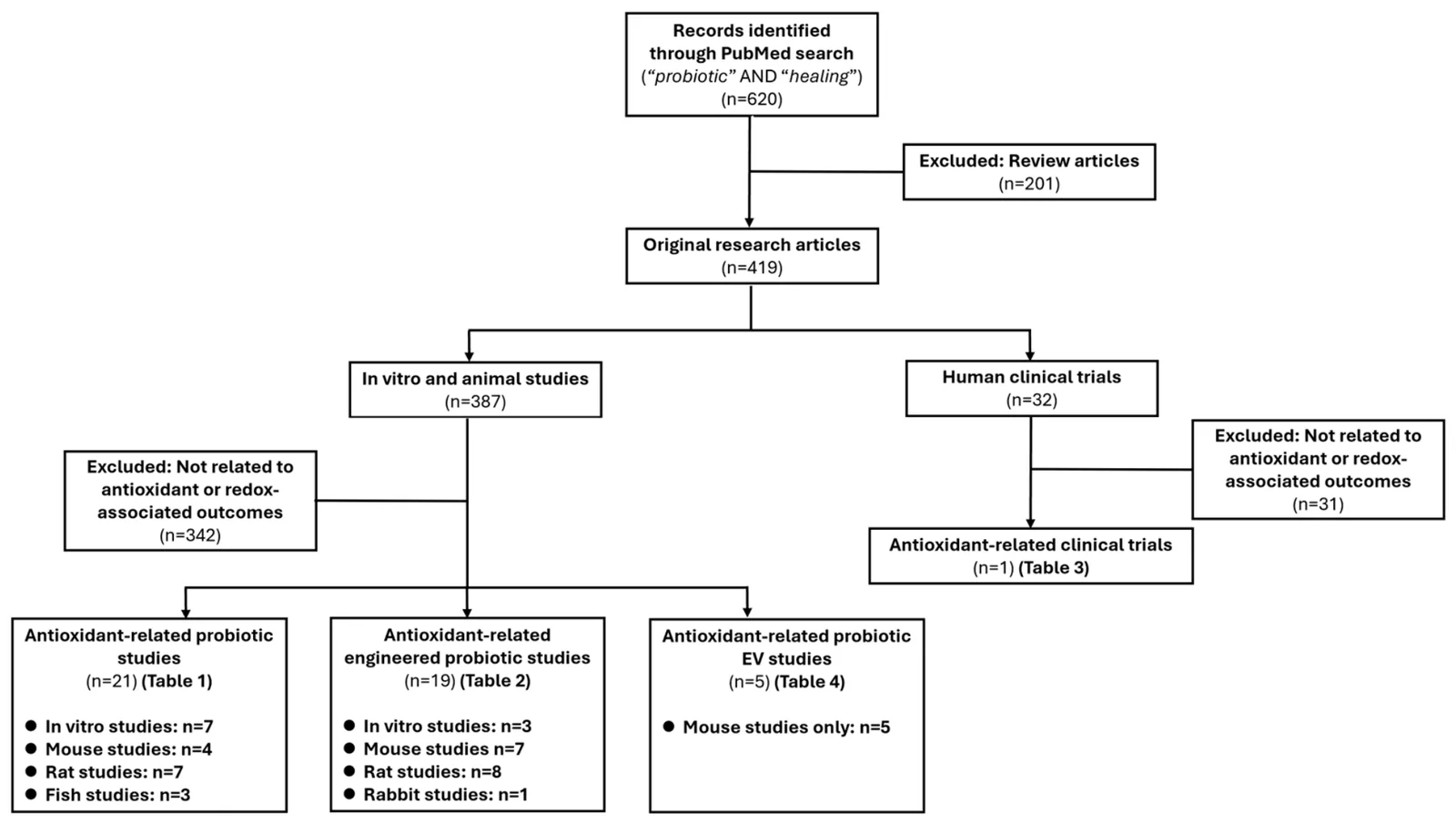
Literature screening and study classification workflow for probiotic-based antioxidant and redox-regulatory strategies in wound healing. A structured PubMed literature search was conducted for studies available through May 2026. Following exclusion of review articles, original research studies were screened based on titles and abstracts and, when necessary, full-text content for antioxidant or redox-associated outcomes relevant to wound healing. Eligible studies were classified into intrinsic probiotic studies, engineered probiotic studies, probiotic-derived extracellular vesicle (EV) studies, and antioxidant-related human clinical studies. Detailed screening criteria and the study classification process are described in Section 3.1.

To evaluate the evidence regarding the antioxidant effects of probiotics in the process of wound healing, a structured literature search and screening process was carried out (Figure 1). The literature search was performed in PubMed, and studies that were published until May 2026 were searched. A preliminary PubMed search was performed using the search terms “probiotic” AND “healing”, which yielded 620 records. A total of 419 original research articles (including in vitro studies, animal experiments, and human clinical studies) were screened after excluding 201 review articles. The 419 original research articles were further screened based on their titles and abstracts and, when necessary, full-text content. Studies were deemed relevant when they reported antioxidant or redox-associated outcomes related to wound healing, such as antioxidant activity, ROS regulation, oxidative stress, or related immunoregulatory responses such as IL-10. If the studies did not have such outcomes, they were not included in the subsequent evidence synthesis. Although IL-10 was not assessed as a direct antioxidant outcome in this review, it was included as an immune-associated outcome due to its reported relationship with inflammatory resolution and redox homeostasis during wound healing [97,98]. Recent studies have also suggested that the increase in IL-10 induced by probiotics can be accompanied by a reduction in oxidative damage, macrophage polarization, and activation of antioxidant mechanisms [99,100,101,102,103]. Accordingly, IL-10-related findings were interpreted as indirect immune-associated evidence rather than direct evidence of antioxidant activity. An additional step of manual checking was performed, where two authors independently checked and confirmed the eligibility of original studies relevant to the scope of this review. Disagreements on study inclusion or classification were discussed and resolved by consensus between the two authors. Included studies were then categorized as per the effects reported in the studies on redox regulation, oxidative signaling pathways, immune regulation (IL-10-associated regulation), and overall wound healing outcomes (Table 1, Table 2, Table 3 and Table 4). The eligible studies were then classified based on the characteristics of the therapeutic modality and the mechanistic aspects. These included intrinsic probiotic antioxidant studies where naturally occurring antioxidant activity of probiotic strains was studied (Table 1; *n* = 21), engineered probiotic studies where the antioxidant efficacy was enhanced using genetic, biomaterial, and/or functional modification of probiotics (Table 2; *n* = 19), probiotic-derived extracellular vesicle studies where EVs produced by probiotics were studied for redox regulation functions (Table 4; *n* = 5), and human clinical studies in which antioxidant-associated outcomes in wound healing studies were reported (Table 3; *n* = 1). The intrinsic and engineered probiotic studies related to the detailed description of mechanisms are also summarized in the Supplementary Tables S1 and S2, respectively, to give a detailed description of the mechanisms from a compendious perspective. A structured screening and classification process was utilized to sort the available evidence for this narrative review and not to perform a formal systematic review or meta-analysis. The results of this evidence synthesis not only pinpoint the fast-growing field of probiotic-based redox therapeutics but also offer a guide for understanding of potential perturbations of oxidative signaling pathways by probiotics and their derivatives at various stages in the wound healing process.

### 3.2. Intrinsic Antioxidant Properties of Probiotics in Wound Repair

The non-engineered probiotic-associated studies included here examined a number of different therapeutic modalities which should not be assumed to be mechanistically equivalent. For clarity, the evidence in this section is distinguished into: (i) live probiotics; (ii) heat-killed or otherwise inactivated probiotics (paraprobiotics); (iii) postbiotic preparations containing inanimate microbial cells and/or their components; (iv) purified microbial products such as exopolysaccharides (EPSs); and (v) cell-free conditioned media or culture supernatants. In addition, engineered live-probiotic systems and EVs derived from probiotics are discussed in separate sections (Section 3.3 and Section 5, respectively) as they are membrane-enclosed vesicles with distinct isolation and characterization considerations, cargo, manufacturing, and safety concerns. For this reason, these categories are discussed individually in this review as they have distinct biological components, modes of action, production needs, safety issues, and possible regulatory categorization. Among these non-engineered probiotic-associated studies, several probiotic strains were shown to exhibit antioxidative and redox-regulatory properties in various types of wound models such as keratinocyte and fibroblast scratch assays, oxidative stress-induced cellular injury, diabetic, infected, burn, oral ulcer, and tissue regeneration models (Table 1; Supplementary Table S1). Overall, these studies indicate that probiotics can influence the level of oxidative stress associated with wounds through a variety of overlapping pathways, such as directly scavenging ROS, boosting the activity of the endogenous antioxidant enzyme systems, modulating redox-sensitive signaling pathways, inhibiting inflammasome activation, and regulating anti-inflammatory cytokines including IL-10. *Lactiplantibacillus plantarum* was the most widely studied probiotic species investigated, which presumably possesses multifunctional antioxidant and regenerative activities both in vitro and in vivo. Mechanistic information for all studies included is summarized in detail in Supplementary Table S1.

Concerning redox modulation, a range of studies showed direct mitigation of the oxidative stress by using probiotics, which reduced the accumulation of ROS in the cells and/or improved their own antioxidant power. Several non-living or cell-free probiotic-associated modalities, including culture supernatants, purified EPS, lysate/inactivated preparations, and postbiotic preparations derived from *L. plantarum*, showed good inhibitory capacity in the DPPH radical-scavenging, reducing-power, metal-chelation, or antioxidant-defense-system-restoring assays. Dubey et al. found, for instance, that the cell-free supernatant of *L. plantarum* could be used topically for the protection of epithelial cells against oxidative stress caused by H_2_O_2_ and simultaneously promote wound closure, fibroblast proliferation, collagen deposition, and angiogenesis in wounds in mice [99]. Elmansy et al. reported strong ROS-scavenging and antioxidant activities of the purified/isolated EPS from *L. plantarum* in burn wounds in rats, along with increased granulation tissue formation and vascularization [104]. Further studies also revealed that probiotics have the potential to enhance the endogenous antioxidant defense, either through the upregulation of antioxidant enzyme (SOD, catalase, GPx, HO-1) expression or the activity of certain cytoprotective genes. A probiotic-metabolite-associated conditioned-medium approach, in which MSCs were preconditioned with probiotic metabolites, for example, has also been shown to enhance the expression of SOD-1, HO-1, and metallothionein-1 and significantly promote the survival of MSCs under oxidative stress conditions [105]. What was interesting was that not all probiotic-mediated ROS regulation was about simply suppressing ROS. *L. rhamnosus* was able to restore ROS that were impaired by exposure to oxytetracycline in a zebrafish regeneration model, thereby promoting the regenerative Wnt signaling and tissue repair [106]. Together, these data indicate that probiotics might not be just ROS scavengers but can act as redox homeostasis modulators able to re-establish redox signaling in a context-dependent manner, thus facilitating the regenerative healing process.

At the signaling pathway level, recent studies have shown that probiotics have an effect on several redox-sensitive molecular pathways that are involved in the processes of inflammation, pyroptosis, angiogenesis, and ECM remodeling. The Nrf2 antioxidant axis and inflammasome-associated signaling were among the several pathways that were reported to be regulated by them [107,108]. Fusco et al. showed that *L. plantarum* rescued the expression of Nrf2 in keratinocytes that were treated with Malassezia furfur and inhibited the activation of AhR/CYP1A1 signaling and the NLRP3 inflammasome, resulting in a decrease in the production of IL-1β and IL-18, and in an improvement in the integrity of the epithelial barrier [107]. In a similar manner, Wang et al. demonstrated that the application of *L. plantarum* to diabetic wounds inhibited NLRP3, caspase-1, and gasdermin D activity, which reduced pyroptosis and accelerated wound healing [108]. In other studies, probiotic activation of signaling pathways for regeneration was observed, such as TGF-β/Smad, HO-1, NOS2/NO, Wnt/β-catenin, and FAK/AKT/ERK signaling. For instance, Demir et al. showed that the EPS produced by *L. plantarum* stimulated the activation of TGF-β1/Smad2/3/4 signaling and expression of the genes related to collagen, which promoted the proliferation of fibroblasts and the formation of new blood vessels (angiogenesis) [109]. In the meantime, Lombardi et al. found that with several probiotic lysates, re-epithelialization was stimulated by the production of NO by NOS2, with strain-dependent differences in the healing efficacy [110]. These results suggest that the redox-modifying effects of probiotics are heterogeneous and possibly strain-specific, dependent on the modality of delivery, and the context of wounds.

Another major mechanistic theme in the papers that were included in this review was that of immune modulation—especially in terms of IL-10-related responses and macrophage polarization. The supplementation of probiotics was reported to upregulate the expression of IL-10 and downregulate pro-inflammatory mediators like IL-1β, IL-6, TNF-α, and IL-18 by multiple probiotic strains, including *L. plantarum*, *B. bifidum*, and *S. putrefaciens* [100,101,102,111]. Interestingly, Ishi et al. found that heat-killed *L. plantarum* (a paraprobiotic preparation) induced the polarization of macrophages to the M2 type and the production of IL-10 via pathways involving CARD9, indicating that non-viable parts of probiotics have the ability to induce a powerful immunoregulatory response without requiring bacterial colonization [112]. In a similar way, Li et al. found that the same B. bifidum-embedded hydrogels promoted the production of IL-10 and the M2 polarization of macrophages and at the same time decreased the production of TNF-α and IL-6 in oral ulcers [111]. These findings are especially relevant when considering chronic wounds, where chronic and ongoing inflammation and oxidative stress in a predominantly M1 response to the wound are thought to be significant factors in impaired healing. The finding that probiotics promote an anti-inflammatory response related to IL-10 levels further reinforces the idea that redox regulation is closely linked with the immunomodulatory response in probiotic wound healing mechanisms.

However, there are several key evidence gaps that need to be overcome. First, there are considerable variations between the probiotics used, the method of administration, wound models, and the mechanistic endpoints; therefore, it is difficult to directly compare the results of different studies. While most studies were based on acute wound or short-term experimental models, clinically relevant chronic wounds are characterized by ischemia, biofilm, metabolic abnormalities, and inflammatory stress. Additionally, there have been multiple reports measuring antioxidant activity in general, using indirect methods that are typically DPPH scavenging or total antioxidant capacity (TAC), with a lack of assessment of intracellular redox dynamics and/or long-term tissue remodeling outcomes [100,104,105,113,114,115,116,117]. Mechanistic validation has also not been adequate in many studies, and several pathways were proposed based on correlative cytokine changes and not on pathway inhibition and/or genetic validation experiments. Importantly, the effect of probiotics seems to be strain-specific and reliant upon the context. Some probiotic lysates boosted the migration of keratinocytes, whereas some inhibited healing processes, as shown by Lombardi et al., although some of the bacteria they belong to are close relatives. Similarly, some ROS restoration might be good for signaling in regeneration, but over-suppression of ROS might, in theory, reduce the physiological ROS-dependent antimicrobial defense and repair processes. So, probiotics should not be regarded as just a sort of universal antioxidant, but as dynamic redox and immune homeostasis modulators, with a possible therapeutic efficacy that requires detailed control of the strains, dosages, vehicles, and microenvironmental conditions of a wound.

**Table 1:** Summary of intrinsic antioxidant mechanisms of probiotics in wound healing.

Probiotic	Model	Redox Modulation	Signaling Pathway	Immune Modulation	Wound Healing	Reference
*L. plantarum*	In vitro: epithelial cell scratch and H_2_O_2_-induced oxidative stress; In vivo: mouse skin wound model	Oxidative stress protection ↑; cell viability ↑ (60–100% protection)	NR	IL-6 ↑ (early phase); IL-10 ↑ (late phase); inflammation modulation (early pro-inflammatory → later anti-inflammatory transition)	↑	Dubey et al., 2021 [99]
*L. plantarum*	Rat burn wound model	ROS scavenging ↑ (DPPH activity ↑); metal chelation ↑; reducing power ↑ (antioxidant capacity ↑)	NR	Inflammation/infiltration ↓	↑	Elmansy et al., 2022 [104]
*L. plantarum*	In vitro keratinocyte model with *M. furfur* infection	Nrf2 ↑ (restored from downregulation induced by M. furfur); ROS-related signaling indirectly modulated via AhR pathway	AhR ↓; CYP1A1 ↓; Nrf2 ↑; NLRP3 inflammasome ↓ (Caspase-1 ↓; IL-1β ↓; IL-18 ↓)	IL-1β ↓; IL-18 ↓; Inflammation ↓; Inflammasome activation ↓	↑	Fusco et al., 2023 [107]
*L. plantarum*	Mouse skin wound model	NR	CARD9-mediated signaling ↑; NF-κB–related pathway (via C-type lectin receptors) involved; MyD88-independent	M2 polarization ↑; IL-10 ↑; chemokines ↑ (CCL5, CXCL1, CXCL2); leukocyte infiltration ↑	↑	Ishi et al., 2023 [112]
*L. plantarum*	Mouse infected skin wound model	Free radical scavenging ↑ (DPPH assay, up to ~90% inhibition); ROS ↓	NR	IL-6 ↓; IL-10 ↑; inflammation ↓		Dubey et al., 2023 [100]
*L. plantarum*	In vitro: HUVEC scratch wound model; In vivo: rat diabetic skin wound model	NR	NLRP3 inflammasome ↓; Caspase-1 ↓; GSDMD ↓ (pyroptosis ↓)	IL-1β ↓; IL-18 ↓; inflammation ↓; pyroptosis ↓	↑	Wang et al., 2024 [108]
*L. plantarum*	Rat diabetic infected skin wound model	Antioxidant compounds present (GC-MS)	NR	IL-10 ↑; IL-6 ↓; inflammation ↓	↑	Narang et al., 2026 [101]
*L. plantarum*	In vitro: fibroblast scratch wound model; in ovo CAM angiogenesis model	NR	TGF-β1/Smad2/3/4 ↑	IL-1β ↓; IL-6 ↓; iNOS ↓ (anti-inflammatory)		Demir et al., 2025 [109]
*L. rhamnosus*	Zebrafish caudal fin amputation model under oxytetracycline exposure	ROS ↑ (restored amputation-induced ROS production)	Wnt signaling ↑ (wnt3a ↑; lef1 ↑; axin2 ↑)	Neutrophil recruitment ↓; apoptosis ↓; inflammation modulation ↑	↑	Wang et al., 2023 [106]
*L. rhamnosus*	In vitro HaCaT scratch wound under TNF-α-induced inflammatory condition	SOD-like activity ↑; DPPH radical scavenging ↑; oxidative stress protection ↑ (H_2_O_2_-induced cy totoxicity ↓)	A2A receptor-mediated FAK ↑/AKT ↑; ERK ↑; p38 ↑	Bidirectional NO regulation: NO ↑ (basal condition); NO ↓ (under LPS stimulation); anti-inflammatory effect ↑	↑	Chae et al., 2025 [113]
*L. reuteri*	Rat skin wound model	MPO ↓ (oxidative/inflammatory enzyme ↓); antioxidant effect↑	NR	Inflammation ↓; neutrophil activity ↓ (via MPO ↓)	↑	Khodaii et al., 2019 [118]
*Lactobacillus* sp. *Ca6*	Rat skin wound model	DPPH radical scavenging ↑; Reducing power ↑; β-carotene bleaching inhibition ↑; Metal chelating activity ↑	NR	Inflammation ↓	↑	Trabelsi et al., 2017 [114]
*B. bifidum*	Rat peptic ulcer wound model	SOD ↑; GPx ↑; antioxidant capacity ↑	NR	IL-8 ↑; neutrophil recruitment ↑; macrophage infiltration ↑	↑	Almasyan et al., 2023 [119]
*B. bifidum*	Rat oral ulcer model	NR	TLR2/TGF-β1/Smad3 (suggested, not directly validated); NF-κB ↓ (indirect evidence via TNF-α, IL-6 reduction)	TNF-α ↓; IL-6 ↓; IL-10 ↑; M2 polarization ↑; inflammation ↓	↑	Li et al., 2026 [111]
*E. mundtii*	In vitro fibroblast scratch wound model	Antioxidant activity ↑ (DPPH scavenging ~40%); ROS ↓	NR	NR	↑	Fidan et al., 2023 [115]
*L. mesenteroides*	In vitro keratinocyte infected scratch wound model	Oxidative stress index ↓; TAC ↑; ROS ↓	NR	IL-6 ↓; inflammation ↓	↑	Altves et al., 2024 [116]
*S. putrefaciens*	Fish skin wound model	SOD ↑; CAT ↑; GSR ↔; peroxidase activity ↑; antioxidant system ↑	NR	IL-10 ↑; TGF-β1 ↑; IL-1β ↓; IL-8 ↓; inflammation ↓	↑	Chen et al., 2020 [102]
*L. casei*, *L. plantarum*	In vitro: H_2_O_2_-induced oxidative stress; In vivo: mouse skin wound model	ROS scavenging ↑ (DPPH assay); MSC survival under oxidative stress (~4–5×) ↑; antioxidant genes ↑ (SOD-1 ↑, HO-1 ↑, MT-1 ↑)	HO-1 ↑; antioxidant gene upregulation (SOD-1, MT-1	Inflammation ↓	↑	Kazemi et al., 2022 [105]
*L. casei*, *L. plantarum*, *multiple Lactobacilli* spp.	In vitro fibroblast scratch wound model	ROS ↓ (DPPH scavenging ↑up to ~60%)	MMP-1/2/3/9/10 ↓; TIMP-1/2 ↑	NR	↑	Shirzad et al., 2018 [117]
*L. casei, L. gasseri, L. paracasei, B. lactis, S. thermophilus*	In vitro: fibroblast scratch and H_2_O_2_-induced oxidative stress	ROS ↓; oxidative damage ↓; cell viability ↑ under oxidative stress	NR	IL-6 ↓; IL-1β ↓; inflammation ↓	↑	Michels et al., 2023 [120]
*S. thermophilus*, *L. plantarum*, *L. acidophilus*, *B. longum*, *B. infantis*, *B. breve*, *L. bulgaricus*	In vitro keratinocyte scratch wound model	NO production ↑ (nitrite levels ↑ via NOS2 activity)	NOS2/NO pathway ↑; NOS2 expression ↑; (effect abolished by NOS2 inhibitor, aminoguanidine)	IL-6 ↑; IL-8 ↑ (associated with NOS2 activation	↑	Lombardi et al., 2019 [110]

**↑**: increase; **↓**: decrease; **↔**: no significant change; →: a transition from one state to another; NR: not reported. *B. bifidum*: *Bifidobacterium bifidum*; *B. breve*: *Bifidobacterium breve*; *B. infantis*: *Bifidobacterium infantis*; *B. lactis*: *Bifidobacterium lactis*; *B. longum*: *Bifidobacterium longum*; *E. mundtii*: *Enterococcus mundtii*; *L. acidophilus*: *Lactobacillus acidophilus*; *L. bulgaricus*: *Lactobacillus bulgaricus*; *L. casei*: *Lactobacillus casei* or *Lacticaseibacillus casei*; *L. gasseri*: *Lactobacillus gasseri*; *L. mesenteroides*: *Leuconostoc mesenteroides*; *L. paracasei*: *Lactobacillus paracasei*; *L. plantarum*: *Lactobacillus plantarum* or *Lactiplantibacillus plantarum*; *L. reuteri*: *Lactobacillus reuteri* or *Limosilactobacillus reuteri*; *L. rhamnosus*: *Lactobacillus rhamnosus* or *Lacticaseibacillus rhamnosus*; *M. furfur*: *Malassezia furfur*; *S. putrefaciens*: *Shewanella putrefaciens*; *S. thermophilus*: *Streptococcus thermophilus*. AhR: aryl hydrocarbon receptor; AKT: protein kinase B; CAM: chick chorioallantoic membrane; CARD9: caspase recruitment domain family member 9; CAT: catalase; COL1A1: collagen type 1 alpha 1; CYP1A1: cytochrome P450 family 1 subfamily A member 1; DPPH: 2,2-diphenyl-1-picrylhydrazyl; ECM: extracellular matrix; ERK: extracellular signal-regulated kinase; FAK: focal adhesion kinase; fn: fibronectin; FOS: fructooligosaccharide; GC-MS: gas chromatography-mass spectrometry; GPx: glutathione peroxidase; GSDMD: gasdermin D; GSR: glutathione reductase; HUVECs: human umbilical vein endothelial cells; LPS: lipopolysaccharide; M2: macrophage M2; MDA: malondialdehyde; MRSA: methicillin-resistant Staphylococcus aureus; NHDF: normal human dermal fibroblasts; NLRP3: NOD-like receptor family, pyrin domain containing 3; Nrf2: nuclear factor erythroid 2–related factor 2; Reuterin: *L. reuteri* -derived metabolite; ROS: reactive oxygen species; shh: sonic hedgehog; SOD: superoxide dismutase. Detailed information for Table 1 is provided in Supplementary Table S1.

**Figure 2:**
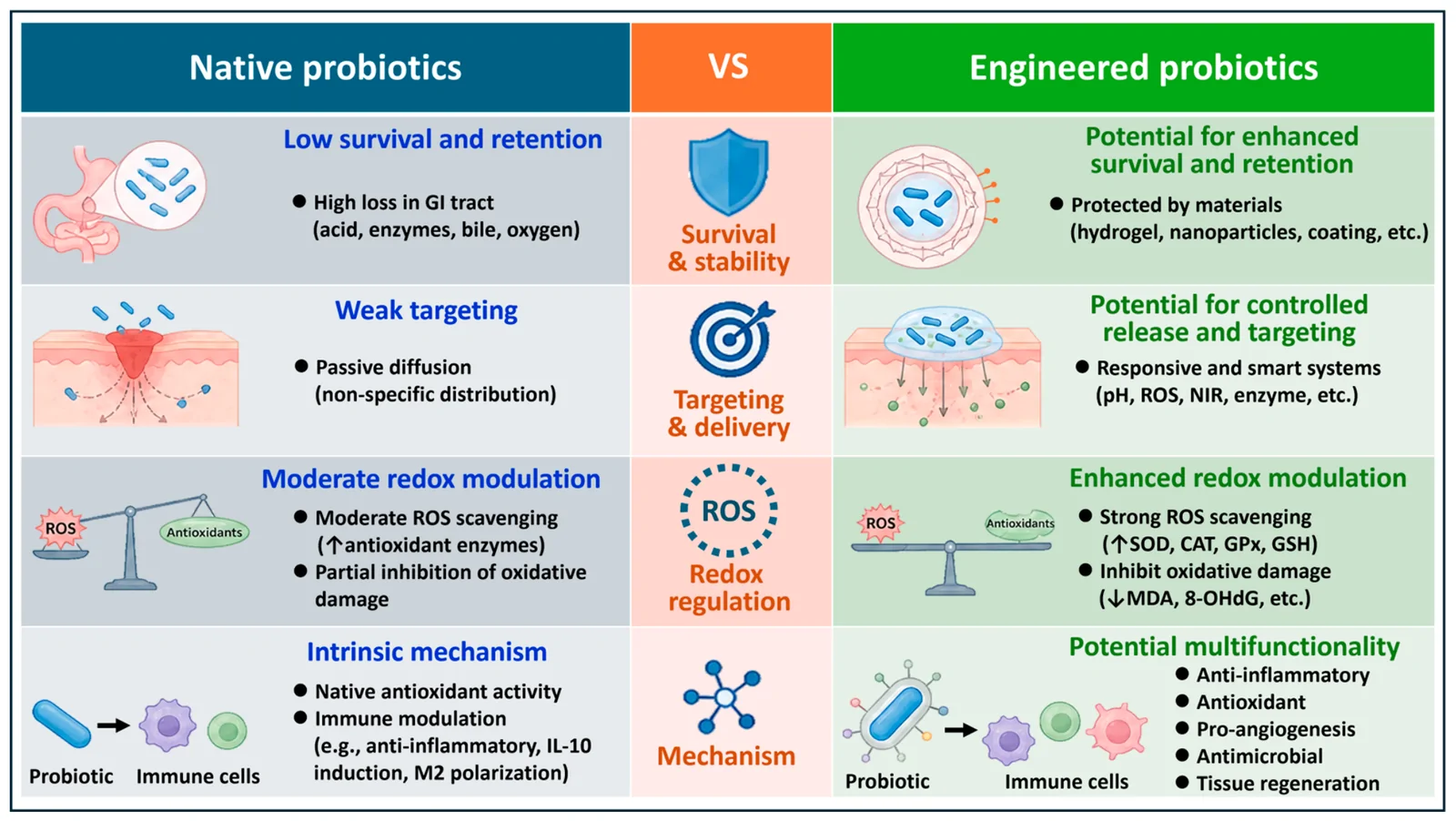
Native live probiotics versus engineered live probiotics for redox regulation and wound healing. Schematic comparison of native probiotics and engineered probiotic platforms in redox-oriented wound therapy across four major dimensions: survival and stability, targeting and delivery, redox regulation, and therapeutic mechanisms. Native probiotics possess intrinsic antioxidant and immunomodulatory activities; however, their therapeutic performance may be limited by low survival and retention under adverse physiological conditions, passive and non-specific distribution, and restricted control over bioactive factor delivery. Consequently, native probiotics generally provide moderate redox modulation through reactive oxygen species (ROS) scavenging, enhancement of endogenous antioxidant defenses, partial inhibition of oxidative damage, and intrinsic immune modulation, including anti-inflammatory responses, IL-10 induction, and M2 macrophage polarization. In contrast, engineered probiotic platforms have the potential to enhance survival and retention through protective biomaterials, hydrogels, nanoparticles, or coatings, and to provide controlled release and targeting through stimuli-responsive systems triggered by cues such as pH, ROS, near-infrared (NIR) irradiation, or enzymatic activity. Engineered probiotics may also provide enhanced redox modulation through stronger ROS-scavenging and antioxidant activities and reduced oxidative damage. In addition, their potential multifunctionality may enable the integration of anti-inflammatory, antioxidant, pro-angiogenic, antimicrobial, and tissue-regenerative functions within a single therapeutic platform. Collectively, these engineering strategies may enable more programmable and multifunctional redox-oriented wound therapies; however, many of these proposed advantages remain primarily supported by preclinical evidence and require further validation in clinically relevant chronic wound models.

Many limitations have been noted in the above-mentioned intrinsic probiotic studies, and this has led to more focus being directed towards engineering probiotic platforms to have additional therapeutic capacity. The key contrasting features of native live probiotics and engineered live-probiotic platforms are illustrated in Figure 2. This is a comparison of viable probiotic systems, and does not mean that heat-killed preparations, postbiotics, EPS, conditioned media/supernatants, or probiotic-derived EVs are mechanistically equivalent to live probiotics. While the inherent antioxidative and immunomodulatory effects of native probiotics are highly beneficial, their therapeutic benefits are limited by a number of biological and translational drawbacks, such as limited survival under unfavorable physiological conditions, low rate of colonization, weak targeting of tissues, and poor control over the delivery of bioactive molecules [27]. In many traditional probiotic strategies, however, the distribution of bacteria within the wound microenvironment has been largely passive and nonspecific, leading to inconsistent therapeutic outcomes and only modestly controlling oxidative stress. Furthermore, the typical mode of action of native probiotics consists of quite simple mechanisms of action, such as native antioxidant activity, short-term immune modulation, or indirect effects via the modulation of the microbiome. Native probiotics are still being poorly translated into clinical practice for chronic wound management, due to stability issues, difficulties in wound retention, and lack of control of redox levels.

To address these disadvantages, there has been a growing interest in the construction of engineered probiotics with a greater number of desirable features such as stability, precision, redox control, and multifunctional therapeutic efficacy [27]. Much like in Figure 2, engineered probiotics are often combined with biomaterials, hydrogels, nanoparticles, or responsive delivery systems that facilitate enhanced protection of the bacteria, retention in the tissues, and controlled release in the oxidative wound microenvironments. Engineered systems, in contrast to passive delivery of native probiotics, could potentially allow for more precise spatial and temporal control of therapeutic release activated by ROS, pH, inflammation, or enzymes [121,122]. In addition, engineered probiotics can be functionalized to secrete antioxidant enzymes, release redox-active metabolites, regulate inflammatory signaling pathways, or function synergistically in a single platform to have antimicrobial, angiogenic, immunomodulatory, and regenerative properties [123,124]. This progress collectively takes the probiotic therapeutic approach to wound healing from relatively simple microbiome modulation to programmable and multifunctional redox therapies, paving the way for new probiotic engineering approaches for wound healing.

### 3.3. Engineering Strategies for Probiotic Redox Modulation in Wound Repair

In this section, “engineered probiotics” are defined as viable probiotic micro-organisms that are genetically, materially, and/or functionally modified or incorporated into engineered delivery platforms, excluding engineered probiotic-derived EVs, which are discussed separately in Section 6. To overcome the drawbacks of using native probiotic bacteria, an increasing amount of research is starting to focus on these engineered probiotic approaches to boost the stability, redox-regulatory activity, targeted delivery, and multifunctional therapeutic activity of probiotics for wound healing (Table 2; Supplementary Table S2). Altogether, these studies illustrate that via the incorporation of biomaterials, nanotechnology, responsive delivery platforms, and multifunctional bioactive components, the function and performance of conventional probiotics in terms of antioxidative and regenerating properties could be significantly increased by the use of engineered probiotics. Engineering strategies for the development of current systems can be summarized as hydrogel-based systems, nanofiber- or scaffold-based systems, encapsulation systems in the form of microparticles or nanoparticles, bioactive dressing systems, and multifunctional hybrid biointerfaces (Table 2). Of these, the most studied were the hydrogel-based probiotic platforms, as hydrogels offer benefits such as good hydration, adhesion to tissues, protection of the bacteria, and controlled release in the wound, which is a hostile environment.

Several engineering strategies were specifically designed to optimize the redox modulation ability of the probiotics, based on (i) higher scavenging ability of ROS, (ii) longer release of antioxidants, or (iii) enhancement of the antioxidant state in the presence of sustained inflammatory conditions. Several hydrogel-based delivery systems containing *L. rhamnosus* or *L. reuteri* showed strong ROS-scavenging effects against hydroxyl radicals, hydrogen peroxide, and superoxide anions, as well as a strong increase in the antioxidant activity of the antioxidant enzymes and peptides, such as SOD and catalase, and glutathione (GSH) and TAC. For instance, Miao et al. created a pH/ROS dual-responsive sodium alginate hydrogel embedded with *L. rhamnosus*, which exhibited a nearly full scavenging capability for the major ROS species, and significantly enhanced the healing of the infected wound and the tissue regeneration process [125]. In a similar fashion, Liu et al. developed a multifunctional living hydrogel with nano-selenium-producing *L. rhamnosus* and found that this bacterium could significantly decrease the accumulation of ROS, recover the redox balance, stimulate the process of angiogenesis, and also accelerate diabetic wound healing [126]. Other studies used innovative redox-responsive systems, such as hydrogen-producing hydrogels, nitric oxide-generating dressings, metal–phenolic protective coatings, MXene-based bio-heterojunctions, or antioxidant metabolite-loaded biomaterials to further boost the effect of regulating oxidative stress [103,127,128,129,130]. Interestingly, a number of engineered systems have been used to specifically produce ROS or nitrosative stress for antibacterial activity as opposed to just the blockade of oxidative signaling. For example, Gong et al. developed biomimetic ‘phage-like’ microparticles that mimic the probiotics’ ability to induce bactericidal intracellular ROS and NO stress against multidrug-resistant bacteria and promote tissue repair [128]. Similarly, *L. plantarum* systems that generate silver nanoparticles also boosted ROS-induced antibacterial activities without compromising the antioxidant wound-healing activities [131]. These data also provide additional proof for the idea that engineered probiotics are not just ROS suppressors but are also dynamic regulators of redox homeostasis whose actions in vivo can enhance the balance between antimicrobial ROS production and redox signaling for tissue regeneration.

At the signaling pathway level, there was a significant increase in the complexity of the engineered probiotics as opposed to the intrinsic probiotic systems. There have been multiple studies showing modulation of redox-sensitive inflammatory and regenerative pathways such as NF-κB, the pathways of PI3K/Akt/mTOR, MAPK, AMPK, the NLRP3 inflammasome, the pathways of tumor necrosis factor (TNF), the pathways of vascular endothelial growth factor (VEGF) associated with vascularization, and the pathways of transforming growth factor (TGF-β) associated with tissue remodeling [126,129,130,132,133,134,135,136]. One interesting observation is that Liu et al. reported that nanoSe-functionalized *L. rhamnosus* hydrogels concurrently reduced the activation of NF-κB and promoted the activation of the PI3K/Akt/mTOR signaling pathway, which coordinated the antioxidant defense, polarized macrophages, regulated angiogenesis, and activated fibroblasts in diabetic wounds [126]. In a similar manner, Feng et al. showed that 3D-printed hydrogels containing reuterin could regulate multiple interconnected pathways such as: NF-κB, MAPK, PI3K-Akt, TNF, VEGF, and NLRP3 inflammasome signaling pathways, inducing M2 polarization as well as reducing inflammatory injury [133]. Other studies also indicated that NO signaling, endoplasmic reticulum (ER) stress regulation, cytokine–cytokine receptor interactions and transcriptomic remodeling of genes involved in regenerative repair were involved [129,132,135,136]. These engineered systems thus demonstrate more and more programmable, multifunctional signaling properties that can mediate both antioxidative and immunomodulatory, angiogenic, antibacterial, and tissue-regenerative effects—all in one therapeutic system.

A second common therapeutic strategy of engineered probiotic systems was also immune modulation, many of which involved induction of IL-10 and polarization of macrophages. The expression of IL-10 was significantly increased, and pro-inflammatory mediators like TNF-α, IL-1β, IL-6, and iNOS were suppressed with several engineered hydrogels and biomaterial-based delivery systems. For instance, a PEG-based hydrogel encapsulating *L. rhamnosus* was proven to promote the production of IL-10 and the polarization of M2 macrophages, to enhance osteogenic regeneration and angiogenesis in the alveolar wound repair process [132]. Likewise, B. subtilis-embedded metal–phenolic network hydrogels were also shown to boost the amount of IL-10 and TGF-β and to decrease inflammatory infiltration and oxidative stress in infected wounds [103]. Significantly, several studies reported that the delivery systems themselves (and not just the probiotics) played a role by increasing bacterial retention, controlling the release, and protecting the probiotics from the hostile inflammatory environment [103,126,129,130,132,137,138]. From these observations, it can be inferred that the engineering strategies used could be more efficient at improving the antioxidant properties and survival of probiotics, and they could potentially be better at altering the immune–redox interaction in chronic wounds than native probiotics alone.

However, there are a number of key issues and limitations to translation. Firstly, many engineered probiotic systems are highly complex, multifunctional, and include some nanoparticles, responsive polymers, or metal-based materials and synthetic biointerfaces, which can lead to significant issues with the complexity of the manufacturing process, regulatory considerations, scalability, and production costs [121,139,140,141,142,143]. Second, although a number of studies showed the potential for achieving good short-term wound closure results, little research has been conducted on the long-term safety, microbial stability, host–microbiome interactions, and biosafety profile of these systems, especially live engineered probiotics integrated into nanomaterials or responsive systems. Mechanistic interpretation is also difficult, as many multifunctional platforms incorporate antioxidant, antimicrobial, hydrogel, and probiotic bioactivity, and it is challenging to differentiate between the impact of the probiotics and that of other materials or co-delivered therapeutics in the platform. Moreover, the majority of studies have been performed using either acute or artificially created wound models, which only partially mimic the pathological complexity of chronic human wounds with their associated events of persistent ischemia, biofilm formation, metabolic dysfunction, and relapsing inflammation. Critically, too much suppression of ROS can also pose a risk, as it may be able to compromise the physiological antimicrobial mechanism and/or interfere with the beneficial ROS associated with tissue regeneration. Thus, engineered probiotics are a great improvement over native probiotics; however, further studies with more clinically relevant chronic wound models, mechanistic validation, and biosafety evaluation will be necessary to fully realize the translational potential of engineered probiotics as programmable redox therapeutic strategies for wound healing.

**Table 2:** Summary of engineering strategies enhancing probiotic-mediated antioxidant effects in wound healing.

Category	Probiotic (Model)	Engineering Strategy	Redox Modulation	Signaling Pathway	Immune Modulation	Wound Healing	Reference
Hydrogel	*L. rhamnosus* (Mouse wound)	Live probiotic-loaded pH/ROS dual-responsive sodium alginate hydrogel (SA-SPBA@L.rha)	ROS ↓ (·OH, H_2_O_2_, ·O_2_^−^ scavenging ↑ ~80–100%)	NR	Inflammation ↓; inflammatory cell infiltration ↓	↑↑	Miao et al., 2024 [125]
	*L. rhamnosus* (Mouse wound)	Live probiotic encapsulated in multifunctional living hydrogel (PMBV-PVA hydrogel; nanoSe biosynthesized intracellularly + ceramide-coated bacteria)	ROS ↓; H_2_O_2_ ↓; NO ↓; nanoSe-mediated ROS scavenging	NF-κB ↓ (p-NF-κB, p65 ↓); PI3K/Akt/mTOR ↑	M2 polarization ↑; M1 markers ↓; IL-6 ↓; TNF-α ↓; IL-1β↓; inflammation ↓	↑↑	Liu et al., 2025 [126]
	*L. rhamnosus* (Rat wound)	Live probiotic encapsulated in injectable photo-crosslinkable PEG hydrogel with calcium phosphate nanoparticles (CP-LGG@Gel; in situ light-activated)	NR	TNF signaling pathway involvement (transcriptomics); cytokine–cytokine receptor interaction ↑	M2 polarization ↑; IL-10 ↑; TNF-α ↓; inflammation↓	↑↑	Guo et al., 2026 [132]
	*L. reuteri* (Mouse wound)	Metal-phenolic self-assembly shielded live probiotic (L. reuteri@FeTA) in hydrogel (Gel/L@FeTA)	NR	Angiogenesis-related proteins ↑ (VEGF ↑; CD31 ↑)	TNF-α ↓; IL-1β ↓; IL-10 ↑; TGF-β ↑ (anti-inflammatory ↑)	↑↑	Zhou et al., 2023 [129]
	*L. reuteri* (Mouse wound)	Viable probiotic encapsulated in calcium alginate hydrogel (LR@CAH; with acid-responsive hydrogen-producing material)	ROS ↓; oxidative stress ↓ (via glucose consumption + hydrogen production); antioxidant enzyme activity ↑	NR	Inflammation ↓	↑↑	Wang et al., 2025 [127]
	*L. reuteri* (Rat wound)	Reuterin incorporated into 3D-printed double-layer hydrogel (ACG-GC/Reu/SPS-PDMS)	NR	NF-κB ↓; AMPK ↓; IL-17 ↓; NLRP3 inflammasome activation; MAPK, TGF-β, PI3K-Akt, TNF, VEGF pathways ↑	IL-10 ↑; TGF-β ↑; Arg-1↑; M2 polarization ↑; TNF-α ↓; IL-1β ↓; iNOS ↓; inflammation ↓	↑↑	Feng et al., 2025 [133]
	*L. reuteri* (Rat wound)	Reuterin-crosslinked chitosan hydrogel (injectable, pH-responsive release system)	ROS ↓; DPPH scavenging ↑ (>60%); oxidative stress–induced cell damage ↓; cell viability under H_2_O_2_ ↑	NR	Inflammation/infiltration ↓; TGF-β1 ↓	↑↑	Gao et al., 2025 [144]
	*L. casei* (Rat wound)	Probiotic extracts + NO donor = biomimetic phage-like microparticles, further encapsulated in GO-doped hydrogel	NO release ↑; nitrosative stress ↑; intracellular ROS ↑ (in bacteria); oxidative stress ↑	NR	Inflammation/infiltration ↓; bacterial toxicity ↓	↑↑	Gong et al., 2022 [128]
	*B. subtilis* (Rat wound)	Living probiotic encapsulated in metal–phenolic networks (MPNs) and loaded into KGM/XG hydrogel (KGXM-PCB@Bsubtilis)	ROS ↓ (intracellular ROS scavenging ↑); antioxidant activity ↑ (DPPH/ABTS scavenging ↑)	NR	IL-10 ↑; TGF-β ↑; IL-6 ↓; iNOS ↓; inflammation ↓	↑↑	Wu et al., 2025 [103]
	*P. pentosaceus* (Rat wound)	Live probiotic incorporated in hydrogel (C-phycocyanin-loaded whey protein nanofibril hydrogel)	MDA ↓ (54.76–98.58%); GSH ↑; TAC ↑ (antioxidant capacity ↑)	COX-2 inhibition ↑ (phycocyanin-related)	Inflammation/infiltration ↓; microbiome balance ↑; immune response modulation ↑	↑↑	El-Dein et al., 2026 [134]
Nanofiber	*L. rhamnosus* (Antioxidant model)	Probiotic exopolysaccharide (EPS-P14)-based electrospun nanofibrous membrane (EPS/PEO ± Cu/Ag-doped bioactive glass); no live bacteria	ROS scavenging ↑ (DPPH ~65–70%; FRAP reducing power ↑)	NR	NR	↑↑	Akhtach et al., 2025 [145]
	*L. casei* (Mouse wound)	Probiotic loaded silk fibroin/alginate scaffold	NR	Endoplasmic reticulum stress ↓	M2 polarization ↑; inflammation ↓	↑↑	Dou et al., 2023 [135]
Microparticle	*L. plantarum* (scratch wound)	Cell-free supernatant-mediated biosynthesis of silver nanoparticles (AgNPs)	ROS ↑ (AgNP-induced oxidative stress for antibacterial effect); antioxidant activity ↑ (DPPH scavenging 9.9–52.4%)	NR	NR	↑↑	Vijayakumar et al., 2023 [131]
	*L. plantarum* (Mouse wound)	Viable probiotic incorporated in sponge dressing + nanocurcumin (CSLNs; solid lipid nanoparticles)	LPO ↓; Catalase ↑; GSH ↑ (antioxidant enzymes ↑)	NR	TNF-α ↓; MMP-9 ↓ (inflammation ↓); VEGF ↑; TGF-β ↑	↑↑	Sandhu et al., 2023 [137]
	*L. plantarum* (Rat wound)	Live probiotic encapsulated in lyophilized polymeric microparticles (chitosan–alginate) + prebiotic (FOS)	ROS-related oxidative stress ↓; FRAP (TAC) ↑; MDA (lipid peroxidation) ↓	NR	Inflammation/infiltration ↓	↑↑	Farahani et al., 2023 [146]
	*L. rhamnosus* (Rat wound)	Live probiotic loaded in Dextran-Gelatin-Gellan Gum composite microspheres (DGGcm) biomaterial (sustained release system)	NR	NR	IL-6 ↓; IL-10 ↑; inflammation ↓	↑↑	Diao et al., 2025 [138]
	*L. rhamnosus* (Antioxidant model)	Live probiotic coated with γ-Fe_2_O_3_ nanoparticles (nanobiohybrid; γ-Fe_2_O_3_ NPs–LR) via electrostatic self-assembly	ROS ↑ (from γ-Fe_2_O_3_ NPs antibacterial mechanism); antioxidant activity ↑ (DPPH scavenging ↑); SOD/CAT ↑ (implied from *L. rhamnosus* secretion); oxidative stress scavenging ↑	NR	NR	NR	Shingade et al., 2026 [147]
Dressing	*L. fermentum* (Rat wound)	Live probiotic embedded in patch (lyophilized bacteria in alginate microbeads within gas-permeable dressing producing NO)	NR	NO signaling ↑ (nitric oxide-mediated wound healing process)	Inflammation/infiltration ↓	↑↑	Jones et al., 2012 [136]
Other	*L. rhamnosus* (Mouse wound)	Live probiotic engineered bio-heterojunction (P-bioHJ): LG conjugated with MXene (Ti_3_C_2_) quantum dots + FeS + LOx; NIR-triggered system	ROS scavenging ↑; intracellular ROS ↓; SOD ↑; CAT ↑; MDA ↓; hydroxyl radical clearance ↑	NF-κB ↓; IκBα ↓; TNF-α ↓; TNF signaling modulation (RNA-seq); anti-inflammatory pathway inhibition	Inflammation ↓; TNF-α ↓; IL-6 ↓; anti-inflammatory effect ↑	↑↑	Qin et al., 2024 [130]

↑: increase; ↑↑: marked increase; ↓: decrease; NR: not reported. *B. subtilis*: *Bacillus subtilis*; *L. casei*: *Lactobacillus casei* or *Lacticaseibacillus casei*; *L. fermentum*: *Lactobacillus fermentum*; *L. plantarum*: *Lactobacillus plantarum* or *Lactiplantibacillus plantarum*; *L. reuteri*: *Lactobacillus reuteri* or *Limosilactobacillus reuteri*; *L. rhamnosus*: *Lactobacillus rhamnosus* or *Lacticaseibacillus rhamnosus*; *P. pentosaceus*: *Pediococcus pentosaceus*. ABTS: 2,2′-azino-bis(3-ethylbenzothiazoline-6-sulfonic acid; ACG: adenylyl cyclase G; AMPK: AMP-activated protein kinase; AgNPs: silver nanoparticles; CAT: catalase; COX-2: cyclooxygenase-2; DGGcm: Dextran-Gelatin-Gellan Gum composite microspheres; DPPH: 2,2-Diphenyl-1-picrylhydrazyl; EPS: exopolysaccharide; FOS: fructooligosaccharides; FRAP: ferric reducing antioxidant power; GSH: glutathione; HO-1: Heme Oxygenase-1; HUVEC: human umbilical vein endothelial cells; KGM: konjac glucomannan; LPO: lipid peroxidation; M2: macrophage M2; MAMPs: microbe-associated molecular patterns; MDA: malondialdehyde; MPNs: myeloproliferative neoplasms; MSC: mesenchymal stem cell; MT-1: Metallothionein-1; nanoSe: nano-selenium; NIR: near-infrared; PEO: polyethylene oxide; PEG: polyethylene glycol; PMBV: phospholipid polymer; PVA: polyvinyl alcohol; Reuterin: *L. reuteri* -derived metabolite; ROS: reactive oxygen species; SA: sodium alginate; SOD: superoxide dismutase; SPBA: succinic acid-4-(bromomethyl)phenylboronic acid; SPS: solubility parameters; TAC: antioxidant capacity; TNF: tumor necrosis factor; VEGF: vascular endothelial growth factor. Paraprobiotics (Microbe-Associated Molecular Patterns, MAMPs): non-viable microbial components (e.g., heat-killed bacteria, lysates, or purified cell components) that retain biological activity and exert effects by interacting with host immune receptors. Detailed information for Table 2 is provided in Supplementary Table S2.

### 3.4. Clinical Evidence of Probiotic Antioxidant Effects in Wound Repair

Thus, there is limited information (one randomized controlled clinical study in humans) available showing that this efficacy could be linked to an antioxidant effect of probiotics during wound healing (Table 3) [148]. Mohseni et al. in their clinical double-blind, placebo-controlled study, investigated the effects of a multi-species oral probiotic formulation consisting of *Lactobacillus acidophilus*, *L. casei*, *L. fermentum*, and *Bifidobacterium bifidum* on diabetic foot ulcers in 60 patients during a 12-week intervention. Of particular importance is the fact that oral live probiotic supplementation may be associated with wound-healing outcomes and systemic oxidative stress parameters in humans. This study, however, tested live probiotics and should thus not be interpreted as clinical evidence for probiotic-EV therapy but as indirect evidence for the concept of redox modulation via probiotics. Probiotics were shown to have a significant effect on decreasing the length, width, and depth of the ulcer, with a simultaneous beneficial effect on glycemic control and the inflammatory status of patients compared with the placebo group. Interestingly, some biomarkers related to antioxidants significantly changed after probiotic supplementation; for instance, at the end of the supplementation, the concentration of malondialdehyde (MDA) and high-sensitivity C-reactive protein (hs-CRP) had decreased, and NO and TAC had increased. In particular, there was a significant reduction in plasma MDA levels (–0.8 ± 0.8 μmol/L) and a significant increase in TAC (+179.3 ± 97.2 mmol/L) in the probiotic group, consistent with reduced systemic oxidative stress and changes in oxidant/antioxidant balance. These antioxidant changes were accompanied by a decrease in fasting plasma glucose, insulin resistance, and HbA1c, which further indicated that the modulation of metabolic dysfunction and oxidative stress in diabetic wound repair may be mechanistically linked.

Mechanistically, the authors suggested that the probiotics’ beneficial effects might be mediated through several pathways, such as inhibiting the production of inflammatory cytokines, increasing the activity of endogenous antioxidant enzymes, modifying the inflammatory responses of gut microbiota-derived SCFA signaling and decreasing ROS-driven lipid peroxidation [148]. Interestingly, while there was no significant change in the levels of GSH, the simultaneous decrease in MDA and increase in TAC and NO suggest that probiotics might favor the general regulation of redox balance in the organism rather than any single antioxidant pathway. The observed rise in NO is also remarkable, as NO is involved in a variety of wound healing processes, such as the regulation of angiogenesis, vasodilation, antimicrobial defense, and tissue remodeling. Thus, these findings are consistent with the possibility that probiotic administration may influence systemic redox homeostasis rather than acting through suppression of a single oxidative pathway.

Although this study provides preliminary clinical support for probiotic-mediated redox modulation, there are a number of limitations that significantly restrict its interpretation and generalizability [148]. Firstly, the intervention was multimodal (oral multispecies probiotics, standardized antibiotic therapy, and wound care), and thus it was hard to differentiate the effect of the probiotics from systemic metabolic improvements or antimicrobial therapy. Second, this study focused on biomarkers of systemic oxidative stress but not local wound-specific redox dynamics, distribution of ROS in the wound tissue, macrophage phenotype, or signaling pathways in the microenvironment of the wound. Thus, it is not known if the antioxidant properties had a direct effect on the healing of the wounds or if their effect was actually secondary to improved metabolism. Moreover, while there was a significant decrease in the size of the ulcers, the study period was relatively limited, with no extensive evaluation of long-term recurrence, scar quality, persistence of the biofilm, or chronic inflammatory relapse. No validation experiments or pathway-specific investigations were performed, and no microbiome sequencing, bacterial colonization analysis, or cytokine profiling (outside of hs-CRP) was conducted to aid mechanistic characterization. The authors themselves noted that the study had several outstanding challenges, such as the failure to analyze the fecal microbiota and to perform direct insulin sensitivity testing, and host–microbiome interaction analysis was not well characterized. Further, the probiotic formulation was made up of several bacterial strains, making it impossible to assess the individual contribution of the different strains to the antioxidant activities. Importantly, the antioxidant properties were measured systemically in diabetic patients with significant metabolic disorders, and it is possible that the antioxidant properties were not comparable to those in other types of wounds or other patient groups. More importantly, in the context of the present review, the intervention consisted of orally administered live probiotics and did not involve isolated or engineered probiotic-derived EVs. Therefore, the study cannot establish the efficacy, safety, biodistribution, or wound-specific redox effects of probiotic-derived EV therapy.

Despite these caveats, this study provides limited but relevant clinical support for the broader concept that live probiotic administration may be associated with systemic redox modulation and wound-healing outcomes. It should not, however, be interpreted as direct translational evidence for probiotic-derived EV therapy. To date, there are no direct human clinical trials demonstrating the efficacy or safety of probiotic-derived EVs for wound healing. Overall, the results from these markers in oxidative stress, inflammatory status, metabolic control, and wound closure demonstrated that the modulation of systemic redox homeostasis by the administration of probiotics could have clinically important implications regarding chronic wounds. However, so few controlled human studies have been initiated, which shows a huge gap in the realm of study. Thus, larger multicenter cohorts with standardized formulations of probiotics, direct analysis of local oxidative signaling pathways, mechanistic analysis of wound-specific events, evaluation of safety in long-term studies, characterization of the probiotic transcriptome, and evaluation of the microbiome will be required for future clinical investigations to further elucidate the therapeutic role of probiotics as a redox-regulatory intervention in human wound healing.

**Table 3:** Clinical evidence of probiotic-mediated antioxidant modulation in wound healing.

Probiotic	Intervention	Design	Population	Biomarker (Antioxidant/Oxidative Stress)	Clinical Outcome	Reference
*L. acidophilus*, *L. casei*, *L. fermentum*, *B. bifidum* (multi-strain)	Oral live probiotics (2 × 10^9^ CFU/g each, oral capsule)	Randomized, double-blind, placebo-controlled trial	Patients with diabetic foot ulcer, *n* = 60 (30 vs. 30), age 40–85, duration: 12 weeks	MDA ↓; TAC ↑; NO ↑; GSH ↔; hs-CRP ↓	Ulcer length, width, depth ↓; wound healing rate ↑; improved glycemic control (FPG, HbA1c ↓); cholesterol ↓; inflammation ↓	Mohseni, 2018 (IRCT201603085623N68) [148]

↑: increase; ↓: decrease; ↔: no significant change. *L. acidophilus*: *Lactobacillus acidophilus*; *L. casei*: *Lactobacillus casei*; *L. Fermentum*: *Lactobacillus Fermentum*; *B. bifidum*: *Bifidobacterium bifidum*. MDA: malondialdehyde; TAC: antioxidant capacity; NO: nitric oxide; hs—CRP: high—sensitivity C—reactive protein; GSH: glutathione; FPG: fasting plasma glucose; HbA1c: haemoglobin A1c. Note: This clinical study evaluated orally administered live probiotics rather than probiotic-derived EVs; therefore, it provides indirect clinical evidence for probiotic-mediated redox modulation and should not be interpreted as clinical evidence for probiotic-EV therapy.

**Table 4:** Probiotic-derived extracellular vesicles targeting antioxidant effects in wound healing.

Strain/EV Source	EV Preparation & Characterization	EV Properties/Cargo	Treatment	Experimental Model	Redox/Immune Findings	Proposed Pathway	Mechanistic Validation	Wound/Tissue Outcome	Reference
*L. reuteri* DSM 20016; MVs	Cell-free culture supernatant; centrifugation (5000× *g*, 20 min) + 0.45-μm filtration + ultracentrifugation (129,000× *g*, 1.5 h; repeated at 129,000× *g*, 16 h); TEM, SEM, DLS	Mean size ≈60 nm (DLS); spherical membrane vesicles; 3-HPA identified by LC–MS; vesicle concentration and purity assessment NR; endotoxin/bacterial component assessment NR	Natural MVs; oral mucosal wound: topical MVs (10 μL, 200 μg/mL, twice daily); cutaneous wound: MV-loaded hydrogel, 200 μg MVs on days 0 and 7	In vitro: RAW 264.7 macrophages (LPS-induced inflammation); In vivo: mouse oral mucosal ulcer model + mouse skin wound model	Redox: ROS ↓; mitochondrial ROS ↓; MDA ↓; NO ↓; mitochondrial membrane potential stabilization ↑; CI activity ↑/CII normalization; oxidative stress ↓ Immune: IL-10 ↑; TNF-α ↓; IL-1β ↓; IL-6 ↓; M2 macrophage polarization ↑ (CD206↑, Arg-1↑; iNOS↓); inflammation ↓	3-HPA-associated inhibition of mitochondrial permeability/mPTP opening → reduced oxidative stress → anti-inflammatory macrophage phenotype	Yes—cargo identification and pharmacological pathway interrogation: LC–MS identified 3-HPA; isolated 3-HPA reproduced redox/immunomodulatory effects; cyclosporin A/CypD-associated mPTP inhibition used to interrogate mitochondrial permeability mechanism; direct molecular target of 3-HPA not established	Wound closure ↑; re-epithelialization ↑; inflammatory infiltration ↓; collagen organization ↑; hair follicle formation ↑; tissue regeneration ↑; mucosal healing ↑	Chen et al., 2024 [92]
*L. reuteri*; MVs	Gradient differential ultracentrifugation; morphology characterization; NTA; transcriptomic analysis of MV-treated cells	Mean size ~120 nm (NTA); vesicle concentration NR; molecular cargo profiling NR; purity assessment NR; endotoxin/bacterial component assessment NR	Natural MVs, biomaterial-engineered delivery; MVs covalently anchored to carboxylated chitosan and incorporated into oxidized hyaluronic acid hydrogel (Gel-MVs) for controlled local release; dose NR	In vitro: cell prolifera-tion/migration assays; In vivo: mouse skin wound model	Redox: Direct redox outcomes NR Immune: Inflammation ↓	Transcriptome-associated regulation of angiogenesis/metabolic pathways; VEGF/CD31-associated vascularization↑	Pathway-associated/transcriptomic evidence only; no inhibitor, knockdown, cargo-depletion, or rescue validation reported	Angiogenesis ↑; vasculariza-tion ↑; wound closure ↑; cell proliferation ↑; cell migration ↑; collagen deposition ↑	Zhou et al., 2025 [95] *
*L. reuteri*; MVs †	Differential centrifugation + ultracentrifugation (100,000× *g*); Cryo-TEM; NTA; UPLC-ESI-MS/MS metabolomic profiling	Mean size ~145 nm; concentration NR; metabolomic profiling identified major bioactive constituents, including abundant phospholipids; purity assessment NR; endotoxin/bacterial component assessment NR	Natural MVs; 20 μg/mL in vitro; ROS-responsive DNA hydrogel (MVs-Gel) for controlled local release ± NIR photothermal therapy; in vivo MV dose NR	Mouse diabetic infected skin wound model	Redox: ROS ↓; antioxidant activity ↑; apoptosis ↓ (Bax ↓/Bcl-2 ↑); Nrf2/HO-1 ↑ Immune: IL-10 ↑; TGF-β ↑; TNF-α ↓; IL-6 ↓; M2 macrophage polarization ↑(CD206 ↑; CD86 ↓); inflammation ↓	Nrf2/HO-1 -mediated redox regulation ↑; NF-κB -mediated inflammation↓; PI3K/Akt ↑; AMPK ↑	Pathway-associated validation by Western blot and transcriptomics/GSEA; no inhibitor, knockdown, cargo-depletion, or rescue validation reported	Wound clo-sure ↑; re-epithelialization ↑; keratinocyte migration ↑; angiogenesis ↑(CD31 ↑); neovasculari-zation ↑; col-lagen deposi-tion ↑; α-SMA ↑; antibacterial activity ↑	Tai et al., 2026 [96]
*L. rhamnosus* GG (ATCC 53103); LGG-MVs	Differential centrifugation + 0.22-μm filtration + ultracentrifugation (150,000× *g*, 2 h, twice) with ultrafiltration; TEM; NTA; zeta-potential analysis; miRNA sequencing	Size 30–200 nm, main peak ~180 nm; zeta potential ~−19.4 mV; concentration estimated by BCA protein assay; miRNA profiling identified 494 miRNAs, with miR-21-5p highly enriched; purity assessment not formally reported; endotoxin/bacterial component assessment NR	Natural LGG-EVs; in vitro 50 ng/μL; in vivo 50 μg EVs in 100 μL PBS by daily subcutaneous injection around wounds	In vitro: scratch wound (HaCaT keratinocytes; HUVEC en-dothelial cells); in vivo: mouse skin wound model	Redox: Direct redox outcomes NR Immune: Direct imunemodulation endpoints NR	miR-21-5p-mediated PI3K/AKT–HIF1α signaling ↑; VEGF-associated angiogenic signaling↑	Yes—miRNA cargo profiling + miR-21-5p inhibitor functional validation; inhibition attenuated LGG-EV-induced proliferation/migration and abolished p-AKT/HIF1α activation; no rescue experiment reported	Wound clo-sure ↑; re-epithelialization ↑; keratinocyte proliferation ↑; endothelial cell prolifera-tion/migration ↑; angiogene-sis ↑; granula-tion tissue ↑; collagen dep-osition ↑	Wang et al., 2024 [94]
*L. casei* DSM 20011; *L. plantarum* NCIMB 8826; MVs	Centrifugation + 0.45-μm filtration + ultracentrifugation (100,000× *g*) + SEC; cryo-TEM; NTA; LC–MS/MS proteomics; SEM of BPs	~50–150 nm; NTA-based particle concentration reported; culture-dependent protein cargo; thioredoxin identified in *L. casei* pH 6.5 MVs; SEC-purified; endotoxin/bacterial component assessment NR	Natural MVs; direct MV-loaded or bacteriomimetic microparticle-loaded HEC hydrogel; topical application; in vivo dose NR	In vitro: Ha-CaT keratino-cyte scratch wound; In vivo: mouse tail wound model	Redox: Direct redox outcomes NR Immune: TNF ↓; IL-10 modulation; IL-10/TNF ratio ↑; an-ti-inflammatory effect ↑; neutrophil infiltration ↓; M2-like ef-fect↑	No specific pathway established; proteomic composition associated with culture-dependent immunomodulation	Proteomic association only; no inhibitor, knockdown, cargo-depletion, or rescue validation	BP hydrogel: early wound-width reduction/re-epithelialization ↑; dermal thickness ↓; revascularization ↑; overall wound-closure time ↔; collagen deposition ↔	Kuhn et al., 2024 [93]

↑: increase; ↓: decrease; ↔: no significant change; NR: not reported. AMPK: AMP-activated protein kinase; α-SMA: alpha-smooth muscle actin; BAX: BCL-2-associated X protein; HaCaT: human skin keratinocytes; HUVEC: human umbilical vein endothelial cell line; MDA: malondialdehyde; mPTP: mitochondrial permeability transition pore; MVs: membrane vesicles; NIR: near-infrared irradiation; ROS: reactive oxygen species; VEGF: vascular endothelial growth factor; DLS: dynamic light scattering; SEM: scanning electron microscopy; TEM: transmission electron microscopy; UPLC-ESIMS/MS: ultra-performance liquid chromatography electrospray ionization mass spectrometry; HEC: hydroxyethyl cellulose. Note: * For the reference [95], the full-text article was not available for assessment; data extraction was therefore based on the abstract and available Supplementary Materials. Methodological details not identified in these sources are reported as NR and should be interpreted accordingly. Note: † The original study [96] referred to *L. reuteri*-derived vesicles as “outer membrane vesicles (OMVs)”; however, because *L. reuteri* is a Gram-positive bacterium and lacks an outer membrane, these vesicles are referred to here as membrane vesicles (MVs) for terminological consistency.

## 4. Extracellular Vesicles: A Redox-Modulating Delivery System

### 4.1. Biology of Extracellular Vesicles

EVs are vesicles that are enclosed by membranes and are released by almost all types of cells and are now reported to play a key role in intercellular communication in physiological and pathological conditions [26,28,29,149,150]. In mammalian systems, according to their biogenesis and size distribution, EVs can be divided into several large ones, such as exosomes, microvesicles, and apoptotic bodies [149,151,152]. The smallest population of EVs, around 30–150 nm in diameter, is exosomes that are present in the endosomal system and are formed by invagination of multivesicular bodies (MVBs) before being secreted from cells through fusion with the plasma membrane. Microvesicles have a size of 100–1000 nm and are formed by direct outward budding and shedding from the plasma membrane, while apoptotic bodies are formed during programmed cell death and are larger vesicles (>1 μm). However, significant overlap is observed regarding the size, composition, and biological functions of these various types of EVs in operation, and in recent years, there have been growing calls to use the more general term ‘extracellular vesicles’ where there is a high degree of uncertainty about the origin of the biogenic vesicles. In order to keep the usage of terminology consistent throughout this review, the term extracellular vesicles (EVs) is used to refer to membrane-enclosed particles released by cells. In bacterial systems, the term bacterial extracellular vesicles (bEVs) is used as the broad term for vesicles released by bacteria, whereas the term probiotic-derived extracellular vesicles (probiotic-EVs) refers specifically to bEVs derived from probiotic or beneficial bacterial strains discussed in this review. The term outer membrane vesicles (OMVs) is reserved for vesicles originating from the outer membrane of Gram-negative bacteria. For Gram-positive bacteria, which lack an outer membrane, the term membrane vesicles (MVs) is used, particularly when this terminology was adopted in the original study. Probiotic-EVs are used as the general therapeutic term, and study-specific terminology is kept when necessary to synthesize evidence from studies. Exosomes, microvesicles, and apoptotic bodies are mostly eukaryotic subclasses of EVs and are not synonymous with bacterial vesicles. Among the probiotic and commensal bacteria discussed in this review, *Limosilactobacillus reuteri*, *Lactiplantibacillus plantarum*, *Lacticaseibacillus rhamnosus* GG, *Lacticaseibacillus casei*, *Bifidobacterium spp*., and *Bacillus spp*. are Gram-positive bacteria and therefore do not possess an outer membrane; their vesicles are referred to as MVs, bEVs, or probiotic-EVs as appropriate. Conversely, Gram-negative bacteria such as *Akkermansia muciniphila*, *Escherichia coli Nissle* 1917, *Bacteroides fragilis*, *Bacteroides thetaiotaomicron,* and *Parabacteroides* spp. might well be called OMVs if the vesicles are indeed originating from the outer membrane.

Significantly, EVs are not just “cellular debris” or “waste products” but are biologically active nanostructures that can carry a variety of molecular loads such as proteins, lipids, nucleic acids, metabolites, cytokines, signaling molecules, etc., to target cells. During this cargo transfer, EVs are involved in various mechanisms associated with inflammation, immune regulation, angiogenesis, tissue remodeling, and adaptation to cellular stress [153,154]. EV molecular composition is mostly dependent on the molecular composition of the EVs’ parental cells, and on the physiological state of the cells. Common mammalian EV markers are proteins such as tetraspanins (CD9, CD63, CD81), endosomal sorting proteins (TSG101 and Alix), heat shock proteins (HSP70), and different membrane-associated lipids and adhesion molecules [154,155]. In particular, conditions of environmental stress, such as inflammation, hypoxia, and oxidative stress, can profoundly affect the process of EV biogenesis, secretion, and the cargo carried, which will affect their biological activity downstream [156,157,158].

Many studies in the last few years have been investigating EVs for therapeutic applications, which are based on their intrinsic biocompatibility, their nanosize, and capacity to transport cargo between cells. EVs have several unique characteristics that suggest they may be more biologically stable than traditional soluble biologics or free therapeutic molecules, and they are inherently less immunogenic, can be taken up easily by cells, and may carry natural targeting molecules to tissues on the surface of the vesicles that can interact with receptors on cell surfaces [159,160]. As well, the EVs’ lipid bilayer membrane acts as a barrier against the enzymatic degradation of encapsulated cargo and allows for delivery through complicated biological barriers [161,162]. These properties have drawn the attention of many scientists to pursue EV-based therapeutics in many different disorders relating to oxidative stress, such as chronic wounds, neurodegenerative disorders, cardiovascular injuries, and inflammatory disorders. While most current EV research has investigated vesicles derived from mammalian cells (especially MSC-derived EVs), an increasing number of studies have demonstrated the potential of microbial-derived EVs to also contain important immunomodulatory and regenerative properties, extending the therapeutic scope of EV-based regenerative medicine [27].

### 4.2. EV-Mediated Redox Regulation Through Antioxidant Enzymes and microRNA Cargo

Much of the current evidence for EV-mediated redox regulation has been derived from mammalian cell-derived EVs. In these systems, EVs have been increasingly recognized as potential carriers of antioxidant enzymes, redox-sensitive proteins, and regulatory non-coding RNAs [163,164,165]. Increasingly, there are some pieces of evidence that EVs from different cell types (MSCs, immune cells, endothelial cells, and stressed epithelial cells) possess functional antioxidant machinery, which can directly modulate oxidative signaling within the recipient cells or tissues [163,166,167]. The antioxidant enzymes under most investigation regarding their association with EVs are SOD, catalase, GPx, thioredoxin and peroxiredoxins, which are involved in the detoxification of ROS and in the maintenance of the intracellular redox equilibrium [163,165,168]. Importantly, these enzymes could keep their catalytic activity after transfer by EVs and therefore they may quickly strengthen the recipient cells’ own antioxidant defense of the recipient cells in case of oxidative stress. Recent research also showed that EVs secreted upon inflammatory or oxidative stress have a selective enrichment of antioxidant enzymes and stress-response mediators, suggesting that EV excretion might be an adaptive mechanism to communicate intercellular redox signals to other cells [156,169,170,171,172]. For instance, it was shown that EV-associated catalase and SOD can decrease intracellular ROS generation, maintain mitochondrial integrity, suppress lipid peroxidation, and reduce apoptosis in various models of oxidative stress-associated diseases [163,173]. Together, these observations contribute to a new idea that EVs could act as endogenously derived antioxidant nanocarriers that could be used to translocate redox-protecting machinery to damaged tissues.

Besides the protein-based antioxidant system, EVs can exert their antioxidant activity by transferring miRNAs, which are able to regulate the oxidative-related pathways at the post-transcriptional level [174,175,176,177]. Some redox-sensitive functions, like mitochondrial function, inflammatory signaling, macrophage polarization, angiogenesis, apoptosis, and activation of antioxidant transcriptional programs, can be modified by EV-associated miRNAs [174,178,179]. In mammalian EV systems, miR-21 and miR-146a are two redox-regulatory miRNAs that are well characterized and often found in EV cargo during times of inflammation or oxidative stress [180,181,182,183,184,185,186]. miR-21 is known to inhibit apoptosis, promote cell survival signals, and modulate PTEN/PI3K/Akt-mediated antioxidant response, while miR-146a is a well-known negative regulator of NF-κB-dependent inflammatory signaling and ROS amplification [187,188]. Simultaneously, other EV-carried miRNAs such as miR-155, miR-210, and miR-223 have been shown to play a role in the regulation of hypoxia adaptation, macrophage polarization, mitochondrial metabolism, and Nrf2-mediated antioxidant pathways [189,190,191,192,193,194]. Interestingly, oxidative stress alone is involved in the selective loading of EVs with miRNAs, through redox-sensitive cargo-sorting mechanisms of RNA-binding proteins and signaling pathways related to stress [174]. Thus, EV-mediated miRNA transfer can be considered not just passive molecular transfer but as a dynamic epigenetic communication system that can influence the redox signal networks of the recipient cells and tissues.

All these observations indicate that EVs contribute to redox regulation in at least two different ways: directly by carrying out detoxification of ROS in the recipient cell thanks to endosomal antioxidant proteins they have transferred and indirectly by carrying out regulation of oxidative signaling through transcriptional or epigenetic regulation mediated by their miRNA cargo [163,165,171,174]. This redox multifunctional regulation can be especially important in chronic wound conditions where oxidative stress, mitochondrial dysfunction, inflammatory intensification, and cellular adaptation in the wound environment provide a complex mix of factors that ultimately lead to failure to heal. Because of their role in regulating antioxidant defense systems, inflammatory signaling pathways and cell-regenerative responses all at once, the use of EVs as the next generation of acellular therapeutics for oxidative stress-associated tissue injury has sparked interest. It is worth noting that the majority of the current evidence is from mammalian cell-derived EVs; however, there is now a growing interest in studying EVs derived from microbes and probiotics that could have specific redox-modulating and immunoregulatory functions and thus play a role in wound healing and the microbiome in wound regeneration.

### 4.3. Therapeutic Advantages of EVs over Conventional Antioxidant Delivery Systems

A primary problem of the traditional antioxidant therapeutic approach to chronic wound treatment is that free antioxidant molecules cannot provide sustained, localized and biologically effective redox modulation in the hostile chronic wound environment [17,23,195]. While some small-molecule antioxidants and soluble antioxidant enzymes have shown great promise in vitro, most of these have proven difficult in clinical applications due to a short half-life, limited tissue uptake, non-specific tissue distribution, poor cellular uptake, and rapid degradation [20,196]. In chronic wounds with chronic inflammation, high protease production, presence of biofilm, hypoxia, and oxidative stress, many free antioxidants are quickly inactivated before reaching amounts in the cells to have any therapeutic effect. Moreover, indiscriminate systemic ROS scavenging could have unwanted side effects too, as it could interfere with antimicrobial defense, angiogenesis, and tissue regeneration, which are important physiological processes that rely on ROS for their regulation. Such restrictions have been spurring an increased interest in EV-based delivery systems as biologically adaptive nanocarriers to overcome many pharmacokinetic and mechanistic challenges faced by conventional antioxidant therapies.

However, EVs have several special structural and functional features that significantly increase the efficiency of therapeutic delivery in an oxidative tissue environment, compared to soluble antioxidants. Firstly, EVs offer intrinsic protection from degradation and inactivation by enzymes and clearance from circulation, allowing the stability and bioactivity of antioxidant proteins, nucleic acids, and signaling molecules to be maintained throughout the journey of EVs in the extracellular space [154,197]. This protective architecture can be especially important for chronic wounds, as increased levels of proteases and sustained inflammatory stress are often present in the wound environment and can cause instability of free therapeutic agents. Moreover, EVs are of the correct size for tissues—nanosized with very flexible membranes to penetrate tissues and diffuse across complex extracellular matrices [198,199]. It has been shown in several studies that EVs are capable of crossing biological barriers and can be internalized into cells efficiently by different internalization methods, including receptor-mediated uptake, endocytosis, phagocytosis, and macropinocytosis [197,200]. Thus, EVs are able to directly transport redox-regulatory molecules to the recipient cells, such as fibroblasts, keratinocytes, endothelial cells, macrophages, and stem/progenitor cells, which are all involved in wound repair. This intracellular delivery feature sets EVs apart from many of the traditional antioxidants, which have their main action outside the cell, with little penetration into the oxidative compartments within cells, such as the mitochondria.

The natural targeting and intercellular communication properties of EVs also have the promise to increase therapeutic specificity and decrease the occurrence of off-target effects [159,201]. Surface-associated adhesion molecules such as integrins, tetraspanins, glycans, and receptor-binding proteins may partially determine the tissue tropism under inflammatory or injured conditions by playing a role in selective EV uptake by recipient cells. Importantly, the biodistribution and biological activity of EVs are also influenced by the physiological state of the parental cells, and stress-adapted EVs are able to selectively target damaged tissue for delivery of regenerative and redox-regulatory signals [202,203]. Hence, EV-mediated delivery may provide a biologically organized mode of intercellular cargo transfer compared with free soluble antioxidants. Furthermore, EVs can even convey several classes of bioactive molecules (such as antioxidant enzymes, anti-inflammatory cytokines, lipids, metabolites and mRNAs, and regulatory miRNAs), which can all be achieved in a single platform at the nanoscale, thus enabling the coordinated modulation of several processes such as oxidative stress, inflammation, angiogenesis, and tissue remodeling. This polyfunctional therapeutic activity is particularly relevant in chronic wounds, the failure of which is not due only to oxidative damage; therefore, the interaction between redox, immune, microbial, and regenerative signaling must be considered.

For many mammalian cell-derived EV platforms, relatively low immunogenicity compared with live-cell transplantation has been proposed as a potential therapeutic advantage [26,204]. This assessment, however, cannot be directly transferred to EVs from bacteria, which might include microbial elements that stimulate the immune system and thus warrant a biosafety assessment. Another benefit of EVs is that they do not have some of the practical drawbacks of stem cell therapy, such as poor engraftment, short survival time post-transplant, and potential uncontrolled differentiation or long-term genetic instability. Another advantage of their acellular nature is the possibility of incorporation of these materials into biomaterials including hydrogels, nanofibers, wound dressings, and injectable scaffolds, which are easily scalable [10,205,206,207,208,209]. All these features render EVs extremely promising next-generation redox-regulatory therapeutic delivery systems. With growing interest in EVs from microbes and probiotics, engineering EVs with improved loading of antioxidants, targeting of different tissues, and release response to certain stimuli might further boost the development of precision redox therapeutics for the chronic wound healing process.

### 4.4. Emerging Interest in Microbial and Probiotic-Derived Extracellular Vesicles

While most existing research on EVs has concentrated on vesicles from mammalian cells, such as MSC-EVs, evidence of the active secretion of biologically functional vesicles from microorganisms to facilitate host–microbe communication is growing [26,27]. This concept has recently become a subject of study in the fields of regenerative medicine and studies on inflammatory diseases because EVs derived from microbes seem to be able to impact immune regulation, epithelial barrier function, metabolic signaling, and oxidative stress responses in the host tissues. Significantly, beneficial microorganisms (commensals and probiotics) have arisen as particularly interesting sources of bioactive EVs that may have therapeutic implications in chronic inflammatory and oxidative stress-related diseases.

Microbial-derived EVs exhibit a number of conceptual benefits as cell-free signaling systems as compared to conventional probiotic therapy involving the delivery of living microorganisms. The nanoscale, biological stability, and lack of replication of these vesicles could enable the delivery of bacterial bioactive molecules and decrease the issues associated with uncontrolled bacterial colonization and/or translocation [210,211]. In addition, preliminary evidence indicates that EVs from probiotics have the potential to influence several redox-sensitive pathways related to inflammation, mitochondrial stress, macrophage polarization, as well as tissue repair [162,212]. Moreover, their compatibility with bioengineering and synthetic biology has led to a growing interest in the potential design of antioxidant, immunomodulatory, or regenerative, programmable microbial vesicles [213,214].

In this context, EVs derived from probiotics are emerging as an exciting new group of nanotherapeutics associated with the microbiome for redox regulation and for wound healing. Below, we will thus concentrate on the biology, molecular content, and therapeutic applications of probiotic-EVs, and their key role in the modulation of oxidative stress and chronic wound repair.

## 5. Probiotic-Derived EVs: Characteristics and Biological Potential

### 5.1. Sources and Biological Characteristics of Probiotic-Derived EVs

Probiotic-EVs are nanosized membrane vesicles that are spontaneously released by beneficial microorganisms and have been emerging as key mediators in the microbiota–host dialogue. Like mammalian EVs, these vesicles act as vehicles to deliver various types of molecular cargoes from the parental bacteria to other microbial organisms or even to host cells [131,215,216,217]. Probiotic-EVs have been observed to exhibit many beneficial properties classically associated with live probiotics in recent years and may not encounter some of the issues found in taking live cells. Numerous species of probiotic and commensal bacteria, such as *Lactobacillus*/*Lactiplantibacillus*, *Bifidobacterium*, *Akkermansia muciniphila*, *Bacillus*, *Lacticaseibacillus,* and other lactic acid bacteria associated with gut and mucosal homeostasis, have already been reported to produce EVs [211,218,219,220,221,222,223]. Of these, the genera *Lactobacillus* and *Lactiplantibacillus* have been studied the most due to their proven anti-oxidative, immunomodulatory and epithelial protective properties [211,221,224,225,226]. More recently, next-generation probiotics like *A. muciniphila* have, however, also been identified as important EV-producing bacteria which potentially play significant roles in the control of inflammatory processes, metabolic homeostasis and modulation of oxidative stress [219,222,227].

While the secretion of EVs was originally believed to be exclusive to Gram-negative bacteria, where they are formed by blebbing of the OM, increasing evidence now shows that Gram-positive probiotics are also able to release large quantities of nanoscale EVs through a yet unidentified mechanism that does not involve outer membrane blebbing in view of the thick peptidoglycan cell wall [211,228,229]. The exact mechanisms that lead to the formation of EVs in Gram-positive bacteria are still not fully understood, but there are several different mechanisms that have been suggested, such as stress response due to membrane curvature, reduction of the cell wall by autolysin, localized remodeling of peptidoglycan, and membrane extrusion due to turgor pressure [228,230,231]. Additional stress conditions present in the environment, such as oxidative stress, acidic pH, nutrient limitation, and inflammatory signaling, could further contribute to an increase in the number of vesicles produced or changes in the contents of EVs. Importantly, recent research has shown that the process of EV release from probiotics is not a passive secretion process but an adaptive one, which allows bacteria to communicate with host tissues and alter their microenvironments under stress situations [216,218,232,233,234]. In particular, this may occur in inflammatory conditions and oxidative stress, like chronic wounding, where the symbiotic adaptations of microorganisms and the host–microbiota relationship may play a dynamic role in tissue repair.

Probiotic-EVs are highly diverse and have varying cargo compositions, which seem to be determinant factors of their biological activity and are dependent on the environmental conditions and the species of bacteria they are derived from. So far, the evidence suggests that vesicles derived from probiotics contain a mix of membrane lipids and phospholipids, lipoteichoic acid-associated structures, proteins, enzymes, metabolites, polysaccharides, and nucleic acids that may contribute to the modulation of host signaling pathways [212,218,221,225,235]. Various bioactive proteins have been detected in probiotic-EVs: stress-response proteins, adhesion molecules, metabolic enzymes, antioxidant- associated proteins, and proteins that modulate the immune response at the epithelial barrier and the inflammatory response [212,236,237]. Additionally, probiotic-EVs can carry molecules related to redox and metabolic homeostasis that are associated with SCFAs, such as organic acids, small bioactive peptides, or molecules related to bacteriocins [238,239]. However, in this regard, there has been a growing interest in the nucleic acid cargoes in probiotic-EVs, including microRNA (miRNA)-like molecules, regulatory RNAs, and bacterial small RNAs (sRNAs) that are potential regulators in cross-kingdom communication with host cells [240,241]. There is growing evidence that these nucleic acid payloads may be associated with the modulation of inflammation, response to oxidative stress, mitochondrial activity, macrophage polarization, and/or epithelial barrier integrity at a post-transcriptional level [212,242,243,244,245,246]. However, direct cargo-specific validation remains limited for probiotic-derived EVs. These results provide a unified view that EVs derived from probiotics are not just “bacterial byproducts” but are intricate nano-organized structures that may participate in microbiota–host signaling networks relevant to redox regulation, immune homeostasis, and tissue repair mechanisms.

### 5.2. Immunomodulatory and Antioxidant Functions of Probiotic-Derived EVs

There is now emerging evidence that EVs from probiotics may exert immunomodulatory and redox-associated effects on several host cell populations associated with tissue remodeling, including keratinocytes, fibroblasts, macrophages, endothelial cells, and epithelial barrier cells [212,216,232,244,247,248,249]. Like their parental probiotic microorganisms, probiotic-EVs have been associated with changes in several components of the oxidative and inflammatory microenvironment, including ROS signaling in cells, generation of inflammatory cytokines, mitochondrial function, integrity of the epithelial barrier, and cellular regenerative responses [212,244,250]. In particular, as EVs are nanoscale, concentrated microbial bioactive cargoes, they could be more specifically and precisely targeted in some contexts than the delivery of live probiotics. As summarized in Table 4, existing studies focusing on probiotic-EVs in wound healing and oxidative stress-related tissue injury vary in EV preparation and characterization, cargo assessment, treatment strategies, experimental models, and the extent of mechanistic validation. Collectively, these studies suggest potential involvement of probiotic-EVs in redox modulation, ROS-sensitive signaling, inflammatory and reparative macrophage responses, and tissue regeneration, although the strength of evidence for these mechanisms varies across studies. However, there is still a small amount of evidence, mainly preclinical. Furthermore, the level of mechanistic support differs among studies, as many of the conclusions are drawn on the basis of ROS measurements, cytokine changes, or pathway markers.

Regarding redox modulation, several studies have shown that EVs derived from probiotics are associated with decreased intracellular ROS levels and changes in antioxidant defenses. Several vesicles from different species of *Lactobacillus* (*L. plantarum*, *L. rhamnosus GG,* and *L. reuteri*) demonstrated their ability to exert antioxidant activities in a variety of epithelial injury, inflammatory stress, and wound repair models. For instance, EVs produced by *L. plantarum* have been associated with improved keratinocyte migration and re-epithelialization, together with increased anti-inflammatory IL-10, decreased pro-inflammatory cytokines such as TNF, reduced neutrophil infiltration, and increased M2-like macrophage polarization [93]. These immunoregulatory properties might indirectly contribute to the reestablishment of redox homeostasis and the establishment of a more favorable microenvironment for tissue repair in chronic wounds, as persistent inflammation therein is a primary source of excess ROS generation. In the same way, biomimetic hydrogels containing probiotic membrane vesicles showed strong ROS-scavenging activity, in addition to faster wound healing, increased angiogenesis, and collagen deposition in wounds of the skin, respectively [92,95,96]. Other studies also indicated that probiotic-EVs may not only inhibit the production of extracellular ROS but could also reduce mitochondrial oxidative stress [92]. Interestingly, vesicles derived from *L. reuteri* have been associated with mitochondrial metabolism and oxidative phosphorylation in macrophages, together with reduced mitochondrial dysfunction and improved healing outcomes of wounds at mucosa and skin [92,95]. All these observations indicate that probiotic-EVs might not only work as passive vehicles to carry antioxidant elements but also have an impact on cellular redox-related processes. The effects, however, could also be due to direct regulation of specific redox pathways and will need to be confirmed by pathway-specific mechanistic studies.

Probiotic-derived EVs seem to have the ability to affect more than one redox-sensitive chronic wound-signaling pathway that is interconnected. Multiple studies reported changes consistent with increased Nrf2/HO-1-associated antioxidant signaling and decreased NF-κB-associated inflammatory signaling, suggesting the possible involvement of these pathways in the tissue-protective effects of probiotic-EVs [94,96]. For example, EVs from *L. reuteri* were associated with increased Nrf2-related cytoprotective signaling, together with decreased oxidative damage and inflammatory responses in epithelial tissues [96]. Likewise, probiotic-EV systems with inflammation-responsive hydrogels or bioengineered biomaterials presented coordinated control of the pathways of oxidative stress and enhanced tissue repair [96]. Recent studies with EVs derived from *L. rhamnosus GG* further showed that vesicle-associated miR-21-5p was associated with enhanced re-epithelialization and angiogenesis, suggesting possible involvement of EV cargo in post-transcriptional modulation of host repair programs [94]. If cargo-depletion, inhibition or rescue experiments have not been performed, such associations should not be taken as proof of cargo-specific causality. In addition, mitochondrial ROS regulation, suppression of inflammatory cytokines, and macrophage-associated signaling pathways have been considered part of the mechanisms of probiotic-EVs-mediated regenerative activity [92]. These results suggest that probiotic-EVs may influence multiple interconnected biological processes associated with antioxidant, immunomodulatory, angiogenic, and epithelial reparative responses. These pathway-level conclusions should be approached with prudence, as not all studies that are available and have focused on a specific probiotic-EV pathway have directly validated the results through pathway-specific inhibition, genetic perturbation, cargo-depletion, or rescue experiments.

Another prominent mechanistic trend among the probiotic-EV studies included was immune modulation, which included the reduction of pro-inflammatory cytokines and the induction of reparative phenotypes of macrophages. Multiple probiotic-EV systems were discovered to mediate a decrease in the levels of inflammatory mediators such as TNF-α, IL-1β, and IL-6, as well as an increase in the levels of anti-inflammatory mediators such as IL-10 and M2 macrophages [92,93,96]. These effects are especially pertinent at the level of chronically inflamed wounds, such as those where inflammation is continually tumultuous and oxidative amplification is happening. Beyond macrophage regulation, probiotic-EVs were also consistently linked to the preservation of the integrity of the epithelial barrier, enhancement of keratinocyte migration, promotion of angiogenesis, and improvement of tissue remodeling responses [95]. Notably, a few studies indicated that probiotic-EVs may help to promote co-regulation of epithelial and immune cells, consequently helping to alleviate inflammatory injury and promote regenerative repair processes. The immunomodulatory effects are likely to be extremely strain-specific and highly context-dependent due to the type of cargo molecules EVs carry, which depends on the physiology and the bacterial source of the probiotics. Furthermore, the immunomodulatory and regenerating effects might be strain-, cargo-, model-, and delivery-dependent, and direct comparison across different studies is challenging due to different EV isolation, purification, characterization, doses, and experimental designs.

Moreover, despite the lower immunogenicity of Gram-positive vesicles derived from probiotics than those of pathogenic bacteria, the potential of these vesicles as immunogens based on peptidoglycan has not been fully investigated, and the batch-to-batch variability of vesicles produced from Gram-positive bacteria, as well as the biodistribution and biosafety over time, have yet to be fully evaluated. Notably, to date, there are no human clinical studies that directly evaluate probiotic-EV-mediated antioxidant therapy in wound healing. Thus, although the studies listed in Table 4 collectively support the emerging therapeutic potential of EVs derived from probiotics as microbiome-associated redox modulators, additional mechanistic validation, standardized characterization of EVs, chronic wound modeling, and safety assessment studies will be critical before translation to clinical wound care applications.

### 5.3. Comparative Positioning of Probiotic- and MSC-Derived EVs

Although the field of EVs as therapeutics is still in its infancy and undergoing rapid advances, MSC-EVs are the most widely investigated EVs in the wound healing and regenerative medicine fields [251,252]. However, probiotic EVs have recently been recognized as a unique type of microbiome-derived vesicle and may possess distinct biological and translational properties that are fundamentally different from mammalian EV systems. Probiotic-EVs and MSC-EVs do not necessarily represent competing technologies, but they seem to convey complementary therapeutic properties that could ultimately be used for different aspects of chronic wound management. Notably, multiple manufacturing and scale-up benefits have been proposed for probiotic-EVs but have yet to be shown in GMP-grade production of clinical-grade probiotic-EV therapeutics. Table 5 succinctly outlines some of the key differences and proposed advantages and limitations of the two EV platforms in terms of their biological origin, cargo makeup, degree of engineering flexibility, and way of interacting with tissues, as well as their level of maturity for translation.

The most important difference between them, which can be noticed, is with regard to the source of vesicle production. The source of MSC-EVs is mammalian stem cells such as bone marrow, adipose, and umbilical cord-derived MSCs, while the source of probiotic-EVs is beneficial microorganisms such as *Lactobacillus*, *Lactiplantibacillus*, *Bifidobacterium,* and *Akkermansia muciniphila* [211,218,219,222,253,254]. This difference has a strong impact on their molecular composition, what they carry, and their downstream biological activities. MSC-EVs frequently contain many proteins and growth factors involved in tissue remodeling and cellular regeneration, anti-inflammatory cytokines, and host-derived regulatory microRNAs (miRNAs) [255,256]. In contrast, EVs derived from probiotics carry molecules associated with the microbiota, such as proteins related to stress, antioxidant-associated enzymes, stress-responsive proteins, SCFA-related compounds, and bacterial regulatory RNAs that may be associated with the modulation of host redox and immune pathways [212,257]. Thus, MSC-EVs have comparatively more extensive preclinical evidence for regenerative and pro-angiogenic effects, whereas probiotic-EVs may provide distinct microbiome-associated immunometabolic and redox-regulatory properties [212,251,255,258].

The two EV systems are also different with respect to their engineering potential and delivery characteristics. In preclinical wound model systems, MSC-EVs have shown good regenerative efficacy and are more advanced in clinical translation [251,259]. However, bacterial systems are amenable to genetic programming, which may provide opportunities for engineering parental probiotic strains and their derived EVs [122,260]. Potential advantages of applying synthetic biology strategies to probiotic-EV systems include: programmable and customizable loading of cargo, targeted modification of EV surfaces, environment-responsive release systems, and programmable redox modulation strategies [261,262]. Furthermore, these probiotic-EVs may be particularly relevant to the oral mucosa, or even microbiota-oriented therapeutic approaches due to their natural role in host–microbiome communication networks [232,263]. Such properties may be especially pertinent for chronic inflammation and metabolic diseases in which the gut–skin axis is dysregulated and systemic oxidative stress has arisen.

Of particular interest, probiotic-EVs and MSC-EVs could eventually be more effective as complementary therapeutic strategies instead of mutually exclusive alternatives. Although vesicles derived from MSCs might have substantial potential for regenerative support of angiogenesis, ECM remodeling, and tissue reconstruction, vesicles derived from probiotics might enable complementary signaling functions related to the modulation of immunity and redox balance as well as the microbiome [211,218,264,265]. The combination of these two EV types with biomaterials or responsive nano-delivery systems might therefore be helpful for future therapeutic strategies aiming toward even more comprehensive modulation of the microenvironment of chronic wounds. It is not simply appropriate that EVs from probiotics are not only considered as an alternative to mammalian EVs, but also that EVs from microorganisms can be considered a distinct emerging class of nanotherapeutic platforms with potential biological properties concerning redox-oriented regenerative medicine.

**Table 5:** Comparative characteristics and translational considerations of probiotic-derived EVs and MSC-derived EVs for wound-healing applications.

Feature	Probiotic-Derived EVs	MSC-Derived EVs
**Source**	Beneficial gut or skin-associated probiotics (e.g., *Lactobacillus*, *Lactiplantibacillus*, *Akkermansia muciniphila*, *Bifidobacterium*)	MSCs (e.g., bone marrow, adipose tissue, umbilical cord)
**Manufacturing Cost**	Potentially low; may be more amenable to bacterial culture-based production, although GMP-grade manufacturing cost has not been established	Generally associated with more complex mammalian cell culture and GMP-grade production requirements
**Production Stability**	Theoretically scalable through bacterial culture-based production; clinical-grade scalability and batch consistency remain to be demonstrated	May be affected by donor variability, cell state/senescence, culture conditions, and batch-to-batch variation
**PAMP/MAMP-associated Immunogenicity**	Potential innate immune activation by MAMPs/PAMPs, including peptidoglycan, lipoteichoic acid, lipoproteins, and, where applicable, LPS-associated components; effects are strain-, cargo-, purity-, and dose-dependent	Generally lower PAMP/MAMP-related immunogenicity, although donor-, culture-, cargo-, and product-related immune effects still require evaluation
**Major Antioxidant Cargo**	Bacterial enzymes, stress proteins, SCFA-associated metabolites, thioredoxin-related proteins, regulatory sRNAs	miR-21, miR-146a, antioxidant enzymes (SOD, catalase), HO-1-related signaling molecules
**Immunomodulatory Functions**	Regulation of TLR/NF-κB/Nrf2 pathways; promotion of M2 macrophage polarization	Suppression of excessive inflammation; enhancement of M2 macrophage polarization and fibroblast activation
**Barrier Penetration/Wound Delivery**	Small nanoscale vesicles facilitate tissue penetration and cellular uptake; frequently incorporated into hydrogels or other biomaterial platforms for enhanced wound retention	Effective delivery through local injection, topical application, and biomaterial-assisted delivery systems
**Engineering Flexibility**	Potentially amenable to parental-strain genetic engineering and programmable cargo modification; direct validation in therapeutic probiotic-EV manufacturing remains limited	Established preclinical strategies include genetic modification, cargo loading, and surface functionalization Can be engineered for siRNA/protein loading and surface targeting modifications
**Oral/Noninvasive Administration Potential**	Theoretical potential for oral or other noninvasive delivery and microbiota-mediated systemic effects; biodistribution and therapeutic efficacy remain insufficiently validated	Primarily administered through local injection or topical application
**Gut–Skin Axis Potential**	Potential to participate in microbiota–gut–skin communication pathways, but direct wound-healing evidence remains limited	Limited evidence supporting gut–skin axis modulation
**Clinical Maturity**	Early preclinical stage; wound-healing evidence remains limited and no direct human therapeutic validation is currently available	More advanced preclinical and early clinical development across regenerative indications, although clinical validation for wound healing remains limited
**Regulatory Classification**	No dedicated classification established; may intersect with biologic, microbial/postbiotic, nanomedicine, or engineered EV product frameworks depending on composition and intended use	Generally considered within biologic/EV therapeutic frameworks, although product classification and regulatory requirements remain jurisdiction- and product-dependent
**Major Advantages**	Potentially lower-cost bacterial culture-based production, theoretical scalability, engineering potential, and microbiome-associated biological properties	More extensive regenerative and wound-healing evidence and greater translational maturity
**Major Limitations**	Limited direct human evidence, insufficient clinical-grade manufacturing data, EV and cargo heterogeneity, PAMP/MAMP-associated immunogenicity, and unresolved regulatory classification	High production cost, donor variability, limited scalability
**Potency Assays**	No standardized wound-specific potency assay established; mechanism-relevant assays may include redox, immunomodulatory, epithelial, or angiogenic endpoints	More extensively developed functional assays are available, but standardized indication-specific potency assays remain an important translational requirement
**Quality Control Requirements**	Requires standardized strain/culture conditions, EV isolation and purification, physicochemical characterization, cargo assessment, sterility/contaminant testing, and functional potency testing	Requires standardized donor/cell-source qualification, culture conditions, EV isolation and characterization, sterility, identity/purity assessment, and potency testing
**Batch-to-Batch Reproducibility**	Potentially affected by bacterial strain, growth phase, culture conditions, isolation method, and cargo heterogeneity; clinical-grade reproducibility remains unestablished	Affected by donor variability, cell state, culture conditions, passage, isolation method, and manufacturing process; substantial standardization efforts are ongoing
**References**	[122,211,212,218,219,222,232,257,258,260,261,262,263]	[251,252,253,254,255,256,259,264,265]

Abbreviations: EVs: extracellular vesicles; GNP: guanosine monophosphate; HO-1: heme oxygenase-1; LPS: lipopolysaccharide; MSC: mesenchymal stem cells; NF-kB: nuclear factor kappa B; Nrf2: nuclear factor erythroid 2-related factor 2; SCFA: short-chain fatty acids; SOD: superoxide dismutase; TLR: toll-like receptors; MAMPs: microbial-associated molecular patterns; PAMPs: pathogen-associated molecular patterns.

### 5.4. Translational Opportunities and Current Limitations of Probiotic-Derived EVs

Overall, the available evidence suggests that EVs derived from probiotics could be a new generation of probiotic-based therapeutic agents in promoting wound repair via an antioxidant mechanism. Table 6 summarizes important differences between probiotic-EVs and live probiotics, covering their structure, delivery system, tissue-penetrating potential, biosafety profile, and engineering potential [27]. The unique advantages of being cell-free and non-replicative allow probiotic-EVs to possibly diminish issues related to live bacteria therapy, such as uncontrolled colonization, bacterial translocation, and infection risk in susceptible individuals [34,232]. The other reason is that their nano size may facilitate interactions with and distribution within wounded tissues, and protect active contents during extracellular transport [266]. The properties described above underpin the developing idea that probiotic-EVs could be even more controllable and drug-adaptable postbiotic vehicles than the administration of only live microorganisms.

A second major translational benefit is the ability to manufacture them easily and their compatibility with high-tech delivery systems. Probiotic-EVs can be potentially manufactured via scalable bacterial culture systems and in combination with hydrogels, wound dressings, microneedles or ROS-responsive biomaterials, where they can be retained in the tissue and release their therapeutic effects gradually in oxidative environments in wounds [267,268,269,270]. Simultaneously, the engineering flexibility of the microbial system may provide opportunities for programmable EV design options, such as selective antioxidant cargo enrichment, inflammation-responsive release, and targeted modulation of wound-associated signaling pathways [203,271,272]. All of these could be useful characteristics for chronic wounds, where a combination of oxidative stress, chronic inflammation, microbial dysbiosis, and the lack of tissue regeneration exists in highly heterogeneous microenvironments.

Although these are promising features, there are still some significant drawbacks that hinder clinical translation. The isolation and purification procedures for EVs are still not well standardized, and significant differences in probiotic-EV preparations have been reported based on the bacterial strain, culture conditions, purification methods, and EV cargo [273,274]. One other point of uncertainty is the potential for immunogenicity of bacterial vesicles, such as those linked to peptidoglycan, lipoteichoic acid, or endotoxin-like inflammatory signaling pathways [275,276,277,278]. Furthermore, this evidence is still scarce for most of the existing systems and consists of in vitro systems and short-term animal studies, and there are virtually no robust chronic wound models or clinical data from humans available. The long-term biodistribution and pharmacokinetics, as well as tissue specificity and biosafety profiles, are also not fully characterized. Furthermore, there is a lack of regulatory considerations, as microbial-derived nanovesicle therapeutics have yet to be well regulated for quality control, large-scale production, and clinical applications.

Taken together, these studies highlight the potential of probiotic-derived EVs as an emerging yet very promising therapeutic platform that is at the nexus of microbiome science, redox biology, regenerative medicine and bioengineering. While a lot of work still needs to be performed on the translational front, the new possibilities that synthetic biology and biomaterial-assisted delivery technologies have brought, along with the advancement of nanomedicine, could greatly improve the chances of future development of probiotic-EV-based therapeutics for the management of chronic wounds.

**Table 6:** Comparison between live probiotics and probiotic-derived extracellular vesicles in wound healing applications.

Feature	Live Probiotics	Probiotic-EVs
**Basic Structure**	Viable microorganisms containing intact cell walls, cytoplasmic components, and genomic DNA/RNA	Cell-free nanosized lipid bilayer vesicles containing bioactive cargo
**Mechanism of Action**	Act through colonization, microbial competition, metabolite secretion, and host immune modulation	Deliver functional cargo directly to host cells, including proteins, lipids, RNAs, and metabolites
**Redox-Regulatory Capacity**	Certain strains produce antioxidant metabolites such as SCFAs and GSH	EVs can directly transport antioxidant enzymes and redox-regulatory molecules (e.g., SOD-like proteins, miRNAs)
**Immunomodulatory Effects**	Activate TLR/NOD-associated signaling and influence macrophage polarization	Modulate immune responses through vesicle-associated surface molecules and regulatory RNA cargo
**Routes of Administration**	Oral, topical, spray-based, or local administration	Topical delivery, spray application, or incorporation into hydrogels and biomaterials
**Penetration across Wound Barriers**	Limited by bacterial size and cell wall structure	Small vesicle size (~50–200 nm) may facilitate tissue penetration and cellular uptake
**Requirement for Survival/Colonization**	Require viability and interaction with local microbiota for sustained activity	Non-replicative; therapeutic effects mediated through direct cargo delivery
**Engineering Potential**	Genetically engineerable strains capable of expressing therapeutic factors (e.g., FGF2, CXCL12)	EV engineering remains early-stage but allows programmable cargo loading and surface modification
**Safety Considerations**	Potential risks of bacterial translocation, infection, or horizontal gene transfer	Reduced infection risk due to acellular nature; lower likelihood of uncontrolled colonization
**Regulatory Challenges**	Often regulated within probiotic, food, or live biotherapeutic frameworks	Greater standardization and characterization challenges as microbial nanovesicle therapeutics
**Research Maturity**	Supported by multiple clinical studies, including dermatologic and gastrointestinal applications	Predominantly preclinical evidence from cell culture and animal studies
**References**	[27,34,270]	[93,273,274]

Abbreviations: CXCL: C-X-C motif chemokine ligand; EVs: extracellular vesicles; FGF: fibroblast growth factors; GSH: glutathione; NOD: nucleotide-binding oligomerization domain; SCFAs: short-chain fatty acids; SOD: superoxide dismutase; TLR: toll-like receptor.

## 6. Engineering and Therapeutic Optimization of Probiotic-Derived EVs

### 6.1. Bioengineering Strategies for Probiotic-Derived EVs

**Figure 3:**
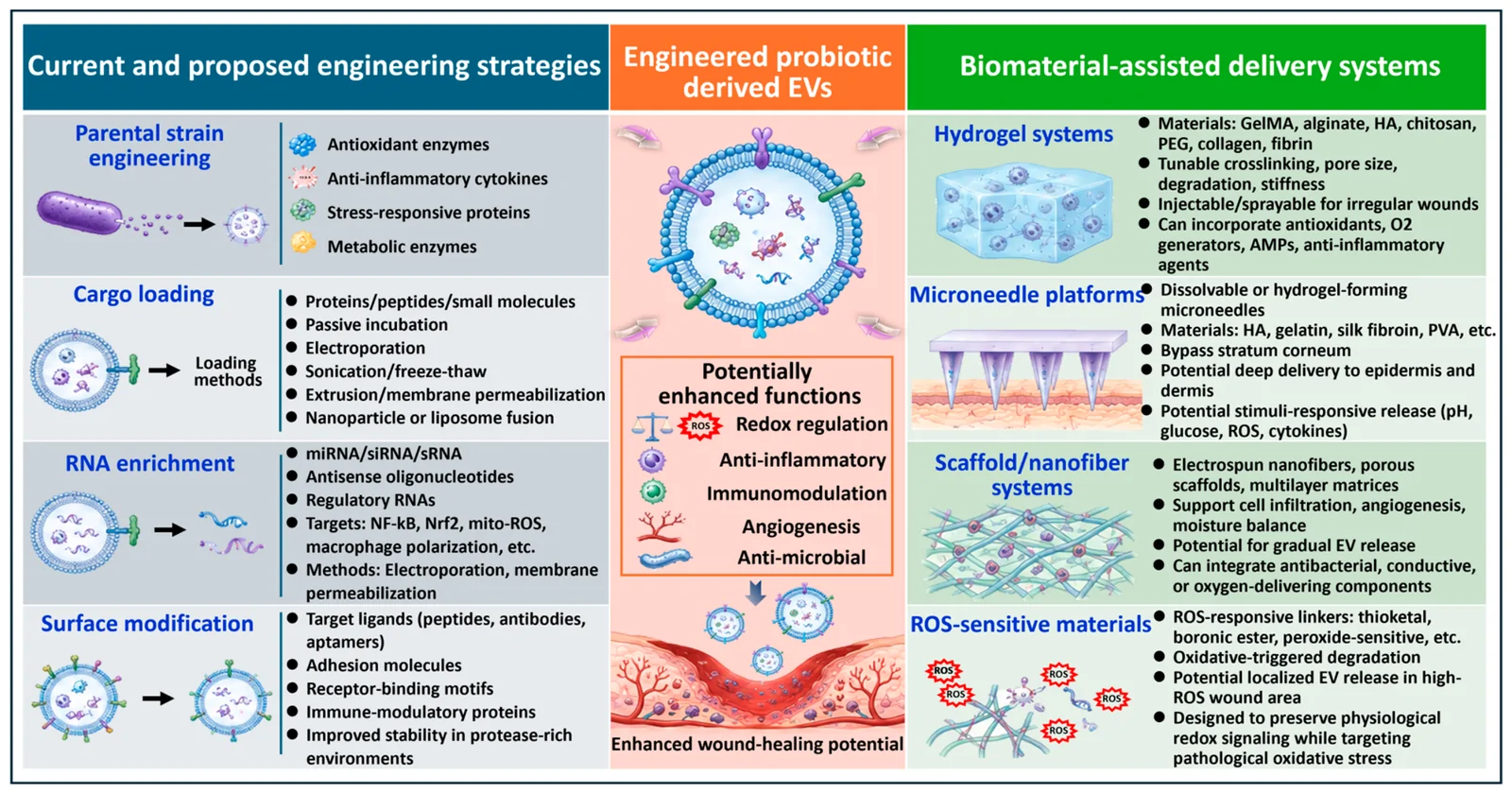
Current and proposed engineering strategies and biomaterial-assisted delivery platforms for probiotic-derived extracellular vesicles in redox-regulated wound healing. Overview of engineering approaches and delivery strategies that may enhance the therapeutic performance of probiotic-derived extracellular vesicles (EVs) for oxidative wound repair. On the left, four major engineering routes are illustrated, including parental strain engineering, cargo loading, RNA enrichment, and surface modification. These approaches have the potential to modify EV cargo composition, redox-regulatory capacity, cellular uptake, and tissue specificity. On the right, biomaterial-assisted delivery systems, including hydrogels, microneedle platforms, bioactive scaffolds, nanofiber matrices, and ROS-responsive materials, are presented as strategies that may improve EV stability, tissue retention, penetration, and controlled release. Importantly, several of these approaches have been more extensively explored in broader EV or biomaterial systems, whereas their specific application to probiotic-derived EVs in chronic wound models remains emerging and requires further validation. Collectively, these engineering and delivery concepts provide a framework for the future development of probiotic-EV-based redox therapeutics.

Nevertheless, with the rising interest in EVs and their use in probiotic applications, researchers have started to move away from simply characterizing vesicles naturally secreted by the probiotics and have started to actively modify these vesicles for application in therapy. While it is well established that native probiotic-EVs have naturally inherent antioxidative and immunomodulatory effects, whether their natural cargo is sufficient for highly oxidative and/or chronically inflammatory wound conditions remains unclear [94,232]. This has led to the exploration of various engineering strategies to improve the functionality of EVs, cargo delivery, and redox-regulatory activity in damaged tissues. There are several practical benefits of using probiotic microorganisms as EV systems as compared to mammalian systems; bacterial strains are easy to culture, genetically modify, and scale up to produce EVs in large quantities and are readily available. As summarized in Figure 3, current and proposed strategies for optimizing probiotic-derived EVs can be broadly divided into two complementary approaches: EV bioengineering and biomaterial-assisted delivery. EV bioengineering mainly encompasses parental strain engineering, cargo loading, RNA enrichment, and surface modification; biomaterial-assisted approaches, such as hydrogels, microneedles, scaffolds/nanofiber systems, and ROS-responsive materials, are engineered to enhance EV stability, retention, tissue penetration, and controlled release within the wound microenvironment. Notably, there is significant variation in the experimental evidence in support of these strategies. Throughout this section, we differentiate between strategies that have already been shown with probiotic-derived EVs in wound or tissue-repair models, those that have been demonstrated in general bacterial-EV systems, those that have been extrapolated from mammalian-EV research, and those that are conceptual or prospective for probiotic-derived EV-based wound healing.

1. **Parental engineering:** Currently, one potential approach to change the composition of EVs in probiotics is the so-called parental engineering. As the physiological state and genetic information of the EV cargo largely derive from the parental microorganism, it is possible to selectively increase the level of therapeutic molecules in the vesicles by engineering the bacteria [279,280,281]. However, in practice, parental bacterial strains may potentially be genetically engineered to produce an abundance of antioxidant enzymes, anti-inflammatory mediators, proteins involved in stress reactions, or peptides with a regeneration capacity that, during the biogenesis of vesicles, may be incorporated into the EV cargo [35,282,283]. Various studies in engineered microbial or related experimental systems have addressed the use of catalase, SOD, thioredoxin-related proteins, or heme oxygenase-related pathways to enhance ROS-scavenging activity in inflammatory tissues [284,285,286]. In a similar fashion, engineered probiotics can be used to produce proteins that could modulate macrophage polarization, epithelial repair, or mitochondrial oxidative homeostasis [123,287,288,289,290,291]. Parentally engineered EVs may offer a stable and scalable approach to generating EVs with a more consistent therapeutic cargo profile in comparison with EVs generated by post-isolation modification. This approach has yet to be tested in the context of EVs derived from probiotics in chronic wound models, however.
2. **Cargo loading:** Many strategies of EV engineering have been explored primarily in mammalian-EV and broader EV systems, and these may also be applicable to probiotic-EVs. After EVs have been isolated, therapeutic molecules can be loaded into EVs, along with the endogenous bacterial load to give EVs an extra boost in biological activity. Incorporation of antioxidant proteins or peptides or small-molecule therapeutics into the interior of vesicles can be achieved by protein loading techniques such as passive incubation, membrane permeabilization, sonication, freeze-thaw cycling, extrusion or electroporation [292,293,294,295]. In addition, integration of EVs with biomimetic nanoparticles or liposomal fusion systems has been explored in broader experimental systems for more effective encapsulation and delivery stability [296,297,298]. However, the therapeutic advantages of these cargo-loading approaches remain to be validated specifically in probiotic-EV-based chronic wound models. These methods may be especially useful in scenarios where the concentration of endogenous bacterial cargo is too low to be used for therapy. Importantly, the cargo loading strategies also provide opportunities for integrating more than one therapeutic function in a single vesicle platform, such as antioxidative, anti-inflammatory, antimicrobial, and regenerative functions.
3. **RNA enrichment:** Another emerging strategy with potential application to probiotic-EVs is RNA engineering. It is now becoming abundantly clear that sRNA, microRNA, and other nucleic acid cargoes are naturally present in EVs produced by bacteria and are able to modulate inflammatory and oxidative signaling pathways in the host [299,300]. Based on this idea, a number of engineering strategies have been developed to selectively enrich EVs or bacterial vesicles with therapeutic RNA, although their application to probiotic-derived EVs remains less established. These include loading with miRNAs, siRNAs, antisense oligonucleotides, or other regulatory RNAs that could be used to regulate NF-κB activation pathways, Nrf2 signaling pathways, mitochondrial ROS production pathways, or macrophage polarization pathways [301,302]. Among the most popular approaches to transferring nucleic acid cargo to isolated vesicles are electroporation and permeabilization of membrane vesicles through the addition of membrane permeabilizing agents [303,304,305]. While the efficiency of loading EVs with RNA and the stability of EVs are still technical issues to be resolved, this suggests the potential for probiotic-EVs to serve as a biologically compatible nanoscale RNA delivery system for targeting redox modulation. However, there is still limited direct validation of RNA-engineered probiotic-EVs for redox modulation in chronic wounds.
4. **Surface modification:** Surface engineering and the incorporation of vesicles by display modification have also been explored as methods for the improvement of EV targeting and interaction with cells. A number of studies have investigated the possibility of engineering the surface of bacterial EVs using targeting ligands, adhesion peptides, receptor-binding motifs and/or immune-modulatory surface proteins, as these surface components strongly impact the vesicle’s tropism, immune recognition and uptake by the cells [159,306]. For instance, changes to the surfaces of vesicles might be made to optimize reactions with inflamed endothelial tissue, activated macrophages, damaged epithelium, or the regions of wounds with high ROS levels [307,308,309]. Surface display engineering may additionally be used to enhance EV stability in wound settings, where protease concentrations are high, or to help with the incorporation of EVs into biomaterial-assisted delivery systems, like hydrogels and scaffolds [310,311,312]. Many of the strategies still exist at the very early stages of preclinical research, but overall, they illustrate the potential for rational engineering of EVs derived from probiotics to optimize the composition of EVs, enhance targeting to cells and tissues, and maximize therapeutic potential through manipulation of redox properties within the wound microenvironment. Most of these approaches have been investigated in bacterial-EV or nanovesicle systems and have yet to be tested specifically for probiotic-EVs in chronic wounds.

### 6.2. Biomaterial-Assisted Delivery Systems

Despite the huge therapeutic potential of EVs derived from probiotics, one of the main challenges in this area is the retention of these nanoscale vesicles in the wound environment in order to have a sustained therapeutic effect. Chronic wounds include wounds in which there is an excessive amount of exudate, a protease-rich environment, a persistent inflammatory process, biofilm formation, and frequent mechanical disruption that can quickly destabilize EVs and reduce local retention after they are directly introduced into the wound [8,313,314]. Therefore, the combination of probiotic-EVs with biomaterial-assisted delivery systems that enhance the protection of vesicles, their localization in tissues, controlled release, and cell internalization has emerged as a potential research direction. While these delivery platforms have been extensively studied in general EV and regenerative medicine systems, including mammalian-EV delivery, their use for EVs derived from probiotics is comparatively limited. Current biomaterial-assisted delivery approaches are mainly composed of hydrogels, microneedle platforms, bioactive scaffolds or nanofiber systems, and ROS-responsive materials that focus on enhancing EV stability, local retention, tissue penetration, and controlled release in oxidative wound microenvironments (Figure 3). Four of five studies identified in this review that directly tested probiotic-derived EVs for wound healing or tissue repair used hydrogel-assisted delivery, including one ROS-responsive system, and one directly administered EVs without a biomaterial carrier. Thus, hydrogel-assisted delivery currently has the most direct preclinical support among the biomaterial strategies discussed below, while microneedle- and scaffold-based approaches remain largely extrapolated from broader EV and nanomedicine platforms.

1. **Hydrogel:** Of these biomaterial approaches, hydrogel-assisted delivery has been directly examined using EVs derived from probiotics or probiotic membrane vesicles in preclinical wound/tissue-repair models [92,93,95,96]. In contrast, most of the advanced designs of hydrogels and multifunctional or responsive hydrogels below are adapted from more general studies of EVs and biomaterial delivery and have yet to be directly tested with EVs derived from probiotic bacteria. Hydrogel-based delivery systems are among the most widely studied approaches for localized EV delivery in broader EV and regenerative medicine research. Due to their high water content, structural flexibility, and tunable physicochemical properties, hydrogels can mimic aspects of the native ECM and could also serve as reservoirs for sustained EV release [311,315,316]. Probiotic-EVs could be encapsulated in natural or synthetic hydrogel matrices containing compounds like gelatin methacrylate (GelMA), alginate, hyaluronic acid, chitosan, polyethylene glycol (PEG), collagen or fibrin-based polymers in practice [317,318,319]. Then, release kinetics can be further tuned by tuning the crosslinking density, degradation rate, pore size, and stiffness of the hydrogel [320,321,322]. Injectable and sprayable hydrogel formulations have been investigated as well, which might be helpful for delivering the formulation into irregularly shaped sites and reducing tissue damage during delivery [323,324,325]. Multifunctional hydrogels can be loaded with antioxidant reagents, antimicrobial peptides, oxygen-producing compounds, conductive polymers, or anti-inflammatory molecules that could potentially act synergistically with the actions of the probiotic EVs to favor tissue regeneration and/or local redox balance [326,327,328]. However, these multifunctional combinations are yet to be confirmed in wound models using probiotic EVs.
2. **Microneedle:** Microneedle-assisted delivery systems are another promising way in which to increase EV penetration into damaged skin tissues. Conventional topical delivery will present some problems, such as insufficient penetration of the stratum corneum and insufficient diffusion into fibrotic and/or biofilm wound surfaces [329,330,331]. Some of these limitations can be addressed by microneedle arrays, which can transport EVs directly into deeper layers of the epidermis and dermis with minimal pain and tissue damage at the microscale size [332,333,334]. Probiotic-EVs could potentially be embedded in the microneedles that are made of polymers like hyaluronic acid, gelatin, silk fibroin, or polyvinyl alcohol (PVA), which are dissolvable, biodegradable, or hydrogel-forming [335,336,337]. After delivery into tissue, these systems could allow gradual release of EV cargo into the local wound microenvironment. The use of microneedle-based delivery could be beneficial, especially for diabetic wounds and very inflamed tissues, to enhance EV penetration and retention and decrease dosing frequencies. Microneedle-assisted delivery has not been directly shown for probiotic EVs in chronic wound models so far, and is considered a potential method of delivery based on extended EV and nanomedicine platforms.
3. **Scaffold:** Bioactive scaffolds and nanofiber-based wound dressings are other methods that are able to retain EVs for a longer period of time and provide a scaffold for spatially organized tissue regeneration. Nanofiber structures, such as electrospun nanofibers, porous scaffolds, multilayer matrices, and ECM-mimetic biomaterials, can be designed to encourage cells to infiltrate, maintain moisture levels, promote angiogenesis and/or allow for a step-wise release of EVs over time [338,339]. The composition of the scaffold, fiber diameter, surface charge, and porosity of the scaffold can all affect the efficiency of loading EVs, release from the scaffold, and uptake by cells in wound tissue [308,340,341]. Another property of some of the scaffolds is their use of antibacterial materials or oxygen-releasing or conductive biomaterials to further enhance the ability to regenerate in chronic wound environments [342,343,344]. Scaffold-assisted systems might allow for a longer period of therapeutic contact at the wound surface and increased structural support, as compared to direct EV injection. For the moment, there are no direct studies on the scaffold-assisted delivery of EVs derived from probiotics in chronic wound models, and the proposed use is thus extrapolated from general EV and biomaterial studies.
4. **ROS-sensitive material:** Recently, ROS-sensitive materials or linkers have been identified as potential platforms for the controlled delivery of EVs in the oxidative wound environment. Since chronic non-healing wounds are associated with an excessive amount of ROS, several biomaterial systems have been created where the cargo of EVs is released preferably under oxidative conditions [8,10,345]. These systems frequently involve ROS-sensitive chemical groups like thioketal bonds, boronic ester-containing polymers, peroxide-sensitive nanoparticles, or oxidation-degradable crosslinkers, which have the ability to break down their structures in the presence of higher ROS levels [346,347,348]. In general, in ROS-responsive delivery systems, sequential escalations of oxidative stress may cause degradation of the biomaterial and subsequent local EV release [349,350,351]. This approach could potentially be used to selectively improve therapeutic delivery in areas of high oxidative damage and maintain redox signaling. Notably, ROS-responsive systems also open up opportunities for more spatially controlled and sustained redox modulation than with conventional passive delivery systems. However, the application of probiotic-derived EVs in chronic wounds via ROS-responsive delivery is still an emerging strategy and needs to be validated directly.

A series of biomaterial-assisted delivery approaches together highlight the potential for incorporating probiotic-EVs into more advanced approaches for regenerative medicine rather than delivering them as a stand-alone vesicle suspension. Biomaterial-assisted systems could potentially enhance the therapeutic potential of probiotic-derived EVs in the challenging chronic wound microenvironment, as they can help enhance EV stability, retention in the tissues, penetration depth, and local release kinetics. However, the majority of these benefits stem from general EV and biomaterial research and still need to be systematically verified for probiotic-derived EVs in the context of chronic wound treatment.

### 6.3. Targeted Redox Modulation in Wound Microenvironments

**Figure 4:**
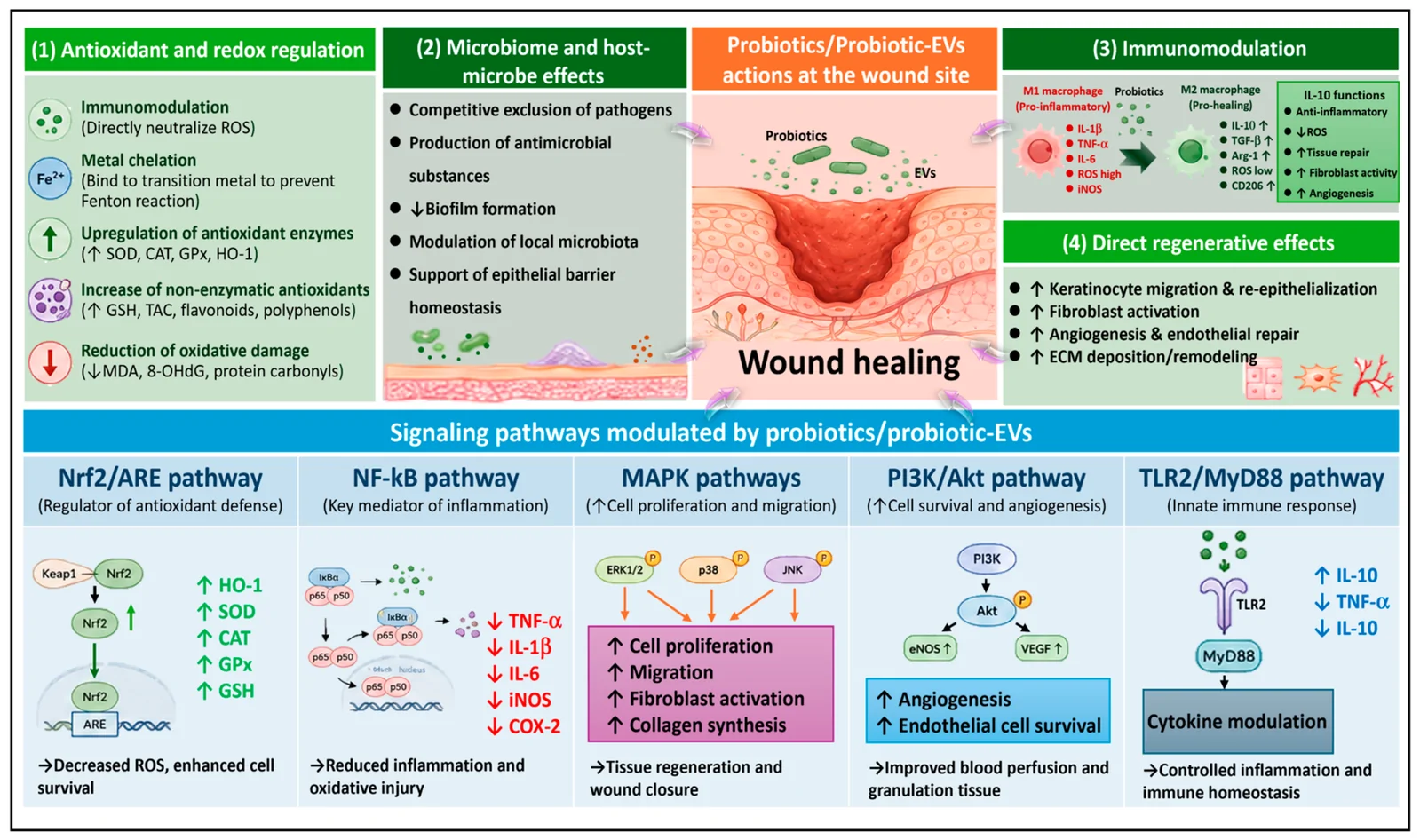
Proposed mechanistic framework by which probiotics and probiotic-derived extracellular vesicles regulate redox homeostasis, immune responses, microbiome-associated processes, and tissue repair during wound healing. Schematic overview of the interconnected mechanisms through which probiotics and probiotic-derived extracellular vesicles (EVs) may influence the wound microenvironment. In chronic wounds, excessive reactive oxygen species (ROS) accumulation, persistent inflammation, impaired angiogenesis, mitochondrial dysfunction, dysregulated immune responses, and microbial imbalance or persistent pathogenic colonization can collectively contribute to delayed tissue repair. The proposed framework organizes probiotic and probiotic-EV activity into four interconnected mechanistic domains: antioxidant/redox regulation, immunomodulation, microbiome- and host–microbe-associated effects, and direct regenerative effects on wound-resident cells and tissues. These activities may include attenuation of excessive ROS, enhancement of endogenous antioxidant defenses, modulation of inflammatory signaling and macrophage polarization, antimicrobial and barrier-supportive effects, modulation of local microbial interactions, promotion of angiogenesis, and support of extracellular matrix remodeling and re-epithelialization. At the molecular level, probiotic-derived bioactive factors and EV cargo may influence redox- and inflammation-sensitive signaling pathways, including Nrf2/ARE, NF-κB, MAPK, PI3K/Akt, and TLR2/MyD88, although the level of direct experimental support differs among pathways and cellular targets. Downstream effects may include fibroblast-associated matrix remodeling, keratinocyte migration and re-epithelialization, endothelial cell survival and angiogenesis, collagen deposition, and a shift from pro-inflammatory M1-like macrophage states toward pro-repair M2-like phenotypes. Importantly, the figure integrates experimentally supported mechanisms with mechanistically plausible interactions inferred from related probiotic- and EV-based studies. In particular, antimicrobial, barrier-protective, and host–microbe signaling effects should be distinguished from direct remodeling of the chronic wound microbiome, for which EV-specific evidence remains comparatively limited. Emerging engineering approaches, including selective cargo enrichment and stimuli-responsive biomaterial-assisted delivery, may further enhance the spatial and temporal control of probiotic-EV activity within oxidative wound environments. These strategies represent promising directions for developing redox-responsive probiotic-EV therapeutics but require further mechanistic and preclinical validation.

Probiotic-derived EVs may influence wound repair through mechanisms extending beyond direct ROS scavenging. Existing data suggest that such vesicles can influence the wound microenvironment, including redox, inflammatory, microbial, and regenerative processes that are interconnected. Conceptually, the actions of probiotics and probiotic-derived EVs can be organized into four interconnected mechanistic domains: (i) antioxidant and redox-regulatory effects, involving direct or indirect attenuation of excessive ROS and reinforcement of endogenous antioxidant defenses; (ii) immunomodulatory effects, including regulation of inflammatory signaling and macrophage phenotypes; (iii) microbiome- and host–microbe-associated effects, including antimicrobial activity, interference with pathogen colonization or biofilm-associated processes, modulation of local microbial interactions, and support of epithelial barrier homeostasis; and (iv) direct regenerative effects on wound-resident cells, including keratinocyte migration and re-epithelialization, fibroblast-associated matrix remodeling, and endothelial repair and angiogenesis (Figure 4). These domains are not mutually exclusive, but rather, microbial, immune, redox, and regenerative processes interact in a dynamic manner within the wound microenvironment.

Importantly, the strength of evidence supporting these mechanistic domains is not uniform. Several effects of probiotic-derived EVs on macrophage phenotype, keratinocyte responses, endothelial function, angiogenesis, and wound closure have been supported by direct experimental evidence in wound and inflammatory skin models. In contrast, the microbiome-related impacts of probiotics are widely acknowledged, while the direct evidence of probiotic-EVs altering the microbial ecology or composition of chronic wounds is relatively sparse. This means that antimicrobial, barrier-protective, and host–microbe signaling effects mediated by EVs should not necessarily be viewed as direct evidence of microbiome remodeling. Likewise, the proposed changes in fibroblast redox homeostasis, mitochondrial regulation, and engineered redox targeting are mechanistically reasonable but less directly demonstrated. Probiotic-derived EVs and other probiotic-associated signals contain proteins, lipids, metabolites, and regulatory nucleic acids that can impact redox- and inflammation-sensitive pathways, including Nrf2/ARE, NF-κB, MAPK, PI3K/Akt, and innate immune signaling pathways, as depicted in Figure 4 [36].

This multi-level mechanism of action is especially pertinent to chronic wounds, where ROS can accumulate, mitochondria can malfunction, inflammation may persist, vasculature may be compromised, microbial dysbiosis or persistent pathogenic colonization may occur, and intercellular communication may be disrupted; these events may all occur at the same time and may reinforce one another [352]. Probiotic-derived EV activity thus could be better conceptualized in an integrated redox–immune–microbial–regenerative paradigm, where different aspects might be supported by varying degrees of experimental data. In the following subsections, the cell-based and redox-regulatory elements of this framework in the fibroblast, keratinocyte, macrophage, and vascular compartment are critically discussed, separating out experimental data from mechanistic inference and proposed engineering strategies. For clarity, evidence in each subsection is categorized according to its experimental source, including direct probiotic-derived EV studies, live-probiotic or postbiotic studies, mammalian or other non-probiotic EV studies, and broader antioxidant or biomaterial literature. Evidence from the latter categories is considered only indirect mechanistic evidence and is not interpreted as direct evidence of probiotic-EV activity.

1. **Fibroblasts:** Fibroblasts are the main cells involved in wound repair, deposition of extracellular matrix (ECM), synthesis of collagen, wound contraction, and wound remodeling. Continued oxidative stress can induce senescence of the fibroblasts and reduce their ability to proliferate, migrate, and produce matrix, which impairs the development of granulation tissue and delays wound closure [353]. Emerging preclinical evidence indicates that probiotic-derived EVs can improve the oxidative and inflammatory wound microenvironment and promote tissue remodeling. In cutaneous wound models, MVs produced from *L. reuteri* improved wound repair and collagen deposition, with a reduction in pro-inflammatory macrophage activation and oxidative stress [92], and probiotic MV-based hydrogel application also improved dermal remodeling and collagen organization and deposition [93,95]. More recently, MVs derived from *L. reuteri* were found to directly decrease intracellular ROS and apoptosis under diabetic wound-like conditions and, when added to an inflammation-responsive hydrogel, to increase organized collagen deposition and myofibroblast-associated α-SMA expression in vivo [96]. These observations imply that probiotic-EVs can create a favorable microenvironment for fibroblast-mediated repair, but the direct mechanistic evidence of redox restoration caused by probiotic-EVs in dermal fibroblasts remains scarce.

In a mechanistic sense, probiotic-EV-mediated redox regulation may be both directly cytoprotective and indirectly mediated through remodeling of the wound immune microenvironment. *L. reuteri* MVs containing 3-hydroxypropionaldehyde (3-HPA), for example, reduced oxidative stress and stabilized mitochondrial membrane function in pro-inflammatory macrophages, promoting their transition toward an anti-inflammatory phenotype [92]. This mechanism has not yet been directly shown in fibroblasts, but the subsequent decrease in inflammatory and oxidative stress may have indirect beneficial effects on the survival and matrix remodeling of fibroblasts. Results of non-EV probiotic studies support activation of Nrf2/HO-1 antioxidant signaling, such as live *L. plantarum* supplementation leading to increased Nrf2-mediated antioxidant responses in a non-wound cardiovascular model [354]. However, direct activation of this pathway by probiotic-derived EVs in dermal fibroblasts remains to be established.

EV cargo may provide an additional route for modulating fibroblast responses. LGG-derived EVs have been demonstrated to deliver miR-21-5p to target cells in the wound microenvironment and induce reparative activities [94], and miR-21 has been associated with fibroblast migration in wound healing. In other cellular systems, EV-associated regulatory RNAs have also been related to mitochondrial bioenergetics and redox homeostasis [355,356]. These observations collectively offer a rationale for future engineering strategies that will enable the enrichment of EVs derived from probiotics with redox-active or fibroblast-regulatory cargo. Other broad EV studies show that EVs are able to transport antioxidant enzymes, such as catalase, superoxide dismutases, glutathione peroxidases, peroxiredoxins, and thioredoxin [163,351]. The presence and functional delivery of these antioxidant enzymes have, however, not been systematically established in probiotic-derived EVs. In this context, engineering probiotic-derived EVs to increase antioxidant enzyme content or to enable the delivery of regulatory RNAs that reinforce cytoprotective pathways like Nrf2 is a promising, but largely conceptual, approach to better endow fibroblasts with resistance to oxidative injury and a capacity for controlled ECM remodeling in chronic wounds.

2. **Keratinocytes:** Keratinocytes are key effector cells in re-epithelialization and epidermal barrier restoration during wound healing. Oxidative stress can affect keratinocyte proliferation, migration, cytoskeletal organization, mitochondrial function, and stress-responsive signaling, which would result in delayed epidermal regeneration and wound closure [357,358,359,360]. Recent original studies provide direct evidence that probiotic-derived EVs can protect keratinocytes and enhance their reparative functions. EVs from *L. rhamnosus* GG stimulated the proliferation and migration of keratinocytes in vitro and accelerated re-epithelialization in a full-thickness skin wound model, with the EV-derived miR-21-5p playing a key role in these effects [94]. More recently, MVs derived from *L. reuteri* were found to decrease intracellular ROS and apoptosis in HaCaT keratinocytes under diabetic wound-like conditions, maintain mitochondrial morphology and membrane potential, restore impaired keratinocyte migration, and activate Nrf2/HO-1-mediated antioxidant signaling [96].

Further evidence for EVs derived from probiotics in regulating oxidative and inflammatory responses in keratinocytes was provided by studies of inflammatory skin models. EVs from *Akkermansia muciniphila* reduced the inflammatory response of keratinocytes by targeting the TLR4/NF-κB signaling pathway with miR-21-5p, and restored damaged skin barrier integrity in the atopic dermatitis model, while EVs from *L. plantarum* directly decreased ROS levels in inflammatory keratinocytes and improved psoriasis-like skin pathology in the model [361,362]. While these results were from inflammatory skin disease models and not from wound models, they do directly indicate that EVs derived from probiotics can affect redox and inflammatory signaling of keratinocytes.

In contrast, there are some proposed mechanisms that are indirect or not fully proven in keratinocytes. For instance, antioxidant effects associated with *L. plantarum* have been described with cell-free probiotic supernatants in wound-related models [99], and redox-regulatory effects have been reported for *L. plantarum*-derived EVs in macrophages in a non-wound acute lung injury model [224]. These findings provide indirect mechanistic support but do not directly establish EV-mediated redox protection of wound keratinocytes. As such, these results should be interpreted as support for the mechanism of action of probiotic-EVs, not direct evidence of probiotic-EV action in keratinocytes.

Biomaterial-assisted delivery could also potentially improve the therapeutic performance of probiotic-EVs in the epidermis from an engineering point of view. Recent wound-healing studies have demonstrated that the incorporation of probiotic membrane vesicles into hydrogels can enhance local retention and tissue repair [93,95], and an inflammation-responsive DNA hydrogel released *L. reuteri*-derived MVs in response to ROS, promoting keratinocyte migration and accelerating healing of diabetic wounds [96]. These findings support ROS-responsive or otherwise stimuli-responsive delivery systems as promising strategies for improving the spatial and temporal delivery of probiotic-EVs to damaged epidermis. More advanced ideas such as EV delivery through dissolvable microneedles or engineered EVs specifically designed for the epidermis should at this stage be expressed as future directions, unless directly supported by experimental studies.

3. **Macrophages:** One of the key connections between oxidative stress and chronic inflammation and poor tissue repair is the dysregulation of macrophages. In chronic wounds, macrophages are continually activated in a pro-inflammatory state, leading to high levels of ROS, reactive nitrogen species, proteases, and inflammatory cytokines, while failure to transition to reparative macrophages is associated with failure of inflammatory resolution and delayed wound healing [363,364]. Importantly, direct evidence from wound models supports macrophages as a major cellular target of probiotic-derived vesicles. Membrane vesicles from *L. reuteri* decreased pro-inflammatory macrophages and facilitated an anti-inflammatory phenotypic switch in mucosal and cutaneous wounds [92]. Mechanistically, MVs decreased oxidative stress in activated macrophages, and the metabolite 3-hydroxypropionaldehyde, associated with vesicles, decreased mitochondrial permeability and stabilized mitochondrial membrane potential and was thus directly connected to macrophage phenotypic reprogramming and wound repair [92].

Complementary evidence further supports this immunomodulatory effect. EVs derived from *L. plantarum* induced M2-like polarization of macrophages and decreased the expression of the M1 marker HLA-DRα in an inflammatory situation in human skin organ culture, while also upregulating the anti-inflammatory cytokine IL-10 [365]. More recently, an inflammation-responsive hydrogel loaded with vesicles derived from *L. reuteri* facilitated M2 macrophage polarization and wound healing in diabetic wounds, which are associated with high oxidative stress and prolonged inflammation [96]. These results represent direct evidence that vesicles from probiotics are able to modulate macrophage phenotypes and that, in the case of MVs from *L. reuteri*, mitochondrial redox modulation is directly related to macrophage reprogramming during wound healing.

In addition to these observations, there is general indirect evidence for mechanisms of action of EVs in macrophage immunometabolism, as indicated by the effect on mitochondrial activity, oxidative phosphorylation, glycolysis, and other immunometabolic programs in the context of bacterial EVs or non-wound probiotic EVs [212,366]. However, the extent to which these mechanisms operate specifically in macrophages within chronic wounds remains incompletely defined. The engineering delivery of EVs derived from probiotics, selectively enriched with redox-active or immunoregulatory cargo, or integrated into stimuli-responsive delivery platforms, could potentially improve pro-resolving macrophage reprogramming. These engineered strategies are still in their infancy and would need to be directly tested in chronic wound models to be able to determine the therapeutic benefit of using engineered EVs versus native probiotic EVs.

4. **Angiogenesis and mitochondrial oxidative homeostasis:** Impaired angiogenesis and reduced tissue oxygenation are major pathological features of chronic wounds, particularly diabetic and ischemic wounds [56,367]. In these scenarios, endothelial dysfunction is tightly associated with oxidative stress and disrupted mitochondrial homeostasis. While transient or low levels of mitochondrial ROS can be important in physiological angiogenic signaling, excess or chronic mtROS can cause endothelial dysfunction, decrease nitric oxide bioavailability, and cause problems with endothelial migration and vascular homeostasis [368,369]. Thus, restoration of endothelial redox balance is likely to be important for effective neovascularization.

Today, direct preclinical evidence exists for the pro-angiogenic function of probiotic-derived vesicles in wound repair. *L. rhamnosus* GG-derived EVs enhanced HUVEC proliferation and migration, promoted endothelial tube formation, and increased wound tissue expression of VEGF-A and HIF-1α, accompanied by increased angiogenesis in a full-thickness skin wound model [94]. Likewise, membrane vesicles derived from *L. reuteri,* when added to a hydrogel formulation, stimulated endothelial cell proliferation, migration and new vessel formation in vitro, and enhanced vascularization in vivo, as evidenced by increased levels of VEGF/CD31 markers [95]. More recently, MVs derived from *L. reuteri* have been shown to directly decrease the accumulation of ROS in HUVECs under conditions mimicking diabetic wounds and to increase the angiogenic response and neovascularization in diabetic wounds upon delivery via an ROS-responsive hydrogel [96].

These results provide evidence that probiotic-derived vesicles have the ability to directly promote endothelial repair and angiogenesis, albeit with only partially understood intracellular modes of action. Indirect mechanistic support from wider endothelial redox studies, such as general antioxidant and non-probiotic EV literature, suggests that mitochondrial redox signaling may regulate Akt, ERK/p38, eNOS/NO, and other redox-sensitive signaling networks involved in VEGF-dependent migration, angiogenic sprouting, mitochondrial respiration, and endothelial survival [368,369]. However, direct evidence that probiotic-derived EVs activate these specific mitochondrial pathways in wound endothelial cells is still limited. Thus, at this time, PI3K/Akt-, MAPK-, Nrf2-, or mitochondrial OXPHOS-related mechanisms should be regarded as mechanistically plausible, but not necessarily fully established.

From an engineering perspective, spatial and temporal control of probiotic-EV activity may further improve endothelial redox regulation. The feasibility of ROS-triggered vesicle release in a high-ROS microenvironment has already been demonstrated in a diabetic wound model using a ROS-responsive hydrogel [96]. Other more sophisticated approaches, including the use of peptides to target EVs to mitochondria, hypoxia-responsive regulatory circuits, or selective enrichment of EV cargo for targeting endothelial mitochondrial metabolism, are far from concrete and need to be tested directly in vivo. Taken together, existing evidence indicates that EVs derived from probiotics possess multifunctional properties as regulators of vascular repair, and that programmable redox-targeted EVs provide a potential direction for the future, but not a current therapeutic pathway.

## 7. Challenges, Translational Barriers, and Future Directions

### 7.1. Technical and Translational Challenges

Although the use of probiotic-derived EVs has recently been of growing interest as redox-regulatory therapeutics, a number of key technical and translational hurdles remain before the next step towards clinical use.

1. **Standardization, isolation variability, and cargo heterogeneity:** The main methodological and biological sources of variability that impact the reproducibility, standardization, and translational development of EVs from probiotics are listed in Table 7. The lack of standardized methods in the isolation, purification, and characterization of EVs is at present one of the major challenges. Various methods of ultracentrifugation, filtration, precipitation kits, density gradients, or chromatography-based methods are used in different studies, leading to significant differences in vesicle purity, yield, size distribution, and content of vesicles [370,371]. Significantly, there are differences in the efficiency of recovery among these various methods, as well as differences in selectivity for different subpopulations of EVs and differences in the extent to which non-vesicular proteins, nucleic acids, or other bacterial components are co-isolated. Therefore, discrepancies among these studies might be attributed to methodological inconsistencies instead of intrinsic biological variability between EVs from different probiotics.

This also becomes more complicated in the field of bacterial EV research, as the vesicles produced by bacterial strains vary greatly depending on when they are collected in the culture growth phase, which culture medium they are grown in, the level of oxygen, exposure to stress, or nutrient availability [372,373]. These biological and culture-dependent factors lead to significant cargo heterogeneity, which includes EV-associated proteins, lipids, metabolites, and nucleic acids. Different conditions may lead to different cargo profiles and biological potencies of EVs, even when they are produced from the same probiotic species. Therefore, probiotic-EVs obtained in different laboratories may also vary significantly in terms of their biological activity and physicochemical characteristics, making it difficult to compare the results of the different studies.

In addition, currently available characterization techniques—including nanoparticle tracking analysis (NTA), electron microscopy, proteomics, lipidomics, and RNA sequencing—still lack universally accepted standards specific to microbial EV systems. This restriction is especially important when comparing therapeutic studies, as variations in EV isolation, content, and characterization could impact dose normalization, mechanistic analysis, and therapeutic outcomes. Such heterogeneity results in serious challenges to future large-scale manufacturing and clinical translation, as well as challenges to mechanistic reproducibility. Therefore, the standardization of reporting of the bacterial strain, culture conditions, isolation and purification protocols, EV characterization, cargo, and biologically relevant potency assays will be key to advancing inter-study comparability and batch-to-batch reproducibility before the translation of EVs derived from probiotics into therapeutics can be achieved.

2. **Immunogenicity:** Biosafety and immunogenicity are other significant issues. Probiotic-derived EVs are derived from bacteria, which contain structurally immunogenic components like peptidoglycan fragments, lipoteichoic acid, lipoproteins, or endotoxin-associated molecules that have the ability to stimulate innate immune signaling pathways, unlike mammalian EVs [276,277,278]. While some inflammation may be desirable for antimicrobial defense or wound healing, there is potential for chronic wounds with ongoing inflammation and an immune response to become chronic and problematic if the immune system is stimulated excessively or in an uncontrolled manner. Interestingly, the immunological effect of probiotic-EVs seems to be quite dependent on bacterial species, vesicle composition, purification, dose, and route of administration [363,374]. However, even for probiotics generally regarded as safe, the cargo profile of vesicles might be greatly different based on environmental conditions [375]. Moreover, biodistribution and systemic accumulation, activation of off-target immune cells, and effects on the microbiome are not fully understood. These uncertainties underscore the importance of more thorough safety assessment, particularly if engineered probiotic-EVs, carrying exogenous proteins or synthetic regulatory RNAs or targeted surface ligands, were ever to be developed for human clinical applications.
3. **Regulatory Issues:** In addition to biological safety, regulatory and manufacturing issues are other important obstacles relating to translation. Currently, no special regulatory guidelines for EV therapeutics of probiotic origins exist. Whether these should be considered as belonging to the class of ‘biologics’, ‘postbiotics’, ‘nanomedicines’, ‘microbial products’ or ‘engineered extracellular vesicle therapeutics’ remains to be determined, and different regulatory pathways may have significantly different manufacturing/quality control requirements [149,376]. There are also practical challenges in large-scale production, as it would be technically challenging to ensure EV yield consistency, cargo composition, and functional potency between different batches. The challenge is even more complicated for engineered probiotic EVs that contain synthetic biology circuits, RNA therapeutics, and/or responsiveness to external cues, where additional factors such as genetic stability, biosafety containment, and reproducibility are involved. Furthermore, there are challenges with the storage of the vesicles and the preservation of their biological activity, and the integrity of the vesicles might be compromised over long storage times, lyophilization and/or freeze-thaw cycles [377,378]. Overall, these restrictions suggest that there is still a lot more to do in order to transform the current experimental nanovesicles into real-world applicable redox therapeutics for chronic wound management that can be produced at a large scale and approved for regulatory use, all derived from probiotics.

### 7.2. Limitations of Current Experimental Models

While probiotic EVs have emerged as a promising therapeutic strategy for wound healing, there are several key limitations in experimental designs and translational modeling that restrict the current body of evidence.

1. **Acute Wound Bias:** One problem is that, in most cases, acute wound models are used and fail to simulate the pathological complexity of chronic non-healing wounds that are found in clinical scenarios; hence, this is acute wound bias. Most of the available studies have tested probiotic-EVs in short-duration wound healing models in otherwise healthy rodents, where wound healing, even in the absence of probiotic-EVs, progresses relatively efficiently [92,93,94,95,96]. While it may seem very promising to lower ROS levels, inflammatory cytokines, and/or wound size, these decreases may not accurately reflect the chronic oxidative stress, ischemia, biofilm formation, metabolic dysfunction, vascular insufficiency, or immune dysfunction associated with chronic wounds in diabetic, obese, aging, or peripheral vascular disease patients. Consequently, many experimental systems are probably overoptimistic in their therapeutic claims and underestimate the biological hurdles encountered in the real-world wound environment.
2. **Lack of Chronic Wound Models:** A further challenge is that there is a relative lack of models that are mechanistically well-developed enough to include both chronic redox imbalance and host–microbiome interactions in a single model. Chronic wounds are not just a static pathological defect in the tissue, but a dynamic pathological site characterized by the constant interaction of various elements, including inflammatory immune cells, senescent fibroblasts, dysfunctional keratinocytes, endothelial dysfunction, microbial colonization of the wound, degradation of the ECM, and mitochondrial oxidative stress [379,380]. Most existing probiotic-EV studies, however, have been in simplified in vitro systems, or in animal experiments of relatively short duration that are not reflective of these multifaceted processes [92,93,94,95,96]. In particular, there is very limited research focused on how probiotic EVs act in a polymicrobial biofilm-associated wound, ischemic tissues, a diabetic microenvironment, or in a recurrent inflammatory condition during long-term administration. Likewise, although the concept of the microbiota–gut–skin axis is gaining traction, the evidence that orally administered probiotic EVs can lead to modulation of distal wound repair through modulation of the redox state in the host is still scanty. As a result, many of these proposed mechanisms have not been definitively validated in physiologically relevant models of disease but are only inferential.
3. **Insufficient Human Validation:** Perhaps the most important gap is the near-total lack of human validation studies. The clinical data on probiotic-EV therapeutics remain extremely scarce, especially in wound healing applications, when compared to conventional probiotics and MSC-derived EVs [148]. The majority of current information is based on cell culture studies or on early-stage animal studies; controlled human trials of safety, biodistribution, dosing, pharmacodynamics, or long-term efficacy are virtually absent.
4. **Limited Biodistribution Studies:** More importantly, the biodistribution aspects of EVs derived from probiotics after topical, oral, or injectable administration, their persistence in wound tissues, the preferred cells that internalize them, and the composition of the EVs at the physiological level are still unknown [36].
5. **Unclear Pharmacokinetics:** Pharmacokinetic and biodistribution studies are still particularly underdeveloped but are important for optimization and regulatory approval. In vivo characterization of variables like EV half-life, in vivo accumulation, clearance routes, EV cargo stability, and dose–response relationships are still too poorly understood [381,382]. In addition, the dynamics of bacterial EVs with resident microbiota, host immunity, and host metabolic pathways may vary significantly, and the biological behavior in humans may be quite different from what is seen in a controlled laboratory system [216,383].

In total, these trends underscore a key point about the existing landscape: despite the excitement in the research field of EVs from probiotics, the evidence to support their translation to clinical use is still in its infancy. Continued innovation in mechanisms, as well as more clinically relevant chronic wound models and standardized frameworks for evaluating wound models in vivo, in combination with more carefully designed human translational studies, will then be essential for future progress. While Table 7 summarizes the major sources of experimental variability and challenges in translation, Table 8 translates these challenges into a set of minimum preclinical requirements that should be addressed prior to considering probiotic-derived EVs for clinical evaluation in wound healing.

### 7.3. Future Directions in Probiotic-EV Therapeutics

**Figure 5:**
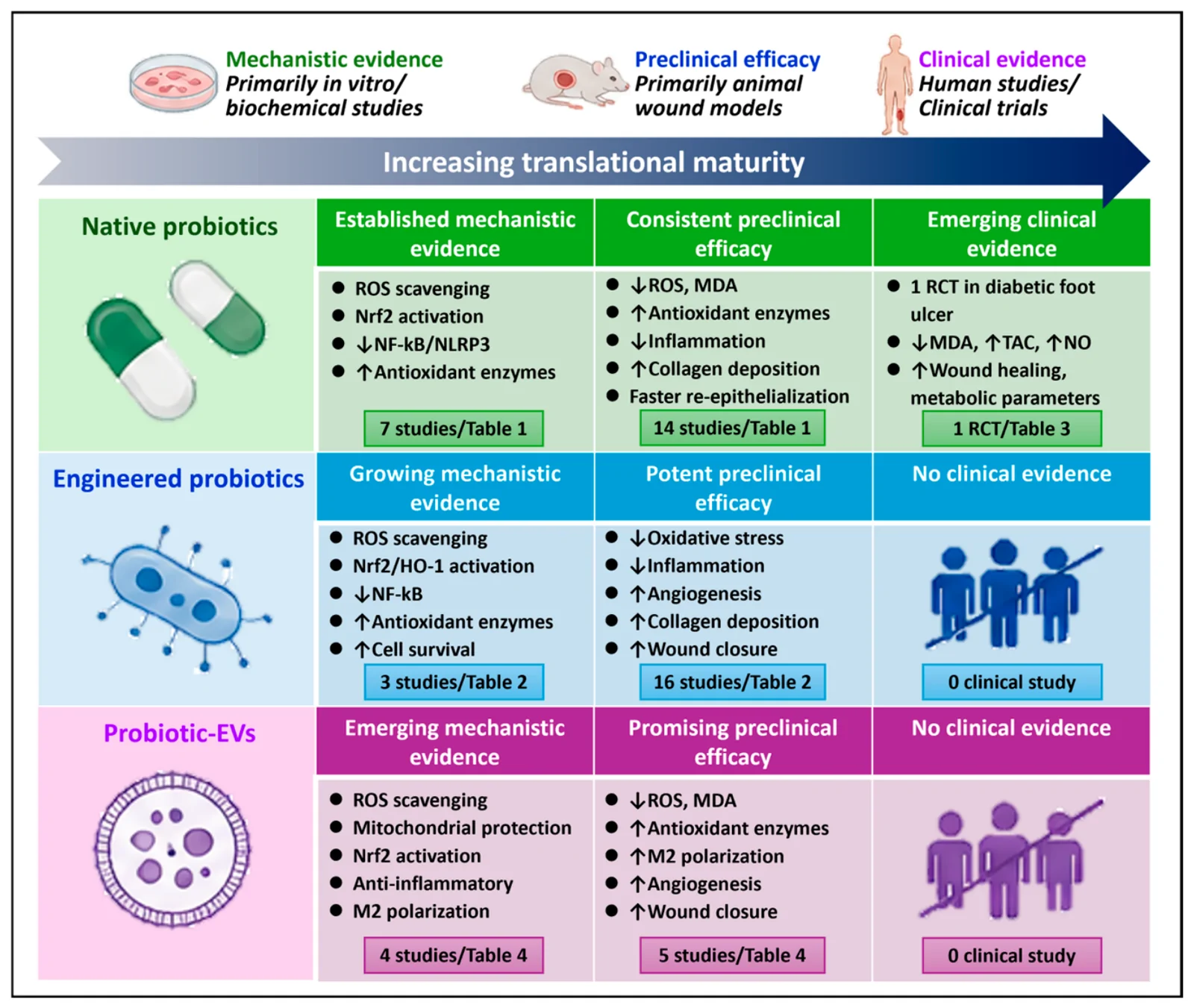
Translational maturity of probiotic-derived antioxidant strategies in wound healing. Overview of the current translational landscape of probiotic-based antioxidant strategies for wound healing, highlighting the progression from basic laboratory research to potential clinical implementation. The figure compares three major therapeutic approaches—native probiotics, engineered probiotics, and probiotic-derived extracellular vesicles (EVs)—across three levels of translational evidence: mechanistic evidence, preclinical efficacy, and clinical evidence. Among these approaches, native probiotics currently represent the most clinically advanced strategy, supported by a relatively large body of mechanistic and preclinical evidence as well as emerging human studies. Their reported benefits include ROS scavenging, activation of antioxidant pathways such as Nrf2, suppression of inflammatory signaling, promotion of M2 macrophage polarization, and support of tissue repair processes. Despite encouraging findings, clinical outcomes remain variable due to differences in probiotic strains, formulations, dosing regimens, and treatment protocols. In contrast, engineered probiotics have demonstrated increasingly sophisticated therapeutic capabilities in preclinical studies. Through genetic modification and synthetic biology approaches, these systems can be designed to enhance antioxidant activity, regulate inflammatory responses, improve angiogenesis, and provide controlled or stimulus-responsive therapeutic functions. However, despite strong mechanistic rationale and promising results in experimental models, translation into human studies remains limited, and clinical validation is still lacking. Probiotic-derived EVs represent the newest and least clinically mature platform but have attracted growing interest because of their cell-free nature, favorable safety profile, and ability to transport diverse bioactive cargo. Experimental studies suggest that probiotic-EVs may regulate oxidative stress, mitochondrial function, inflammation, macrophage polarization, angiogenesis, and tissue regeneration. Nevertheless, current evidence remains largely confined to early-stage laboratory investigations, and substantial gaps remain regarding biodistribution, manufacturing standardization, pharmacokinetics, safety assessment, and clinical efficacy. Overall, the figure highlights a translational gap between the expanding mechanistic and preclinical evidence for advanced probiotic-based platforms and their limited clinical validation. Bridging this gap will require standardized production and characterization methods, clinically relevant chronic wound models, rigorous safety and pharmacokinetic evaluation, and well-designed human trials. ↑: increase; ↓: decrease.

The study of probiotic-derived EV therapeutics is still early in its stage of development; however, it has been making strides in the last couple of years in a wide range of fields in the study of the microbiome, redox biology, biomaterials, and regenerative medicine. Although there is increasing interest and growing complexity in the antioxidative and immunomodulatory effects of probiotic-derived EV systems, engineered probiotics and probiotic-derived EV systems are still mostly confined to preclinical investigation stages, while native probiotics already have the highest level of translational maturity (Figure 5). This disproportion emphasizes one of the main dilemmas in this field: the failure to clinically validate mechanistically novel approaches. Future development will therefore need to change from experimentation in the proof-of-concept phase through to precision-based strategies based on precision that are able to adapt to the changes and variability in the oxidative, inflammatory, metabolic, and microbial aspects of chronic wounds.

1. **Precision medicine:** Precision redox therapeutics have the potential to become a large area of research for probiotic-EVs in the future. There is significant variation in the level of oxidative stress, immune activation, ischemia, microbial dysbiosis, and tissue degeneration that can occur in chronic wounds and between patients and different types of wounds [363,384]. Thus, it is very likely that next-generation probiotic-EVs will not be just antioxidants but more personalized therapeutic systems, which selectively modulate certain pathways of a pathological process. Next-generation EV therapeutics might not try to indiscriminately inhibit ROS, but rather they might target inappropriately amplified ROS signals linked to chronic inflammatory and mitochondrial disease.
2. **Gut–skin axis:** There is also a new focus area that is rapidly emerging: the microbiota–gut–skin axis [385,386]. There is now growing evidence that microbial-derived vesicles could be involved in long-range host–microbiome interactions via immune, metabolic, and epithelial signaling pathways that connect the gut with other tissues, for instance, the skin [235,387]. Probiotic EVs are nanoscale particles that interact with intestinal epithelial cells and immune cells to possibly exert an effect on systemic inflammatory or oxidative pathways in addition to wound sites [249]. This suggests that administration of probiotic-EVs via the oral route may play indirect roles in wound healing in the future via regulation of systemic redox homeostasis, macrophage activation, metabolic control and microbiota-related immune activation [212,257]. However, there is still a lot of work to be performed to understand the effects in vivo, such as biodistribution, intestinal uptake, host–microbiome interactions, and long-range signaling mechanisms.
3. **Hybrid platform:** Another interesting pathway toward going would be the use of EVs from probiotics in a hybrid therapeutic platform with advanced biomaterials or nanomedicine techniques [388,389]. Newly designed probiotic-EVs could be useful components of future multifunctional therapeutic systems that could control oxidative stress, inflammation, infection, angiogenesis, and tissue regeneration at the same time. Hybrid platforms can enhance the stability, tissue penetration, controlled release, and therapeutic duration of EVs in hostile chronic wound environments through the involvement of nanoparticles, ROS-scavenging materials, conductive biomaterials, oxygen-generating systems, or smart wound dressings [140,141,142,143,390,391]. In the future, such integrated systems might enable more coordinated modulation of the complicated pathological network that is characteristic of the failure to heal chronic wounds.
4. **MSC-EV combination:** MSC-derived EVs and probiotic-derived EVs can be used together to create a combination that could offer unique therapeutic benefits. While the strong regenerative and angiogenic properties of MSC-EVs are noteworthy, probiotic-EVs may provide complementary functions that are related to the microbiome, such as immunomodulation and redox-regulation [392,393]. Combining different abilities of vesicles of stem cell origin and microbial EVs derived from bacteria or yeast may thus be a future approach to combine the tissue-repairing properties of the vesicles with the antioxidative and immune-modulating characteristics of the microbial ones. These strategies may help in the treatment of chronic wounds that are simultaneously associated with other factors such as excess ROS, vascular impairment, microbial dysbalance and chronic inflammation. Importantly, combinational EV systems may provide wider therapeutic coverage than each individual platform.
5. **Microbiome engineering:** Progress in personalized microbiome engineering is also likely to radically change the future of probiotic-EV therapeutics. With the ever-developing multi-omics technologies, synthetic biology and artificial intelligence (AI) microbial design, future probiotic strains could potentially be engineered to enable the production of vesicles with finely tuned cargo designs that are specific to certain pathological conditions or patient populations [287,394]. In the future, possibly, engineered microbial systems could be used to produce “designer EVs” loaded with an optimized mix of antioxidant enzymes, immunoregulatory RNAs, targeting ligands, or regenerative signaling molecules which are optimized for the specific characteristics of the wound [395]. While at present many of these concepts are still speculative, they are a clear indication of the increasing overlap of microbiome sciences and precision regenerative medicine.
6. **Clinical translation:** Finally, the clinical translation of probiotic-derived EVs will need to be successful to bring the EVs to the clinical setting. In the future, successful development will require an improvement not only in biological activity, but also in the production of reproducible manufacturing protocols, standardized isolation protocols, scalable production systems, biosafety validation, and regulatory frameworks available for the development of nanovesicle therapeutics derived from microorganisms [375,376]. Together, it will be necessary to fill the existing translational gap by creating more clinically relevant chronic wound models, conducting pharmacokinetic studies, performing biodistribution analysis, and developing well-designed human trials. All these emerging directions show the potential applications of probiotic-derived EVs as an essential tool for next-generation precision therapeutic approaches for chronic wound management, especially for wounds associated with high oxidative stress, immune imbalance, and a poor wound repair process.

**Table 7:** Major sources of variability and translational limitations of probiotic-derived extracellular vesicles.

Source of Variability/Limitation	Key Factor	Potential Impact on Probiotic-EV Preparation	Implications for Wound-healing Studies and Translation	Recommended Consideration	Reference
**Isolation and Purification Variability**	Ultracentrifugation, filtration, precipitation, density-gradient separation, chromatography	Differences in EV yield, purity, size distribution, recovery of EV subpopulations, and co-isolated non-vesicular components	Reduced inter-study comparability; apparent differences in biological activity may partly arise from isolation methodology	Standardized protocols; detailed reporting of isolation/purification procedures; assessment of purity and recovery	[370,371]
**Culture-dependent Heterogeneity**	Bacterial strain, growth phase, culture medium, oxygen availability, nutrient status, environmental stress	Altered EV production, physicochemical properties, and molecular composition	EVs from the same probiotic species may exhibit different biological activities between laboratories or batches	Standardize and report strain identity and culture/harvest conditions	[372,373]
**Cargo Heterogeneity**	Variation in proteins, lipids, metabolites, and nucleic acids	Differences in antioxidant, immunomodulatory, antimicrobial, and regenerative potency	Complicates identification of active components, mechanistic interpretation, and comparison of therapeutic efficacy	Comprehensive cargo profiling and identification of cargo–function relationships	[372,373,375]
**Characterization Variability**	NTA, electron microscopy, proteomics, lipidomics, RNA sequencing and other analytical platforms	Differences in reported EV concentration, size, morphology, and cargo composition	Difficulties in comparing EV preparations and establishing reproducible quality attributes	Use complementary characterization methods and standardized reporting criteria	[370,371,372,373]
**Dose Normalization**	Particle number, protein concentration, bacterial culture volume, or other normalization approaches	Equivalent reported doses may not represent equivalent amounts of biologically active EV material	Confounds dose–response comparisons and therapeutic efficacy across wound models	Establish biologically relevant and consistently reported dosing metrics	[212,371,372,373]
**Batch-to-batch Variability**	Variation in culture, isolation, purification, and cargo composition	Inconsistent EV composition and functional potency	Challenges reproducibility, quality control, scale-up, and therapeutic manufacturing	Define critical quality attributes and functional potency assays	[372,373]
**Biological and Mechanistic Heterogeneity**	Different recipient cells, wound models, EV uptake, cargo-dependent signaling	Variable responses in macrophages, keratinocytes, fibroblasts, and endothelial cells	Limits generalization of mechanisms across wound types and experimental models	Validate mechanisms across multiple relevant cellular and in vivo wound models	[92,93,94,95,96,365,366,375]
**Scale-up and Clinical Translation**	Manufacturing scale, purification efficiency, storage stability, quality control	Changes in EV yield, integrity, cargo, or potency during large-scale production	Difficulty achieving reproducible therapeutic products suitable for clinical use	Develop scalable GMP-compatible production, storage, release criteria, and potency testing	[149,371,372,373,376,377,378]

Abbreviations: EV: extracellular vesicles; NTA: nanoparticle tracking analysis.

**Table 8:** Proposed minimum preclinical requirements before clinical translation of probiotic-derived EVs for wound healing.

Preclinical Domain	Minimum Requirement	Suggested Minimum Evidence Before Clinical Translation
**Production standardization**	Standardized culture conditions	Defined probiotic strain and reproducible culture medium, growth phase, oxygen conditions, culture duration, and harvest conditions across batches
**EV preparation**	Standardized isolation and purification	Reproducible isolation/purification workflow with documented EV recovery, purity, and removal/assessment of non-vesicular contaminants
**EV identity and quality**	Size, concentration, and morphology characterization	Reproducible particle-size distribution and concentration together with morphological confirmation using complementary characterization methods
**Molecular composition**	Protein, RNA, lipid, and metabolite cargo profiling	Representative molecular profiling with assessment of cargo consistency across independent production batches
**Microbiological safety**	Sterility and contaminant testing	Absence of viable parental bacteria and unintended microbial contamination, with assessment of relevant immunostimulatory bacterial contaminants/components
**Biological potency**	Mechanism-relevant potency assay	Reproducible quantitative assay linked to the proposed wound-healing mechanism, such as redox modulation, immune regulation, keratinocyte migration, or angiogenic activity
**Dose optimization**	Dose-response analysis	Demonstration of dose-dependent biological activity and definition of an effective and tolerable dose range using a clearly reported dose-normalization method
**Pharmacology**	Biodistribution and persistence	Wound retention, target-cell uptake, systemic exposure, off-target organ distribution, persistence, and clearance characterized for the intended administration route
**Safety**	Local and systemic toxicity	Assessment of local inflammation/tissue toxicity, systemic immune responses, major-organ toxicity, and clinically relevant laboratory safety parameters
**Disease relevance**	Chronic wound model validation	Therapeutic efficacy reproduced in at least one clinically relevant impaired-healing model incorporating features such as diabetes, ischemia, aging, or persistent inflammation/oxidative stress
**Infection relevance**	Biofilm or infection-model testing	Efficacy and safety evaluated in clinically relevant infected wounds and, where feasible, polymicrobial biofilm-associated wound models
**Comparative efficacy**	Comparison with standard wound care	Direct comparison with an appropriate standard-of-care or clinically relevant wound-treatment comparator, rather than vehicle control alone
**Durability**	Long-term safety and recurrence assessment	Follow-up beyond initial wound closure to assess tissue quality, delayed toxicity/immunogenicity, durability of repair, and wound recurrence

1. Abbreviations: EV: extracellular vesicles. 2. Note: These requirements represent a proposed preclinical framework derived from the translational gaps discussed in Section 7.1 and Section 7.2 and should not be interpreted as an established regulatory guideline specific to probiotic-derived EV therapeutics.

## 8. Conclusions

It is now understood that chronic wounds have been observed to exist as an imbalance of prolonged oxidative stress, persistent inflammation, and changes in regenerative signals. In this context, probiotic-EVs have emerged as a new preclinical therapeutic platform associated with the microbiome, which may have the potential to modulate redox balance, immune responses, and tissue repair. Probiotic-EVs, unlike classic antioxidants, which primarily act as ROS scavengers, have been associated with changes in various interconnected pathways implicated in the regulation of oxidative signaling, macrophage polarization, mitochondrial homeostasis, angiogenesis, and epithelial regeneration. However, at this time, many of these proposed mechanistic effects have only been studied preclinically; additional mechanistic validation is needed.

Furthermore, the recent advancements in engineering probiotic-EVs, synthetic biology, and biomaterial-assisted probiotic-EV delivery systems may increase the therapeutic capabilities of probiotic-EVs, thereby supporting the development of programmable and targeted redox nanotherapeutics for the treatment of chronic wounds. However, there are still big questions regarding EV standardization, biosafety, pharmacokinetics, and clinical validation that need to be answered. Importantly, the currently available human clinical evidence relates to live probiotic administration rather than probiotic-derived EV therapy and therefore provides only indirect clinical support for probiotic-mediated redox modulation in wound healing. At present, probiotic-derived EVs should be considered a promising preclinical platform for redox-oriented wound therapy, but their clinical utility remains unproven. In the future, interdisciplinary collaboration among the fields of microbiome research, regenerative medicine and nanotechnology, biomaterials science, and clinical wound management will therefore be neede(()) for further development.

## Data Availability

No new data were created or analyzed in this study.

## Supplementary Materials

The following supporting information can be downloaded at: https://www.mdpi.com/article/10.3390/antiox15091172/s1, Table S1: Detailed summary of intrinsic antioxidant mechanisms of probiotics in wound healing; Table S2: Detailed summary of engineering strategies enhancing probiotic-mediated antioxidant effects in wound healing.

## Abbreviations

α-SMA: alpha-smooth muscle actin; ABTS: 2,2′-azino-bis(3-ethylbenzothiazoline-6-sulfonic acid; ACG: adenylyl cyclase G; AGEs: advanced glycation end products; AhR: aryl hydrocarbon receptor; AKT: protein kinase B; AMPK: AMP-activated protein kinase; BAX: BCL-2-associated X protein; CAM: chick chorioallantoic membrane; CAT: catalase; CARD9: caspase recruitment domain family member 9; COL1A1: collagen type 1 alpha 1; COX-2: cyclooxygenase-2; CYP1A1: cytochrome P450 family 1 subfamily A member 1; DPPH: 2,2-diphenyl-1-picrylhydrazyl; DGGcm: Dextran-Gelatin-Gellan Gum composite microspheres; ECM: extracellular matrix; EGF: epidermal growth factor; EGFR: epidermal growth factor receptor; EPS: exopolysaccharide; ERK: extracellular signal-regulated kinase; EVs: extracellular vesicles; FAK: focal adhesion kinase; fn: fibronectin; FOS: fructooligosaccharide; FPG: fasting plasma glucose; FRAP: ferric reducing antioxidant power; GC-MS: gas chromatography-mass spectrometry; GelMA: gelatin methacrylate; GNP: guanosine monophosphate; GPx: glutathione peroxidase; GRAS: generally regarded as safe; GSDMD: gasdermin D; GSH: glutathione; GSR: glutathione reductase; HaCaT: human skin keratinocytes; HbA1c: haemoglobin A1c; HIF: hypoxia-inducible factors; HO-1: Heme Oxygenase-1; hs-CRP: high-sensitivity C-reactive protein; HUVEC: human umbilical vein endothelial cell line; LPO: lipid peroxidation; LPS: lipopolysaccharide; MAMPs: microbe-associated molecular patterns; MDA: malondialdehyde; MMPs: matrix metalloproteinases; MPO: myeloperoxidase; MPNs: myeloproliferative neoplasms; mPTP: mitochondrial permeability transition pore; MRSA: methicillin-resistant Staphylococcus aureus; MSC: mesenchymal stem cell; MT-1: Metallothionein-1; MVBs: multivesicular bodies; MVs: membrane vesicles; nanoSe: nano-selenium; NHDF: normal human dermal fibroblasts; NIR: near-infrared; NLRP3: NOD-like receptor family, pyrin domain containing 3; NOX: NADPH oxidase; Nrf2: nuclear factor erythroid 2–related factor 2; PDGF: platelet-derived growth factor; PEG: polyethylene glycol; PEO: polyethylene oxide; PMBV: phospholipid polymer; PTEN: phosphatase and tensin homolog; PVA: polyvinyl alcohol; ROS: reactive oxygen species; SA: salicylic acid; SCFA: short-chain fatty acids; SOD: superoxide dismutase; SPBA: succinic acid-4-(bromomethyl)phenylboronic acid; SPS: solubility parameters; TAC: antioxidant capacity; TGF: transforming growth factor; TIMPS: tissue inhibitors of metalloproteinases; TLR: toll-like receptor; TNF: tumor necrosis factor; VEGF: vascular endothelial growth factor.
