# Supplementary material for: Probiotics and Extracellular Vesicles as Redox Modulators in Wound Healing: From Microbial Therapeutics to Engineered Nanotherapeutic Strategies
Source: Antioxidants (Basel). 2026 Sep 15;15(9):1172. doi: 10.3390/antiox15091172 (PMC13603312; doi:10.3390/antiox15091172)
Supplement: Supplementary file 1 [file antioxidants-15-01172-s001.zip › antioxidants-4437846-supplementary.pdf]

Supplementary Tables

Table S1. Detailed summary of intrinsic antioxidant mechanisms of probiotics in wound healing

Probiotic	Delivery	Model	Redox modulation	Signaling pathway	Immune modulation	Wound healing outcome	Reference
L. plantarum	Probiotic supernatant (topical gel)	In vitro: A549 epithelial cell scratch and H_2O_2-induced oxidative stress; In vivo: mouse skin wound model	Oxidative stress protection ↑; cell viability ↑ (60–100% protection)	NR	IL-6 ↑ (early phase); IL-10 ↑ (late phase); inflammation modulation (early pro-inflammatory → later anti-inflammatory transition)	Wound closure ↑; re-epithelialization ↑; fibroblast proliferation ↑; collagen deposition ↑; angiogenesis ↑; granulation tissue ↑; antibacterial activity ↑	Dubey et al., 2021 [1]
L. plantarum	Purified exopolysaccharide incorporated into topical cream/ointment (0.5% w/w)	Rat burn wound model	ROS scavenging ↑ (DPPH activity ↑); metal chelation ↑; reducing power ↑ (antioxidant capacity ↑)	NR	Inflammation/ infiltration (histology) ↓	Wound closure ↑; burn contraction ↑; granulation tissue formation ↑; angiogenesis (new capillaries) ↑; fibrosis deposition ↑	Elmansy et al., 2022 [2]
L. plantarum	Live probiotic (co-culture / pretreatment with keratinocytes in vitro)	In vitro HaCaT keratinocyte model with *Malassezia furfur* infection	Nrf2 ↑ (restored from downregulation induced by M. furfur); ROS-related	AhR ↓; CYP1A1 ↓; Nrf2 ↑; NLRP3 inflammasome ↓ (Caspase-1 ↓; IL-1β ↓; IL-18 ↓)	IL-1β ↓; IL-18 ↓; Inflammation ↓; Inflammasome activation ↓	Wound closure ↑ (scratch assay); Keratinocyte migration ↑; Barrier integrity ↑ (Claudin-1 ↑; Zonulin ↑);	Fusco et al., 2023 [3]

			signaling indirectly modulated via AhR pathway			MMP-2 ↑ (tissue remodeling ↑)	
L. plantarum	Heat-killed probiotics (topical)	Mouse skin wound model	NR	CARD9-mediated signaling ↑; NF-κB–related pathway (via C-type lectin receptors) involved; MyD88-independent	Macrophage M2 polarization ↑; IL-10 ↑; chemokines ↑ (CCL5, CXCL1, CXCL2); leukocyte infiltration ↑	Wound closure ↑; re-epithelialization ↑; granulation tissue ↑; angiogenesis (CD31+ vessels ↑) ↑; myofibroblast accumulation ↑ (α-SMA ↑); growth factors ↑ (EGF, VEGF, FGF-b, TGF-β1)	Ishi et al., 2023 [4]
L. plantarum	Probiotic supernatant (topical gel)	Mouse infected skin wound model	Free radical scavenging ↑ (DPPH assay, up to ~90% inhibition); ROS ↓	NR	IL-6 ↓; IL-10 ↑; inflammation ↓	Wound closure ↑; re-epithelialization ↑; fibroblast proliferation ↑; collagen deposition ↑; angiogenesis ↑	Dubey et al., 2023 [5]
L. plantarum	Live probiotic (topical application in wound; co-culture with HUVECs in vitro)	In vitro: HUVEC scratch wound model; In vivo: rat diabetic skin wound model	NR	NLRP3 inflammasome ↓; Caspase-1 ↓; GSDMD ↓ (pyroptosis ↓)	IL-1β ↓; IL-18 ↓; inflammation ↓; pyroptosis ↓	wound closure ↑; cell migration ↑; re-epithelialization (histology)↑; pyroptosis ↓	Wang et al., 2024 [6]
L. plantarum	Postbiotics	Rat diabetic	Antioxidant	NR	IL-10 ↑; IL-6 ↓	Wound closure	Narang et

	(ethyl acetate extract, EAE) formulated as Carbopol gel	infected skin wound model	compounds present (GC-MS)		(normalized); inflammation ↓	↑; bacterial clearance ↑ (MRSA elimination); biofilm inhibition/disruption ↑; restoration of normal skin architecture ↑	al., 2025 [7]
L. plantarum	Lyophilized exopolysaccharide	In vitro: L929 fibroblast scratch wound model; in ovo CAM angiogenesis model	NR	TGF-β1/Smad2/3/4 ↑	IL-1β ↓; IL-6 ↓; iNOS ↓ (anti-inflammatory)	Wound closure ↑; fibroblast proliferation ↑; COL1A1 (collagen) ↑; angiogenesis ↑	Demir et al., 2025 [8]
L. rhamnosus	Viable probiotic (PBS immersion; 10^6 CFU/mL)	Zebrafish caudal fin amputation model under oxytetracycline exposure	ROS ↑ (restored amputation-induced ROS production)	Wnt signaling ↑ (wnt3a ↑; lef1 ↑; axin2 ↑)	Neutrophil recruitment ↓; apoptosis ↓; inflammation modulation ↑	Fin regeneration (length/area) ↑; tissue repair ↑	Wang et al., 2023 [9]
L. rhamnosus	Probiotic polydeoxyribonucleotide	In vitro HaCaT scratch wound under TNF-α-induced inflammatory condition	SOD-like activity ↑; DPPH radical scavenging ↑; oxidative stress protection ↑ (H_2O_2-induced cytotoxicity ↓)	A2A receptor-mediated FAK ↑/AKT ↑; ERK ↑; p38 ↑	Bidirectional NO regulation: NO ↑ (basal condition); NO ↓ (under LPS stimulation); anti-inflammatory effect ↑	Cell migration ↑; wound closure ↑ (scratch assay); epithelial repair ↑	Chae et al., 2025 [10]
L. reuteri	Probiotic	Rat skin	MPO ↓	NR	Inflammation	Wound	Khodaii et

	ointment	wound model	(oxidative/inflammatory enzyme ↓); antioxidant effect↑		↓; neutrophil activity ↓ (via MPO ↓)	contraction ↑; re-epithelialization ↑; epidermis formation ↑; collagen deposition ↑	al., 2019 [11]
Lactobacillus sp. Ca6	Exopolysaccharide (EPS-Ca6) topical gel (15 mg/mL in glycerol)	Rat skin wound model	DPPH radical scavenging ↑; Reducing power ↑; β-carotene bleaching inhibition ↑; Metal chelating activity ↑	NR	Inflammation ↓	Wound closure ↑(≈97% by day 14); Re-epithelialization (complete epidermal regeneration) ↑; Fibroblast proliferation ↑; Collagen deposition ↑ (hydroxyproline ↑); Angiogenesis ↑ (new blood vessel formation); Granulation tissue formation ↑	Trabelsi et al., 2017 [12]
B. bifidum	Metabiotic (cell-free supernatant/bacterial metabolites), oral gavag	Rat peptic ulcer wound model	SOD ↑; GPx ↑; antioxidant capacity ↑	NR	IL-8 ↑ (early phase); neutrophil recruitment ↑; macrophage infiltration ↑	Wound closure ↑; re-epithelialization ↑ (EGF ↑); fibroblast proliferation ↑; granulation tissue ↑; collagen	Almasyan et al., 2023 [13]

						deposition ↑(via fibroblast activity)	
B. bifidum	Live probiotic embedded in sprayable Schiff-base gelatin/cellulose hydrogel (topical, oxygen-limited microenvironment)	Rat oral ulcer model	NR	TLR2 / TGF-β1 / Smad3 (suggested, not directly validated); NF-κB ↓ (indirect evidence via TNF-α, IL-6 reduction)	TNF-α ↓; IL-6 ↓; IL-10 ↑; M2 macrophage polarization ↑(CD206 ↑, CD86 ↓); inflammation ↓	Wound closure ↑; re-epithelialization ↑; fibroblast migration ↑; collagen deposition ↑; α-SMA ↑; tissue remodeling ↑	Li et al., 2026 [14]
E. mundtii	Live probiotic cells (also tested lysate form in vitro)	In vitro L929 fibroblast scratch wound model	Antioxidant activity ↑ (DPPH scavenging ~40%); ROS ↓	NR	NR	Wound closure ↑ (max ~58.76% at 10^8 cells/ml, 30 h); fibroblast migration ↑ (scratch assay)	Fidan et al., 2023 [15]
L. mesenteroides	Probiotic lysate	In vitro HaCaT keratinocyte infected scratch wound model	Oxidative stress index ↓; total antioxidant capacity ↑; ROS ↓	NR	IL-6 ↓; inflammation ↓	Wound closure ↑ (24 h scratch assay); keratinocyte proliferation ↑; cell cycle progression ↑ (G0/G1 ↓; S, G2/M ↑); CDH1 ↑; wound healing gene expression ↑	Altves et al., 2024 [16]
S. putrefaciens	Live probiotic	Fish skin wound model	SOD ↑; CAT ↑; GSR ↔;	NR	IL-10 ↑; TGF-β1 ↑; IL-1β ↓;	Wound closure ↑; re-	Chen et al., 2020 [17]

	(dietary administration, 10^9 CFU/g feed)		peroxidase activity ↑; antioxidant system ↑		IL-8 ↓; inflammation ↓	epithelialization ↑; keratinocyte proliferation ↑ (pcna ↑); ECM synthesis ↑ (fn1α ↑); MMP9 ↑; angiogenesis ↑ (shh ↑)	
L. casei, *L. plantarum*	Probiotic metabolites–preconditioned MSC-derived conditioned medium (topical ointment)	In vitro: H_2O_2-induced oxidative stress; In vivo: mouse skin wound model	ROS scavenging ↑ (DPPH assay); MSC survival under oxidative stress (~4–5×) ↑; antioxidant genes ↑ (SOD-1 ↑, HO-1 ↑, MT-1 ↑)	HO-1 ↑; antioxidant gene upregulation (SOD-1, MT-1	Inflammation ↓	Wound closure ↑; healing time ↓ (≈7–14 days vs 20 days control); tissue repair ↑; antibacterial activity ↑	Kazemi et al., 2023 [18]
L. casei, *L. plantarum*, *multiple Lactobacilli spp.*	Probiotic-derived exopolysaccharides	In vitro fibroblast scratch wound model	ROS ↓ (DPPH scavenging ↑up to ~60%)	MMP-1/2/3/9/10 ↓; TIMP-1/2 ↑	NR	Fibroblast proliferation/migration ↑; scratch wound closure ↑ (~94–99%); collagen degradation ↓; ECM preservation ↑	Shirzad et al., 2018 [19]
L. casei, *L. gasseri*, *L. paracasei*, *B. lactis*, *S. thermophilus*	Paraprobiotics (Microbe-Associated Molecular	In vitro: 3T3 fibroblast scratch and H_2O_2-induced oxidative	ROS ↓; oxidative damage ↓; cell viability ↑ under	NR	IL-6 ↓; IL-1β ↓; inflammation ↓	Scratch closure ↑; fibroblast proliferation ↑; cell viability ↑; wound repair ↑	Michels et al., 2023 [20]

	Patterns, MAMPs)	stress	oxidative stress				
S. thermophilus, *L. plantarum*, *L. acidophilus* (pro-healing), *B. longum*, *B. infantis*, *B. breve*, *L. bulgaricus*	Probiotic lysates	In vitro keratinocyte scratch wound model	NO production ↑ (nitrite levels ↑ via NOS2 activity)	NOS2/NO pathway ↑; NOS2 expression ↑; (effect abolished by NOS2 inhibitor, aminoguanidine)	IL-6 ↑; IL-8 ↑ (associated with NOS2 activation	Re-epithelialization ↑ (S. thermophilus, L. plantarum, L. acidophilus); strain-dependent effects (some strains ↓ healing); healing impaired when NOS2 inhibited	Lombardi et al., 2019 [21]

↑: increase; ↓: decrease; ↔: no significant change; NR: not reported. *B. bifidum*: *Bifidobacterium bifidum*; *B. breve*: *Bifidobacterium breve*; *B. infantis*: *Bifidobacterium infantis*; *B. lactis*: *Bifidobacterium lactis*; *B. longum*: *Bifidobacterium longum*; *E. mundtii*: *Enterococcus mundtii*; *L. acidophilus*: *Lactobacillus acidophilus*; *L. bulgaricus*: *Lactobacillus bulgaricus*; *L. casei*: *Lactobacillus casei* or *Lacticaseibacillus casei*; *L. gasseri*: *Lactobacillus gasseri*; *L. mesenteroides*: *Leuconostoc mesenteroides*; *L. paracasei*: *Lactobacillus paracasei*; *L. plantarum*: *Lactobacillus plantarum* or *Lactiplantibacillus plantarum*; *L. reuteri*: *Lactobacillus reuteri* or *Limosilactobacillus reuteri*; *L. rhamnosus*: *Lactobacillus rhamnosus* or *Lacticaseibacillus rhamnosus*; *M. furfur*: *Malassezia furfur*; *S. putrefaciens*: *Shewanella putrefaciens*; *S. thermophilus*: *Streptococcus thermophilus*; α-SMA: alpha-smooth muscle actin; AhR: aryl hydrocarbon receptor; CAM: ovo chorioallantoic membrane; CARD9: caspase recruitment domain family, member 9; CDH1: cadherin-1; CFU: colony-forming unit; COL1A1: collagen type 1 alpha 1; CYP1A1: cytochrome P450, family 1, subfamily A, polypeptide 1; DPPH: 2,2-diphenyl-1-picrylhydrazyl; EAE: experimental autoimmune encephalomyelitis; ECM: extracellular matrix; EGF: epidermal growth factor; EPS: extracellular polymeric substances; ERK: extracellular signal-regulated kinases; FAK: focal adhesion kinase; FGF: fibroblast growth factor; fn: fibronectin; FOS: fructooligosaccharide; GC-MS: gas chromatography-mass spectrometry; GSDMD: gasdermin D; GSR: glutathione reductase; HaCaT: human skin keratinocytes; HUVECs: human umbilical vein endothelial cells; LPS: lipopolysaccharide; MAMPs: microbe-associated molecular patterns; MDA: malondialdehyde; MMPs: matrix metalloproteinases; MPO: myeloperoxidase; MRSA: methicillin-resistant Staphylococcus aureus; MSCs: mesenchymal stem cells; MyD88: myeloid differentiation primary response 88; NHDF: normal human dermal fibroblasts; NLRP3: NOD-like receptor family, pyrin

domain containing 3; PBS: phosphate-buffered saline; PCNA: proliferating cell nuclear antigen; Reuterin: *L. reuteri* -derived metabolite; ROS: reactive oxygen species; shh: sonic hedgehog; SOD: superoxide dismutase; TGF: transforming growth factor; TIMP: tissue inhibitors of metalloproteinases; TLR: toll-like receptor; TNF: tumor necrosis factor; vascular endothelial growth factor. Paraprobiotics (Microbe-Associated Molecular Patterns, MAMPs): non-viable microbial components (e.g., heat-killed bacteria, lysates, or purified cell components) that retain biological activity and exert effects by interacting with host immune receptors.

Table S2. Detailed summary of engineering strategies enhancing probiotic-mediated antioxidant effects in wound healing							
**Probiotic**	**Engineering strategy**	**Model**	**Redox modulation**	**Signaling pathway**	**Immune modulation**	**Wound healing outcome**	**Reference**
L. plantarum	Cell-free supernatant-mediated biosynthesis of silver nanoparticles (AgNPs)	In vitro scratch wound model (3T3 fibroblast/ Vero cells)	ROS ↑ (AgNP-induced oxidative stress for antibacterial effect); antioxidant activity ↑ (DPPH scavenging 9.9–52.4%)	NR	NR	Wound closure ↑ (≈96% at 72 h); fibroblast migration ↑; cell proliferation ↑	Vijayakumar et al., 2023 [22]
L. plantarum	Viable probiotic incorporated in sponge dressing + nanocurcumin (CSLNs; solid lipid nanoparticles)	Mouse infected wound model	LPO ↓; Catalase ↑; GSH ↑ (antioxidant enzymes ↑)	NR	TNF-*α* ↓; MMP-9 ↓ (inflammation ↓); VEGF ↑; TGF-β ↑	Wound closure ↑; bacterial load ↓; angiogenesis ↑; collagen remodeling ↑	Sandhu et al., 2023 [23]
L. plantarum	Live probiotic encapsulated in lyophilized polymeric microparticles (chitosan–alginate) + prebiotic (FOS)	Rat infected burn wound model	ROS-related oxidative stress ↓; FRAP (total antioxidant capacity) ↑; MDA (lipid peroxidation) ↓	NR	Inflammation/ infiltration ↓	Wound closure ↑; Infection clearance ↑; Re-epithelialization ↑; Collagen deposition ↑	Farahani et al., 2023 [24]
L. rhamnosus	Live probiotic-loaded	Mouse infected wound	ROS ↓ (·OH, H_2O_2, $\cdot O_2^-$ scavenging ↑	NR	Inflammation ↓; inflammatory	Wound closure (~65% by day 5) ↑; fibroblast	Miao et al., 2024 [25]

	pH/ROS dual-responsive sodium alginate hydrogel (SA-SPBA@L.rha)	model	~80–100%)		cell infiltration ↓	migration ↑; hair follicle regeneration ↑; antibacterial activity ↑; infection control ↑	
L. rhamnosus	Live probiotic encapsulated in multifunctional living hydrogel (PMBV-PVA hydrogel; nanoSe biosynthesized intracellularly + ceramide-coated bacteria)	Mouse diabetic infected skin wound model	ROS ↓; H_2O_2 ↓; NO ↓; nanoSe-mediated ROS scavenging	NF-κB ↓ (p-NF-κB, p65 ↓); PI3K/Akt/mTOR ↑	M2 macrophage polarization ↑ (CD206 ↑); M1 markers ↓ (iNOS ↓); IL-6 ↓; TNF-*α* ↓; IL-1β↓; inflammation ↓	Wound closure ↑; re-epithelialization ↑; collagen deposition ↑; angiogenesis ↑ (CD31 ↑, VEGF ↑); fibroblast/myofibroblast activation ↑ (*α*-SMA ↑); cell migration ↑; antibacterial (MRSA ↓); tissue regeneration (TGF-β1, EGF) ↑	Liu et al., 2025 [26]
L. rhamnosus	Live probiotic encapsulated in injectable photo-crosslinkable PEG hydrogel with calcium phosphate nanoparticles (CP-LGG@Gel; in	Rat tooth-extraction socket wound model (alveolar bone defect)	NR	TNF signaling pathway involvement (transcriptomics); cytokine–cytokine receptor interaction ↑	M2 macrophage polarization ↑ (CD206 ↑); IL-10 ↑; TNF-*α* ↓; inflammation↓	Wound closure ↑; re-epithelialization ↑; osteogenic differentiation ↑ (Runx2, BMP-2, ALP ↑); collagen deposition ↑; bone regeneration	Guo et al., 2026 [27]

	situ light-activated)					↑(BV/TV ↑, trabecular bone ↑); angiogenesis-related healing support	
L. rhamnosus	Probiotic exopolysaccharide (EPS-P14)-based electrospun nanofibrous membrane (EPS/PEO ± Cu/Ag-doped bioactive glass); no live bacteria	In vitro antibacterial and antioxidant models (DPPH, FRAP)	ROS scavenging ↑ (DPPH ~65–70%; FRAP reducing power ↑)	NR	NR	Antibacterial activity ↑ (*E. coli, S. aureus* inhibition up to ~85–98%); potential wound healing support (antioxidant + antibacterial functions)	Akhtach et al., 2025 [28]
L. rhamnosus	Live probiotic loaded in Dextran-Gelatin-Gellan Gum composite microspheres (DGGcm) biomaterial (sustained release system)	Rat diabetic infected skin wound model	NR	NR	IL-6 ↓; IL-10 ↑; inflammation ↓	Wound closure ↑; re-epithelialization ↑; fibroblast proliferation ↑ (Ki-67 ↑); angiogenesis ↑ (CD31 ↑, VEGF ↑); collagen deposition ↑; tissue remodeling ↑ (MMP-2 ↑, TGF-β ↑, α-SMA ↑)	Diao et al., 2025 [29]
L. rhamnosus	Live probiotic coated with γ-Fe_2O_3 nanoparticles	In vitro antibacterial, antibiofilm	ROS ↑ (from γ-Fe_2O_3 NPs antibacterial mechanism);	NR	NR	NR	Shingade et al., 2026 [30]

	(nanobiohybrid; γ-Fe_2O_3 NPs–LR) via electrostatic self-assembly	, antioxidant assays; MEF & Caco-2 cell lines only	antioxidant activity ↑ (DPPH scavenging ↑); SOD/CAT ↑ (implied from *L. rhamnosus* secretion); oxidative stress scavenging ↑				
L. rhamnosus	Live probiotic engineered bio-heterojunction (P-bioHJ): LG conjugated with MXene (Ti_3C_2) quantum dots + FeS + LOx; NIR-triggered system	Mouse biofilm-infected cutaneous wound model	ROS scavenging ↑; intracellular ROS ↓; SOD ↑; CAT ↑; MDA ↓; hydroxyl radical clearance ↑	NF-κB ↓; IκB*α* ↓; TNF-*α* ↓; TNF signaling modulation (RNA-seq); anti-inflammatory pathway inhibition	Inflammation ↓; TNF-*α* ↓; IL-6 ↓; anti-inflammatory effect ↑	Wound closure ↑; re-epithelialization ↑; collagen deposition ↑; angiogenesis ↑ (VEGF, CD31 ↑); bacterial load ↓; biofilm eradication ↑	Qin et al., 2024 [31]
L. reuteri	Metal-phenolic self-assembly shielded live probiotic (L. reuteri@FeTA) in hydrogel (Gel/L@FeTA)	In vitro fibroblast and HUVEC scratch wound model; in vivo: mouse skin wound model	NR	Angiogenesis-related proteins ↑ (VEGF ↑; CD31 ↑)	TNF-*α* ↓; IL-1β ↓; IL-10 ↑; TGF-β ↑ (anti-inflammatory ↑)	Wound closure ↑ (~98% day 12); fibroblast proliferation ↑; collagen deposition ↑; angiogenesis ↑; hair follicle regeneration ↑	Zhou et al., 2023 [32]
L. reuteri	Viable	Mouse	ROS ↓;	NR	Inflammation	Wound closure	Wang et al.,

	probiotic encapsulated in calcium alginate hydrogel (LR@CAH; with acid-responsive hydrogen-producing material)	diabetic wound model	oxidative stress ↓ (via glucose consumption + hydrogen production); antioxidant enzyme activity ↑		↓	↑	2025 [33]
L. reuteri	Reuterin incorporated into 3D-printed double-layer hydrogel (ACG-GC/Reu/SPS-PDMS)	In vitro (L929, HUVECs, RAW264.7); in vivo: rat skin wound model and pig infected wound model	NR	NF-κB ↓; AMPK ↓; IL-17 ↓; NLRP3 inflammasome activation; MAPK, TGF-β, PI3K-Akt, TNF, VEGF pathways ↑	IL-10 ↑; TGF-β ↑; Arg-1↑; M2 macrophage polarization ↑; TNF-*α* ↓; IL-1β ↓; iNOS ↓; inflammation ↓	Wound closure ↑; re-epithelialization ↑; fibroblast proliferation ↑; collagen deposition ↑; angiogenesis ↑; antibacterial	Feng et al., 2025 [34]
L. reuteri	Reuterin-crosslinked chitosan hydrogel (injectable, pH-responsive release system)	In vitro: NHDF fibroblasts under H_2O_2-induced oxidative stress; In vivo: rat infected skin wound model	ROS ↓; DPPH scavenging ↑ (>60%); oxidative stress–induced cell damage ↓; cell viability under H_2O_2 ↑	NR	Inflammation/infiltration ↓; TGF-β1 ↓	Wound closure (~10% faster) ↑; re-epithelialization ↑; collagen deposition ↑; angiogenesis ↑ (CD31 ↑); scar formation ↓; antibacterial activity ↑ (>99%)	Gao et al., 2025 [35]
L. casei	Probiotic	Rat	NO release	NR	Inflammation	Wound closure	Gong et al.,

	extracts + NO donor = biomimetic phage-like microparticles, further encapsulated in GO-doped hydrogel	infected skin wound model	↑; nitrosative stress ↑; intracellular ROS ↑ (in bacteria); oxidative stress ↑		↓ (neutrophil infiltration ↓); bacterial toxicity ↓	(~97–98%) ↑; collagen deposition ↑; angiogenesis ↑ (VEGF ↑); cell migration ↑; bacterial clearance ↑	2022 [36]
L. casei	Probiotic loaded silk fibroin/alginate scaffold	Mouse infected wound model	NR	Endoplasmic reticulum stress ↓	Macrophage M2 polarization ↑; inflammation ↓	Wound healing ↑; bacterial load ↓; angiogenesis ↑; collagen ratio regulation ↑; fibroblast-to-myofibroblast transition ↓; scarless healing ↑; hair follicle regeneration ↑	Dou et al., 2023 [37]
B. subtilis	Living probiotic encapsulated in metal–phenolic networks (MPNs) and loaded into KGM/XG hydrogel (KGXM-PCB@Bsubtilis)	Rat infected skin wound model	ROS ↓ (intracellular ROS scavenging ↑); antioxidant activity ↑ (DPPH / ABTS scavenging ↑)	NR	IL-10 ↑; TGF-β ↑; IL-6 ↓; iNOS ↓; inflammation ↓	Wound closure ↑ (~98.31% day 14); re-epithelialization ↑; collagen deposition ↑ (~81%); angiogenesis ↑ (CD31↑); fibroblast/endothelial cell migration ↑	Wu et al., 2025 [38]
P. pentosaceus	Live probiotic incorporated in hydrogel (C-	Rat skin wound model	MDA ↓ (54.76–98.58%); GSH ↑; TAC	COX-2 inhibition ↑ (phycocyanin-related)	Inflammation/infiltration ↓; microbiome balance ↑;	Wound closure ↑; re-epithelialization ↑; granulation	El-Dein et al., 2026 [39]

	phycocyanin-loaded whey protein nanofibril hydrogel)		↑ (antioxidant capacity ↑)		immune response modulation ↑	tissue ↑; angiogenesis ↑; collagen deposition ↑; fibroblast activity ↑	
L. fermentum	Live probiotic embedded in patch (lyophilized bacteria in alginate microbeads within gas-permeable dressing producing NO)	Rabbit ischemic and infected skin wound model	NR	NO signaling ↑ (nitric oxide-mediated wound healing process)	Inflammation/ infiltration (histology)↓	Wound closure ↑; epithelial coverage ↑; granulation tissue ↑; collagen deposition ↑; angiogenesis ↑; keratinocyte proliferation/migration ↑; antimicrobial effect ↑ (bacterial load ↓)	Jones et al., 2012 [40]

↑: increase; ↓: decrease; NR: not reported. *B. subtilis*: *Bacillus subtilis*; *E. coli*: *Escherichia coli*; *L. casei*: *Lactobacillus casei* or *Lacticaseibacillus casei*; *L. fermentum*: *Lactobacillus fermentum*; *P. pentosaceus*: *Pediococcus pentosaceus*; *L. plantarum*: *Lactobacillus plantarum* or *Lactiplantibacillus plantarum*; *L. reuteri*: *Lactobacillus reuteri* or *Limosilactobacillus reuteri*; *L. rhamnosus*: *Lactobacillus rhamnosus* or *Lacticaseibacillus rhamnosus*; *S. aureus*: *Staphylococcus aureus*; α-SMA: alpha-smooth muscle actin; ABTS: (2,2'-azino-bis(3-ethylbenzothiazoline-6-sulfonic acid)); ALP: alkaline phosphatase; AMPK: AMP-activated protein kinase; BMP: bone morphogenetic proteins; COX-2: cyclooxygenase-2; DGGcm: dextran-delatin-dellan gum composite microspheres; DPPH: 2,2-diphenyl-1-picrylhydrazyl; EGF: epidermal growth factor ; EPS: exopolysaccharide; FOS: fructooligosaccharide; FRAP: ferric reducing antioxidant power; GSH: glutathione lipid peroxidation; HO-1: heme oxygenase-1glutathione; HUVEC: human umbilical vein endothelial cells; LPO: lipid peroxidation; M1: Macrophage M1; M2: Macrophage M2; MAPK: mitogen-activated protein kinase; MDA: malondialdehyde; MEF: mouse embryonic fibroblasts; MMP: matrix metalloproteinases; MPNs: myeloproliferative neoplasms; MSC: mesenchymal stem cell; MT-1: metallothionein-1Heme Oxygenase-1; nanoSe: nano-selenium; NHDF: normal human dermal fibroblasts; NIR: near-infrared; NLRP3: NOD-like receptor protein 3; P-bioHJ: probiotic bio-heterojunction; PEG: polyethylene glycol; PEO: polyethylene oxide; PMBV:

phospholipid polymer; PVA: polyvinyl alcohol; ROS: reactive oxygen species; SOD-1: superoxide dismutase I metallothionein-1; TAC: total antioxidant capacity; TGF: transforming growth factor; TNF: tumor necrosis factor; Reuterin: *L. reuteri* -derived metabolite.

References

1. Dubey, A.K.; Podia, M.; Priyanka; Raut, S.; Singh, S.; Pinnaka, A.K.; Khatri, N. Insight Into the Beneficial Role of Lactiplantibacillus plantarum Supernatant Against Bacterial Infections, Oxidative Stress, and Wound Healing in A549 Cells and BALB/c Mice. *Front. Pharmacol.* **2021**, *12*, 728614. https://doi.org/10.3389/fphar.2021.728614.
2. Elmansy, E.A.; Elkady, E.M.; Asker, M.S.; Abdou, A.M.; Abdallah, N.A.; Amer, S.K. Exopolysaccharide produced by Lactiplantibacillus plantarum RO30 isolated from Romi cheese: Characterization, antioxidant and burn healing activity. *World J. Microbiol. Biotechnol.* **2022**, *38*, 245. https://doi.org/10.1007/s11274-022-03439-6.
3. Fusco, A.; Perfetto, B.; Savio, V.; Chiaromonte, A.; Torelli, G.; Donnarumma, G.; Baroni, A. Regulatory Ability of Lactiplantibacillus plantarum on Human Skin Health by Counteracting In Vitro Malassezia furfur Effects. *J. Fungi* **2023**, *9*, 1153. https://doi.org/10.3390/jof9121153.
4. Ishi, S.; Kanno, E.; Tanno, H.; Kurosaka, S.; Shoji, M.; Imai, T.; Yamaguchi, K.; Kotsugai, K.; Niiyama, M.; Kurachi, H.; et al. Cutaneous wound healing promoted by topical administration of heat-killed Lactobacillus plantarum KB131 and possible contribution of CARD9-mediated signaling. *Sci. Rep.* **2023**, *13*, 15917. https://doi.org/10.1038/s41598-023-42919-z.
5. Dubey, A.K.; Sharma, M.; Parul; Raut, S.; Gupta, P.; Khatri, N. Healing wounds, defeating biofilms: Lactiplantibacillus plantarum in tackling MRSA infections. *Front. Microbiol.* **2023**, *14*, 1284195. https://doi.org/10.3389/fmicb.2023.1284195.
6. Wang, X.; Li, X.; Liu, J.; Tao, Y.; Wang, T.; Li, L. Lactobacillus Plantarum Promotes Wound Healing by Inhibiting the NLRP3 Inflammasome and Pyroptosis Activation in Diabetic Foot Wounds. *J. Inflamm. Res.* **2024**, *17*, 1707–1720. https://doi.org/10.2147/JIR.S449565.
7. Narang, A.; Kaur, K.; Bedi, N.; Rajput, N.; Kaur, G.; Kaur, S. Targeting Microbial Biofilms and Promoting Wound Healing in MRSA-Infected Diabetic Rats using Postbiotics of Lactiplantibacillus plantarum. *Probiotics Antimicrob. Proteins* **2026**, *18*, 756–774. https://doi.org/10.1007/s12602-025-10565-8.
8. Demir, A.; Aslim, B. Investigation of multifaceted wound healing effect of exopolysaccharide (EPS) produced from probiotic strain Lactiplantibacillus plantarum GD2 as in vitro and in ovo. *Sci. Rep.* **2025**, *15*, 36512. https://doi.org/10.1038/s41598-025-90682-0.
9. Wang, J.; Wang, L.; Wang, Q.; Liu, C.; Zheng, L. Lacticaseibacillus rhamnosus GG enhances fin regeneration under oxytetracycline exposure via activating Wnt signaling and modulating gut microbiota. *Fish. Shellfish. Immunol.* **2023**, *142*, 109155. https://doi.org/10.1016/j.fsi.2023.109155.
10. Chae, D.; Oh, S.W.; Choi, Y.S.; Kang, D.J.; Park, C.W.; Lee, J.; Seo, W.S. First Report on Microbial-Derived Polydeoxyribonucleotide: A Sustainable and Enhanced Alternative to Salmon-Based Polydeoxyribonucleotide. *Curr. Issues Mol. Biol.* **2025**, *47*, 41. https://doi.org/10.3390/cimb47010041.
11. Khodaii, Z.; Afrasiabi, S.; Hashemi, S.A.; Ardeshirylajimi, A.; Natanzi, M.M. Accelerated wound healing process in rat by probiotic Lactobacillus reuteri derived ointment. *J. Basic Clin. Physiol. Pharmacol.* **2019**, *30*, 20180150. https://doi.org/10.1515/jbcpp-2018-0150.
12. Trabelsi, I.; Ktari, N.; Ben Slima, S.; Triki, M.; Bardaa, S.; Mnif, H.; Ben Salah, R. Evaluation of dermal wound healing activity and in vitro antibacterial and antioxidant activities of a new exopolysaccharide produced by *Lactobacillus* sp.Ca(6). *Int. J. Biol. Macromol.* **2017**, *103*, 194–201. https://doi.org/10.1016/j.ijbiomac.2017.05.017.
13. Almasyan, R.; Jafari, P.; Farjanikish, G.; Noorbazargan, H. Metabiotic extracted from Bifidobacterium bifidum modulates antioxidant capacity and inflammatory responses during peptic ulcer healing in male wistar rats: A preliminary study. *Iran. J. Microbiol.* **2023**, *15*, 102–110. https://doi.org/10.18502/ijm.v15i1.11924.

14. Li, M.; Yan, Y.; Gong, T.; Liu, X.; Liu, Y.; Zheng, B. Sprayable dynamic Schiff-base hydrogel engineers an oxygen-limited microenvironment for viable Bifidobacterium bifidum delivery and accelerated oral ulcer healing. *Biomater. Adv.* **2026**, *183*, 214766. https://doi.org/10.1016/j.bioadv.2026.214766.
15. Fidan, A.; Ugras, S. First isolation of a probiotic candidate Enterococcus mundtii from *Herniaria glabra* L. and evaluation of its wound healing activity. *FEMS Microbiol. Lett.* **2023**, *370*, fnad083. https://doi.org/10.1093/femsle/fnad083.
16. Altves, S.; Celik, F.S.; Gunes, C.E.; Bilecen, K. Exploring the therapeutic potential of Leuconostoc mesenteroides lysates in wound healing and immune modulation on keratinocyte cells. *Arch. Dermatol. Res.* **2024**, *316*, 548. https://doi.org/10.1007/s00403-024-03324-2.
17. Chen, Z.; Ceballos-Francisco, D.; Guardiola, F.A.; Esteban, M.Á. Dietary administration of the probiotic Shewanella putrefaciens to experimentally wounded gilthead seabream (*Sparus aurata* L.) facilitates the skin wound healing. *Sci. Rep.* **2020**, *10*, 11029. https://doi.org/10.1038/s41598-020-68024-z.
18. Kazemi, A.; Ataellahi Eshkoor, P.; Saeedi, P.; Halabian, R. Evaluation of antioxidant and antibacterial effects of lactobacilli metabolites- preconditioned bone marrow mesenchymal stem cells in skin lesions amelioration. *Bioorg Chem.* **2022**, *124*, 105797. https://doi.org/10.1016/j.bioorg.2022.105797.
19. Shirzad, M.; Hamedi, J.; Motevaseli, E.; Modarressi, M.H. Anti-elastase and anti-collagenase potential of Lactobacilli exopolysaccharides on human fibroblast. *Artif. Cells Nanomed. Biotechnol.* **2018**, *46*, 1051–1061. https://doi.org/10.1080/21691401.2018.1443274.
20. Michels, M.; Corneo, E.; Rocha, L.B.G.; Dias, R.; Voytena, A.P.L.; Rossetto, M.; Ramlov, F.; Dal-Pizzol, F.; Jesus, G.F.A. Paraprobiotics strains accelerate wound repair by stimulating re-epithelialization of NIH-3T3 cells, decreasing inflammatory response and oxidative stress. *Arch. Microbiol.* **2023**, *205*, 134. https://doi.org/10.1007/s00203-023-03469-0.
21. Lombardi, F.; Palumbo, P.; Mattei, A.; Augello, F.R.; Cifone, M.G.; Giuliani, M.; Cinque, B. Soluble Fraction from Lysates of Selected Probiotic Strains Differently Influences Re-Epithelialization of HaCaT Scratched Monolayer through a Mechanism Involving Nitric Oxide Synthase 2. *Biomolecules* **2019**, *9*, 756. https://doi.org/10.3390/biom9120756.
22. Vijayakumar, G.; Kim, H.J.; Rangarajulu, S.K. In Vitro Antibacterial and Wound Healing Activities Evoked by Silver Nanoparticles Synthesized through Probiotic Bacteria. *Antibiotics* **2023**, *12*, 141. https://doi.org/10.3390/antibiotics12010141.
23. Kaur Sandhu, S.; Raut, J.; Kumar, S.; Singh, M.; Ahmed, B.; Singh, J.; Rana, V.; Rishi, P.; Ganesh, N.; Dua, K.; et al. Nanocurcumin and viable Lactobacillus plantarum based sponge dressing for skin wound healing. *Int. J. Pharm.* **2023**, *643*, 123187. https://doi.org/10.1016/j.ijpharm.2023.123187.
24. Hassaninejad Farahani, F.; Moraffah, F.; Samadi, N.; Sharifzadeh, M.; Motasadizadeh, H.; Vatanara, A. Improved infectious burn wound healing by applying lyophilized particles containing probiotics and prebiotics. *Int. J. Pharm.* **2023**, *636*, 122800. https://doi.org/10.1016/j.ijpharm.2023.122800.
25. Miao, Y.; Wei, J.; Chen, X.; Shi, J.; Zhang, L.; Wang, L.; Yang, J.; Ma, L.; Duan, J. Evaluation of living bacterial therapy assisted by pH/reactive oxygen species dual-responsive sodium alginate-based hydrogel for wound infections. *Int. J. Biol. Macromol.* **2024**, *271*, 132536. https://doi.org/10.1016/j.ijbiomac.2024.132536.
26. Liu, Y.; Yang, M.; Li, Y.; Liu, Y.; Su, H.; Zhang, W.; Liu, Y.; Zhang, Q.; Wang, C. A multifunctional living hydrogel for the synergistic management of infected diabetic wounds. *Mater. Today Bio* **2025**, *32*, 101787. https://doi.org/10.1016/j.mtbio.2025.101787. Correction in *Mater. Today Bio.* **2026**, *37*, 102844. https://doi.org/10.1016/j.mtbio.2026.102844.
27. Guo, J.; Lei, W.; Li, B.; Li, K.; Li, J.; Wang, Z.; Liu, H.; Huang, C.; Shen, Y. Injectable living hydrogel as engineered biotherapeutic to promote tooth-extraction wound healing and alveolar bone regeneration. *Bioact. Mater.* **2026**, *59*, 355–369. https://doi.org/10.1016/j.bioactmat.2025.12.051.

28. Akhtach, S.; Tabia, Z.; Belkhou, R.; Mabrouk, K.E. Bacterial exopolysaccharide and metal-doped bioactive glass nanoparticles-based electrospun membranes for chronic wound healing. *Int. J. Pharm.* **2025**, *676*, 125594. https://doi.org/10.1016/j.ijpharm.2025.125594.
29. Diao, W.; Li, W.; Li, P.; Cai, X.; Xiao, Z.; Zhou, J. DGGcm Loaded with LGG for Promoting Diabetic Infected Wound Healing. *Macromol. Biosci.* **2025**, *25*, e00237. https://doi.org/10.1002/mabi.202500237.
30. Shingade, J.A.; Padalkar, N.S.; Park, J.P.; Patil, A.R. Biocompatible nanobiohybrid of maghemite nanoparticle-coated probiotics: A synergistic platform for enhanced antibacterial, antibiofilm, and antioxidant activities. *N. Biotechnol.* **2026**, *93*, 130–142. https://doi.org/10.1016/j.nbt.2026.02.009.
31. Qin, M.; Zhang, X.; Ding, H.; Chen, Y.; He, W.; Wei, Y.; Chen, W.; Chan, Y.K.; Shi, Y.; Huang, D.; et al. Engineered Probiotic Bio-Heterojunction with Robust Antibiofilm Modality via "Eating" Extracellular Polymeric Substances for Wound Regeneration. *Adv. Mater.* **2024**, *36*, e2402530. https://doi.org/10.1002/adma.202402530.
32. Zhou, C.; Zou, Y.; Xu, R.; Han, X.; Xiang, Z.; Guo, H.; Li, X.; Liang, J.; Zhang, X.; Fan, Y.; et al. Metal-phenolic self-assembly shielded probiotics in hydrogel reinforced wound healing with antibiotic treatment. *Mater. Horiz.* **2023**, *10*, 3114–3123. https://doi.org/10.1039/d3mh00033h.
33. Wang, Y.; Shi, L.; Lu, J.; Wang, F.; Zhou, Z.; Wang, Y.; Du, X.; Qin, D.; Chen, F.; Shao, D.; et al. Probiotic active gel promotes diabetic wound healing through continuous local glucose consumption and antioxidant. *J. Nanobiotechnology* **2025**, *23*, 62. https://doi.org/10.1186/s12951-025-03115-5.
34. Feng, L.; Peng, Q.; Miao, L.; Cai, C.; Tay, F.R.; Zhou, S.; Zhang, Y.; Liu, Z.; Wang, X.; Jiao, Y.; et al. "Monitor-and-treat" that integrates bacterio-therapeutics and bio-optics for infected wound management. *Bioact. Mater.* **2025**, *48*, 118–134. https://doi.org/10.1016/j.bioactmat.2025.02.001.
35. Gao, Y.; Wang, P.; Huang, S.; Zheng, Q.; Wu, S.; Huang, J.; Chen, Z.; Gao, B. Antibacterial reuterin as a multifunctional crosslinker for constructing chitosan-based hydrogels to promote infected wound healing. *Int. J. Biol. Macromol.* **2025**, *316*, 144494. https://doi.org/10.1016/j.ijbiomac.2025.144494.
36. Gong, C.; Guan, W.; Liu, X.; Zheng, Y.; Li, Z.; Zhang, Y.; Zhu, S.; Jiang, H.; Cui, Z.; Wu, S. Biomimetic Bacteriophage-Like Particles Formed from Probiotic Extracts and NO Donors for Eradicating Multidrug-Resistant Staphylococcus aureus. *Adv. Mater.* **2022**, *34*, e2206134. https://doi.org/10.1002/adma.202206134.
37. Dou, Z.; Li, B.; Wu, L.; Qiu, T.; Wang, X.; Zhang, X.; Shen, Y.; Lu, M.; Yang, Y. Probiotic-Functionalized Silk Fibroin/Sodium Alginate Scaffolds with Endoplasmic Reticulum Stress-Relieving Properties for Promoted Scarless Wound Healing. *ACS Appl. Mater. Interfaces* **2023**, *15*, 6297–6311. https://doi.org/10.1021/acsami.2c17168.
38. Wu, Q.; Lu, Z.; Wang, L.; Peng, S.; Wang, Z.; Qiu, Y.; Liao, Z.; Wang, Y.; Qin, X. Konjac glucomannan/xanthan gum hydrogels loaded with metal-phenolic networks encapsulated probiotic to promote infected wound healing. *Carbohydr. Polym.* **2025**, *353*, 123243. https://doi.org/10.1016/j.carbpol.2025.123243.
39. Negm El-Dein, A.; Soliman, T.N.; Ezzat, A.; Abd El-Fattah, M.A.; Aly, H.F.; Younis, E.A.; Flefil, N.S. Innovative Hydrogel Formulation Combining Phycocyanin and Probiotic for Enhancing Skin Regeneration and Accelerated Wound Healing: A Preclinical Investigation in Wistar Rats. *Probiotics Antimicrob. Proteins* **2026**, *18*, 2037–2055. https://doi.org/10.1007/s12602-025-10635-x.
40. Jones, M.; Ganopolsky, J.G.; Labbe, A.; Gilardino, M.; Wahl, C.; Martoni, C.; Prakash, S. Novel nitric oxide producing probiotic wound healing patch: Preparation and in vivo analysis in a New Zealand white rabbit model of ischaemic and infected wounds. *Int. Wound J.* **2012**, *9*, 330–343. https://doi.org/10.1111/j.1742-481X.2011.00889.x.
